# Chemoselective Solution- and Solid-Phase Synthesis of Disulfide-Linked Glycopeptides

**DOI:** 10.1021/acs.joc.2c01651

**Published:** 2022-10-20

**Authors:** Katreen A. F. Banisalman, Athina Polykandritou, Francis M. Barnieh, Goreti Ribeiro Morais, Robert A. Falconer

**Affiliations:** Institute of Cancer Therapeutics, Faculty of Life Sciences, University of Bradford, Bradford BD7 1DP, U.K.

## Abstract

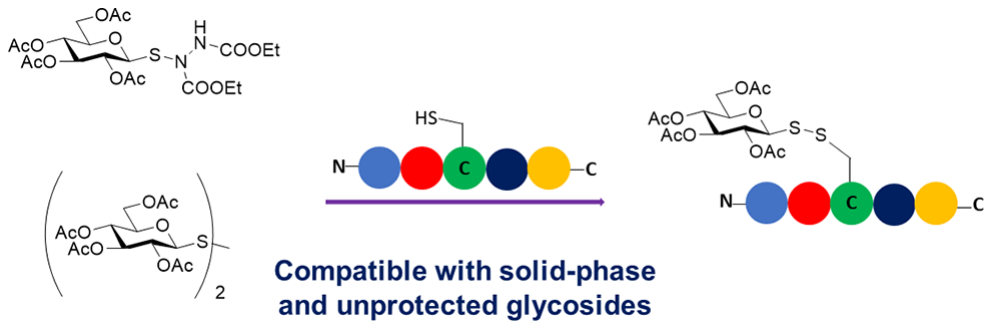

Glycosylation of peptides and proteins is a widely employed strategy to mimic important post-translational modifications or to modulate the physicochemical properties of peptides to enhance their delivery. Furthermore, glycosylation *via* a sulfur atom imparts increased chemical and metabolic stability to the resulting glycoconjugates. Herein, we report a simple and chemoselective procedure to prepare disulfide-linked glycopeptides. Acetate-protected glycosylsulfenyl hydrazines are shown to be highly reactive with the thiol group of cysteine residues within peptides, both in solution and as part of conventional solid-phase peptide synthesis protocols. The efficiency of this glycosylation methodology with unprotected carbohydrates is also demonstrated, which avoids the need for deprotection steps and further extends its utility, with disulfide-linked glycopeptides produced in excellent yields. Given the importance of glycosylated peptides in structural glycobiology, pharmacology, and therapeutics, the methodology outlined provides easy access to disulfide-linked glycopeptides as molecules with multiple biological applications.

## Introduction

The therapeutic potential of peptides has gained much interest within the drug discovery community since the first significant application, that of the use of insulin in the 1920s for the treatment of diabetes mellitus.^1,2^ Since then, there have been various strategies over recent decades to develop therapeutic peptides, particularly for the treatment of metabolic diseases and cancer, which have led to a cumulative increase in the number of peptides entering clinical trials per year since 1980.^2−4^ This interest is accelerating such that there are currently over 150 therapeutic peptides undergoing various stages of clinical trial. Despite the enthusiasm surrounding peptide drug development, the clinical success of these agents has been relatively limited compared to the volume of research reported in this field. Of the approximately 500 clinically evaluated therapeutic peptides since 1980, it is perhaps surprising that only 60 have been approved by the Food and Drug Administration (FDA) for the treatment of various diseases.^2^ This has been attributed to various factors, including poor pharmacokinetics resulting from poor metabolic stability, rapid elimination, and low bioavailability.^3^ Several strategies have therefore been developed to mitigate some of these limitations. One strategy that is often employed to improve the proteolytic stability of peptides is the capping of their N- and/or C-terminus using chemical modifications, including *N*-acetylation, *N*-amidation, and small molecule N*-* or C-terminal conjugation.^5^ Despite the improvement in metabolic stability, these modifications can increase overall hydrophobicity; however, it exacerbates the often already poor aqueous solubility exhibited by some peptides.

Glycosylation has been used as a strategy to improve the aqueous solubility of hydrophobic peptides,^6^ though this type of modification can potentially change the three-dimensional (3D) structure of the molecule and thus its biological function.^7^ Naturally occurring N- and O-linked glycosylated proteins are common examples of post-translational modifications,^8^ and several synthetic efforts have been made to mimic these transformations. Although rare, glycans connected to peptides *via* a cysteine residue have also been identified in nature,^9,10^ and synthetic *S*-glycosylation is a well-accepted strategy to mimic *O*-glycosylated congeners as it can confer resistance to glycosidase enzymes.^11,12^ Additionally, a disulfide bond at the anomeric carbon has drawn interest as an alternative tether in glycobiology.^13−15^ In glycochemistry, glycosyl disulfides have been explored either as glycosyl donors^16−20^ or as a way to access glycoproteins^21,22^ and vaccines.^23^ The growth of interest in glycosyl disulfides prompted the development of several methods for their synthesis,^24−27^ and albeit less well explored, some efforts have also been dedicated to the preparation of glycopeptides since the disulfide linkage allows for chemoselective modification of peptides and/or proteins. Also, as disulfides are flexible^28^ and can adopt conformations imposed by natural amide bonds at glycosylation sites,^29^ the glycosylation of peptides *via* disulfide bonds can be considered as a route to structural mimics of natural N-linked glycoproteins.

The synthesis of disulfide-linked glycopeptides generally focuses on the reaction of a cysteine residue with an electrophilic thiol-specific carbohydrate reagent. The use of glycoselenylsulfides as sulfur transfer reagents allows the quantitative glycosylation of proteins *via* a disulfide linkage with either protected or unprotected mono- and oligosaccharides.^14^ Glycosyl thiosulfonate esters have also been used to glycosylate proteins, but the efficiency of their preparation can be dramatically affected by the synthetic conditions, often being contaminated with the respective glycosyl dithiosulfonate ester, and they are additionally unstable under basic conditions.^17,30^ Another example includes the 5-nitropyridinylsulfenyl reagent, though this group was introduced into glucosamine in only a modest yield, with large excesses (50 mol equiv) required to produce the desired neoglycoproteins efficiently. Attempts to glycosylate peptides with unprotected disaccharides using this sulfenyl intermediate failed, however.^31^ Alternatively, it was also claimed that glycosyl sulfenic acids^32^ and sulfenamides^33^ were able to glycosylate cysteine *via* a disulfide bond, but synthesis of the former was less straightforward with the need for *meta*-chloroperoxybenzoic acid (*m*CPBA) as oxidant, followed by thermolysis of the glycosyl sulfoxides. Also, the use of these thiol-reactive synthons has not been demonstrated for the direct glycosylation of peptides, and the use of sulfenamides requires rigorous control of low temperatures.^32,33^ While these strategies have proven to be chemoselective, the issues associated with either the synthesis of the glycosyl sulfur transfer agent and the associated stability or kinetic control of the glycosylation reaction itself still present a significant challenge. A simpler strategy was required that could be applied robustly in our laboratory. In previous work, we developed a one-pot glycosyl disulfide synthesis *via* the use of an *in situ* glycosylsulfenyl hydrazine intermediate.^34^ The simplicity of this reaction encouraged us to further explore its potential application to the synthesis of disulfide-linked glycopeptides.

## Results and Discussion

**Scheme 1:**
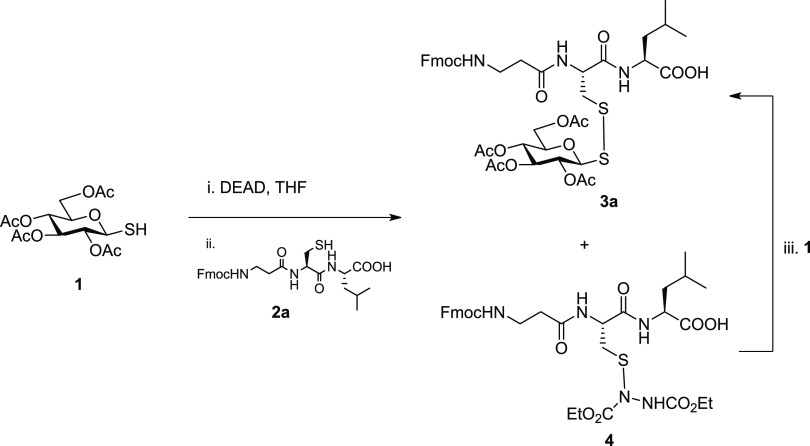
One-Pot Protocol to Prepare Disulfide-Linked Glycopeptides

We used an amine-protected tripeptide (Fmoc-βAla-Cys-Leu-OH) as a simple model peptide to investigate the glycosylation of cysteine using per-*O*-acetylated-1-thioglucose (**1**) as a representative monosaccharide. Progress of the reactions was monitored by liquid chromatography/mass spectrometry (LC/MS) for qualitative/quantitative analyses. In our previous work, unsymmetrical glycosyl disulfides were easily obtained in excellent yields when thioglucose **1** was slowly added to a solution of azo compound (*e.g.*, diethyl azodicarboxylate, DEAD), followed by an excess of reacting thiol, and the reactions were completed within 2 h in excellent yields.^34^ In this work, we decided to use thioglucose in excess. For this purpose, Fmoc-βAla-Cys-Leu-OH (**2a**) (1 equiv) was added to a previously mixed solution of per-*O*-acetylated thioglucose **1** (5 equiv) with DEAD (10 equiv). An excess of DEAD was required to avoid the formation of the symmetrical glycosyl disulfide. LC/MS analysis indicated that the reaction was complete after just 10 min: complete conversion of the peptide was observed. However, it was found that despite the glycosyl sulfenylhydrazine intermediate being in excess (5 equiv), the peptide cysteine was highly reactive with the excess DEAD so that in addition to the desired disulfide-linked glucopeptide **3a**, the cysteinyl analogue **4** was also observed in a 1:1 ratio (Scheme 1). In turn, compound **4** was completely converted into desired glucopeptide **3a** when additional thiosugar **1** was added to the reaction.

This observation prompted us to next try the glycosylation of the protected tripeptide inversely through the *in situ* activation of the cysteine followed by the conjugation of the per-*O*-acetylated thioglucose **1** under the following conditions (1.0 equiv peptide; 3.0 equiv DEAD; 5.0 equiv thiosugar; tetrahydrofuran (THF), room temperature (RT)). Despite the excess of thioglucose, we found the nucleophilicity of the cysteine residue of tripeptide **2a** to be higher than thioglucose **1** and that as a result of its reaction with the rapidly formed electrophilic thiol-specific intermediate **4**, a symmetric disulfide-linked peptide homodimer was always obtained in addition to desired glucopeptide **3a**. The kinetics of this reaction were difficult to control, and unsuccessful attempts to decrease the formation of the undesired cystine peptide dimer only led to unreacted tripeptide **2a** and poor yields of desired glucopeptide **3a**.

**Scheme 2:**
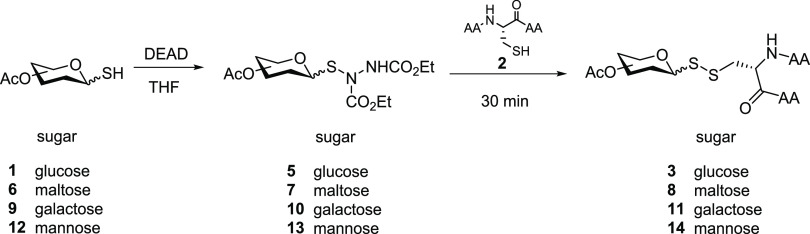
Two-Pot Protocol to Access Disulfide-Linked Glycopeptide

The equal reactivity of cysteine and thioglucose toward the hydrazine intermediate **4** (they have similar p*K*_a_ values—cysteine p*K*_a_ 8.2; thioglucose theoretical p*K*_a_ 8.4) constituted a setback to the establishment of a one-pot protocol to access disulfide-linked glycopeptides *via* oxidation with azodicarboxylates. Consequently, we decided to isolate the glucosulfenyl hydrazine intermediate **5** instead, which was prepared in excellent yields (85%) (Scheme 2) and was found to be highly stable to storage over time at RT. Dropwise addition of a diluted solution of per-*O*-acetylated thioglucose (**1**) (1.0 equiv) to a solution of DEAD (2.0 equiv) in THF allowed complete kinetic control of the reaction, which was complete instantly, and prevented the formation of the symmetric glycosyl disulfide. Having purified glucosulfenyl **5**, we reacted it (5.0 equiv) with tripeptide **2a** (1.0 equiv) in THF at RT (Table 1, entry 1). LC/MS analysis of the reaction after 30 min indicated full conversion of the peptide into desired disulfide-linked thioglucopeptide **3a**. The equivalent electrophilic thiol-specific glucosyl intermediate using diisopropyl azodicarboxylate (DIAD) as the azo source was found to be similarly susceptible to nucleophilic substitution by the cysteine of peptide **2a** and efficiently gave access to glucopeptide **3a** as the sole product cleanly within 30 min.

**Table 1:** Substrate Scope for the Synthesis of Disulfide-Linked Glycopeptides (with Per-*O*-Acetylated Sugars)

entry	sugar	peptide	product (%)^a^	ES MS found (calcd)
1	**5**	**2a** (Fmoc-βAla-Cys-Leu-OH)	**3a**^b^ (>95%)	890.3 [M + H]^+^ (889.28)
2	**5**	**2b** (Fmoc-βAla-Cys-Tyr-Leu-OH)	-b	
3	**5**	**2b** (Fmoc-βAla-Cys-Tyr-Leu-OH)	**3b**^b^^,^^c^ (>95%)	1053.3 [M + H]^+^ (1052.34)
4	**5**	**2c** (Fmoc-βAla-Cys-Lys-Leu-OH)	**3c**^b^ (>95%)	1018.4 [M + H]^+^ (1017.37)
5	**5**	**2d** (H_2_N-βAla-Gly-Cys-Leu-Tyr-Leu-OH)	**3d**^b^ (50%)	
6	**5**	**2d** (H_2_N-βAla-Gly-Cys-Leu-Tyr-Leu-OH)	**3d**^b^^,^^c^ (>95%)	1001.3 [M + H]^+^ (1000.38)
7	**5**	**2e** (H_2_N-Tyr-Thr-Gly-Phe-Leu-Cys-OH)	**3e**^c^^,^^d^ (>95%)	1065.3 [M + H]^+^ (1064.37)
8	**5**	**2f** (H_2_N-Tyr-Thr-Gly-Phe-Leu-Leu-OH)	-c^,^^d^	
9	**5**	**2g** (Fmoc-Met-Pro-Ala-Cys-Gly-Ser-Ser-OH)	**3g**^c^^,^^d^ (>95%)	619.7 [M + 2H]^2+^ (1236.34)
10	**5**	**2h** (Fmoc-βAla-Gly-Cys-Ala-Cit-Leu-His-Leu-OH)	**3h**^c^^,^^d^ (>95%)	713.7 [M + 2H]^2+^ (1425.58)
11	**5**	**2i** (Fmoc-βAla-Arg-Cys-Gly-Asn-Leu-OH)	**3i**^c^^,^^d^ (>95%)	609.5 [M + 2H]^2+^ (1217.32)
12	**5**	**2j** (Fmoc-βAla-Arg-Gly-Asn-Leu-OH)	-c^,^^d^	
13	**5**	**2l** (H_2_N-Cys-Tyr-Phe-Gln-Asn-Cys-Pro-Arg-Gly-CONH_2_)	**3l**^c^^,^^d^ (>95%)	906.6 [M + 2H]^2+^ (1810.95)
14	**7**	**2e** (H_2_N-Tyr-Thr-Gly-Phe-Leu-Cys-OH)	**8e**^d^ (>90%)	677.7 [M + 2H]^2+^ (1352.46)
15	**7**	**2g** (Fmoc-Met-Pro-Ala-Cys-Gly-Ser-Ser-OH)	**8g**^c^^,^^d^ (>90%)	762.9 [M + 2H]^2+^ (1523.46)
16	**7**	**2h** (Fmoc-βAla-Gly-Cys-Ala-Cit-Leu-His-Leu-OH)	**8h**^c^^,^^d^ (>90%)	858.0 [M + 2H]^2+^ (1713.83)
17	**7**	**2i** (Fmoc-βAla-Arg-Cys-Gly-Asn-Leu-OH)	**8i**^c^^,^^d^ (>90%)	753.6 [M + 2H]^2+^ (1505.58)
18	**7**	**2l** (H_2_N-Cys-Tyr-Phe-Gln-Asn-Cys-Pro-Arg-Gly-CONH_2_)	**8l**^c^^,^^d^ (>90%)	798.9 [M + 3H]^3+^ (2387.45)
19	**10**	**2c** (Fmoc-βAla-Cys-Lys-Leu-OH)	**11c**^c^^,^^d^ (>90%)	1018.2 [M + H]^+^ (1017.37)
20	**10**	**2g** (Fmoc-Met-Pro-Ala-Cys-Gly-Ser-Ser-OH)	**11g**^c^^,^^d^ (>90%)	618.9 [M + 2H]^2+^ (1236.34)
21	**10**	**2h** (Fmoc-βAla-Gly-Cys-Ala-Cit-Leu-His-Leu-OH)	**11h**^c^^,^^d^ (>90%)	713.4 [M + 2H]^2+^ (1425.58)
22	**10**	**2i** (Fmoc-βAla-Arg-Cys-Gly-Asn-Leu-OH)	**11i**^c^^,^^d^ (>90%)	609.4 [M + 2H]^2+^ (1217.32)
23	**13**	**2g** (Fmoc-Met-Pro-Ala-Cys-Gly-Ser-Ser-OH)	**14g**^c^^,^^d^^,^^e^ (>90%)	618.8 [M + 2H]^2+^ (1236.34)

a Conversion determined by LC/MS.

b Reaction in THF.

c 1.0 equiv DIPEA added to the reaction.

d Reaction in DMF.

e 10 equiv of **13**.

This two-pot reaction for glycopeptides is in contrast with our original report, however, in which a one-pot reaction proved successful for the synthesis of aliphatic and aromatic asymmetric glycosyl disulfides.^34^ It proved to be the best approach to obtain the desired disulfide-linked glucopeptide in a straightforward manner with excellent control of the product and no peptide-related byproducts.

Next, we focused our attention on the glycosylation of more complex peptides with unprotected amino acid side-chain functionality, such as the phenol of Tyr (Table 1, entry 2) and the primary amine of Lys (Table 1, entry 4). When glucosulfenyl hydrazine **5** was reacted with Fmoc-βAla-Cys-Tyr-Leu-OH (**2b**), no product was observed, while the reaction of **5** with Fmoc-βAla-Cys-Lys-Leu-OH (**2c**) showed complete conversion into the desired disulfide-linked glucopeptide **3c** within 30 min. We think that the reduced nucleophilicity of the cysteine residue in peptide **2b** may result from intramolecular hydrogen bonding between the phenol and thiol groups. Indeed the p*K*_a_ of a given cysteine residue can vary in proteins, ranging from 7.4 to 9.2.^35^ When DIPEA (1 equiv) was added to the reaction, however, peptide Fmoc-βAla-Cys-Tyr-Leu-OH (**2b**) was completely converted into glucopeptide **3b** within 5 min. (Table 1, entry 3). This is a clear indication that the p*K*_a_ of the cysteine residue in peptide Fmoc-βAla-Cys-Tyr-Leu-OH (**2b**) is higher than in **2a**.

The compatibility of the Lys side chain in assembling the disulfide-linked glycopeptide under these conditions encouraged us to react intermediate **5** with peptide H_2_N-βAla-Gly-Cys-Leu-Tyr-Leu-OH (**2d**) (Table 1, entry 5), in which the N-terminal is not protected. Despite the apparent need for a base to allow disulfide-glycosylation of peptides containing Tyr residues, we reasoned that the N-terminal amine might act as a base and promote the reaction. Indeed, within 30 min, glycosylation of **2d** in the absence of DIPEA led to 50% conversion of peptide **2d** into desired glucopeptide **3d**, as indicated by LC/MS analysis (Table 1, entry 5). Repetition of the reaction for the same length of time but with DIPEA promoted complete conversion of **2d** into disulfide-linked glucopeptide **3d**. This indicates that it is preferable to perform the glycosylation in the presence of a base to ensure complete and efficient conversion. While THF was a suitable solvent, DMF, which is the routine solvent of choice to dissolve longer and complex peptides in solid-phase peptide synthesis, was found to be equally efficient to allow glycosylation of the cysteine of the peptides NH_2_-Tyr-Thr-Gly-Phe-Leu-Cys-OH (**2e**) and Fmoc-Met-Pro-Ala-Cys-Gly-Ser-Ser-OH (**2g**) with **5** (Table 1, entries 7 and 9). The presence of other unprotected amino acids in the peptide sequences such as His, Cit (citrulline), Asn, and Arg (Table 1, entries 10 and 11) was found to be similarly compatible with the glycosylation conditions using sulfenyl hydrazine intermediate **5**. Peptides **3h** and **3i** were obtained efficiently and in high purity. We confirmed that no product was formed when we attempted the glycosylation with peptides **2f** (H_2_N-Tyr-Thr-Gly-Phe-Leu-Leu-OH) and **2j** (Fmoc-βAla-Arg-Gly-Asn-Leu-OH) (Table 1, entries 8 and 12) that lack Cys residues in their sequence. This demonstrates the chemoselectivity of glucosyl sulfenyl hydrazine **5** for thiol-containing residues and the compatibility to glycosylate peptides containing unprotected functional groups that range from carboxylic acids, amines, thioethers, phenols, alcohols, ureidos, imidazoles, carboxamides, and guanidines.

To further demonstrate the versatility of this approach, we glycosylated peptide **2l** (Table 1, entry 13), which contains two unprotected Cys residues. Likewise, the respective disulfide-linked di-glucopeptide **3l** was obtained as the sole product within 30 min. Furthermore, we have shown that the reaction is not limited to monosaccharides; disaccharides could also glycosylate peptides *via* this method, and glycopeptides **8e**–**8l** were efficiently obtained when per-*O*-acetylated thiomaltose derivative **7** was used as the glycosulfenyl transfer agent (Table 1, entries 14–18). This protocol is not restricted to sugars from the glucopyranoside series, as demonstrated when galactosyl analogue **10** is used for the glycosylation of peptides **2c**, **2g**–**2i** (Table 1, entries 19–22). However, the glycosylation with mannosulfenyl hydrazine **13** was less efficient. While glycopeptide **14g** was obtained in high yield (>90%) (Table 1, entry 23), the reaction with peptides **2c**, **2h**, and **2i** led to significant amounts of the cystine peptide dimer (approx. 40–50%), which is reflected in poor yields of respective glycosylated products. When compared to sulfenyl hydrazines **5**, **7**, and **10**, two-fold higher equivalents of **13** were necessary to fully convert **2g** into **14g**.

**Scheme 3:**
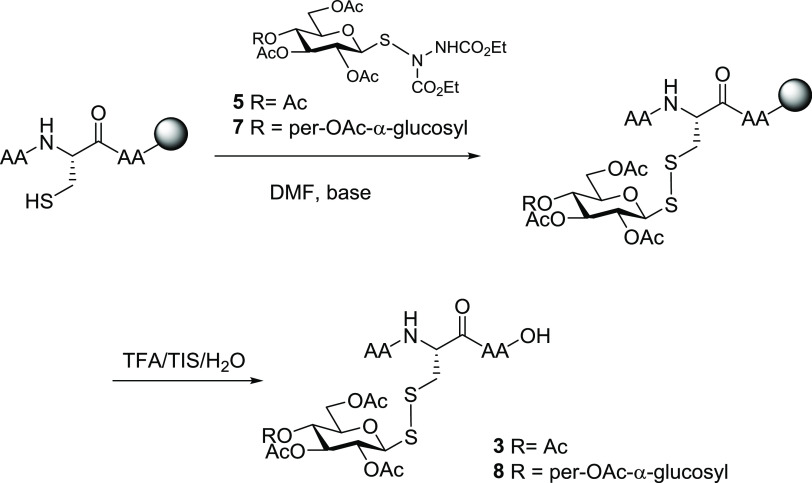
Solid-Phase Disulfide-Linked-Glycosylation of Peptides

**Scheme 4:**
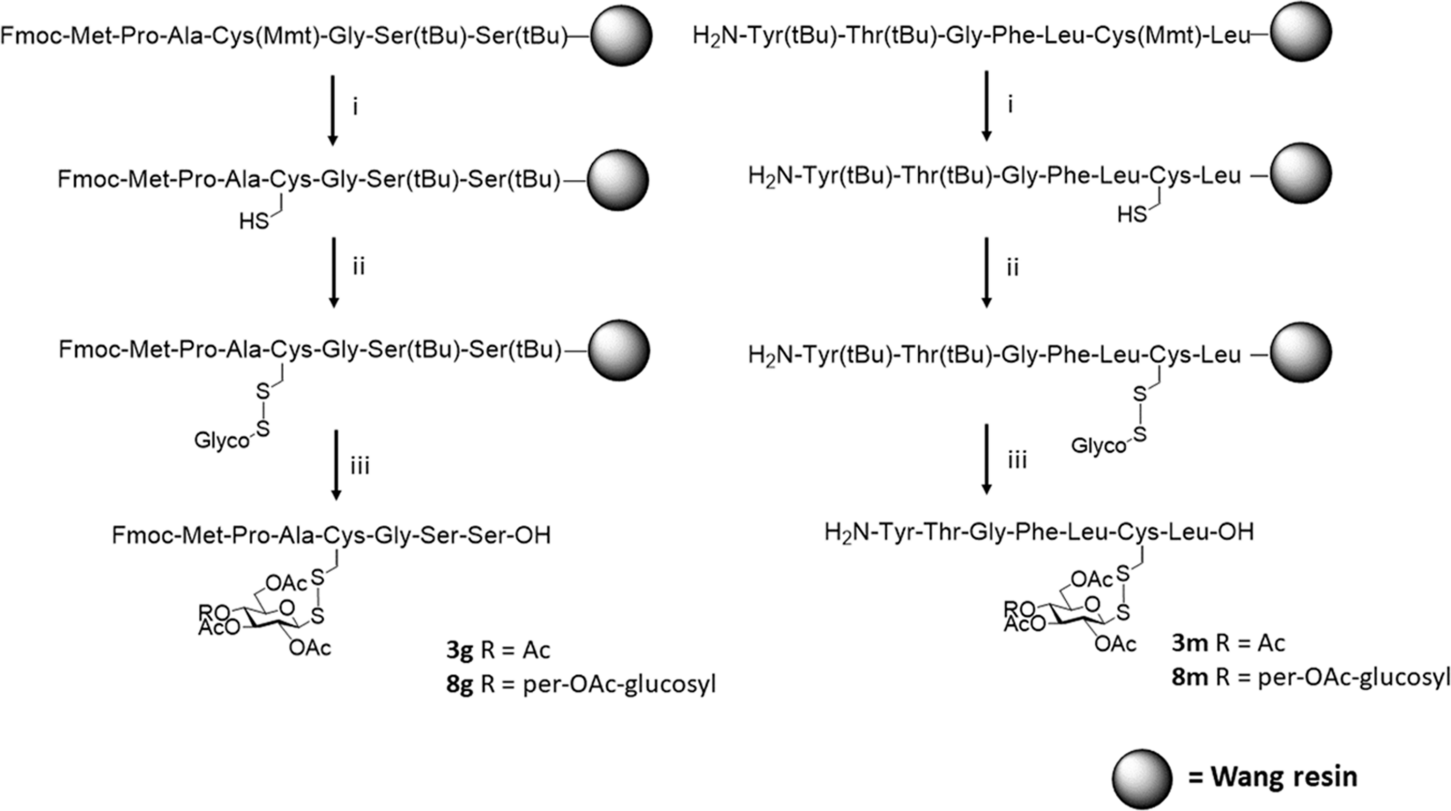
Solid-Phase Glycosylation of Fmoc-Met-Pro-Ala-Cys-Gly-Ser-Ser-OH (Yielding Compounds **3g** and **8g**) and H_2_N-Tyr-Thr-Gly-Phe-Leu-Cys-Leu-OH (Yielding Compounds **3m** and **8m**) Synthetic conditions: (i) CH_2_Cl_2_/TFA/TIS 95:2:3, 3 cycles of 10 min; (ii) **5** or **7** (2.5 equiv), *N*,*N*-diisopropylethylamine (DIPEA) (1 equiv), dimethylformamide (DMF) 2 cycles × 30 min; (iii) TFA/TIS/water 95:2.5:2.5, 4 h.

Next, we set out to investigate the feasibility of this approach to glycosylate resin-bound substrates (Scheme 3), since the ability to complete these as part of conventional solid-phase peptide synthesis protocols would be a significant advantage. The peptides were synthesized using Mmt-protected Cys residues. Selection of this orthogonal acid-labile protective group allows for selective cleavage while the peptide remains bound to the resin. We selected two model peptides (Fmoc-Met-Pro-Ala-Cys(Mmt)-Gly-Ser(*t*Bu)-Ser(*t*Bu)-OH and H_2_N-Tyr(*t*Bu)-Thr(*t*Bu)-Gly-Phe-Leu-Cys(Mmt)-Leu-OH), each loaded onto a Wang resin to react with both protected sulfenylhydrazine derivatives, glucose (**5**) and maltose (**7**) (Schemes 3 and 4). Selective deprotection of the Mmt group to expose the thiol of the Cys residue while on the resin was achieved using very mild acidic conditions (TFA/CH_2_Cl_2_/TIS 2:95:3), and complete glycosylation with the electrophilic thiol-specific glycosyl (**5** or **7**) (2.5 equiv) was accomplished using 2 repetitions of a 30 min reaction. Acidic hydrolysis of the peptide from the resin, with simultaneous deprotection of the amino acid side-chain protective groups, yielded the desired disulfide-linked glycopeptides, fully acetylated (Scheme 4). Both activated mono- and disaccharides proved to be equally effective in glycosylating peptides on solid phase. It was important to establish that the newly formed glycosyl disulfide linkages were stable to standard cleavage conditions required to hydrolyze the peptide from the resin (*i.e.*, strongly acidic—TFA/TIS/water 95:2.5:2.5). This proved to be so, with disulfide-linked glycopeptides **3g**, **3m**, **8g**, and **8m** (Scheme 4) being efficiently produced. The compatibility of this method of glycosylation with conventional solid-phase peptide synthesis will be extremely useful for site-specific mono-glycosylation of peptides containing two or more Cys residues.

The use of glycosulfenyl reagents **5** and **7** required subsequent sugar protective group hydrolysis, which was easily achieved by treating the per-*O*-acetylated glycopeptides with dilute basic conditions, followed by neutralization with an acidic resin. The disulfide bond was found to be stable under these conditions. In a search for an even simpler procedure to avoid the need for a postmodification step, we decided to investigate the possibility of preparing the glycosyl reagent in an unprotected form. In this case, more polar and protic solvents were necessary, as unprotected thioglucose **15** was not sufficiently soluble in THF.

**Figure 1:**
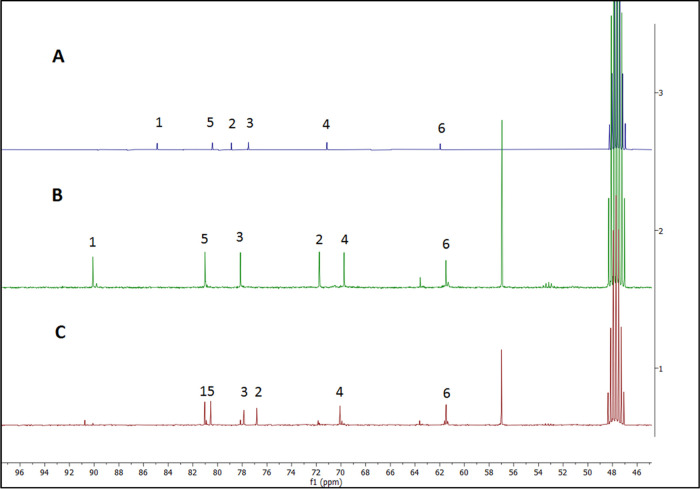
^13^C NMR spectra recorded in CD_3_OD. (A) ^13^C NMR spectrum of d-thioglucose sodium salt; (B) ^13^C NMR spectrum of reaction of d-thioglucose sodium salt with dilute deuterated methanolic DEAD (2.0 equiv) (instant reaction); (C) ^13^C NMR spectrum after addition of PhSH (2.0 equiv) (1 h).

**Scheme 5:**
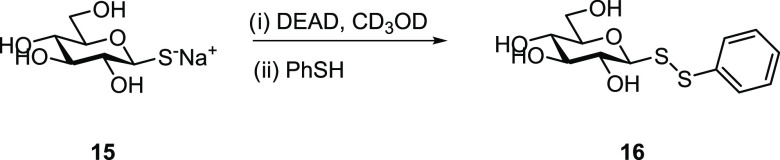
Schematic Representation of Synthesis of Unprotected Glycosyl Disulfide **16**

The absence of chromophores in both the relatively polar thioglucose and DEAD reagents made following the reaction for this alternative approach more challenging either by thin-layer chromatography (TLC) or LC/MS. The reaction was thus monitored by NMR analysis. To enable this, a deuterated methanolic solution of thioglucose **15** (1 equiv) was added dropwise to a solution of DEAD (2 equiv) in CD_3_OD at room temperature (Scheme 5). At the end of the addition period, the signal corresponding to C1 of thioglucose (Figure 1A,B) had shifted completely to low field, which indicated that a chemical modification occurred at that carbon (*i.e.*, at the thiol group). The reaction of unprotected thioglucose with azo reagent was instant, and similarly to the synthesis of acetate-protected reagents **5** and **7**, we assumed the product to be the unprotected glucosulfenyl hydrazine. To rule out the potential reaction with unprotected hydroxyl groups, since azodicarboxylate reagents can be used to oxidize alcohols to ketones in the presence of a Lewis acid,^36^ we also reacted unprotected glucose with DEAD under the same conditions. No change in the ^13^C NMR spectrum was observed when compared to the ^13^C NMR spectrum of glucose. This finding confirms that the reaction of thioglucose **15** with azo reagent occurred at the thiol group.

To further investigate the reactivity of the resulting product with thiols in protic solvents, thiophenol (2 equiv) was added to the same deuterated methanolic solution (Scheme 5). After 1 h, NMR analysis indicated that the C1 signal had shifted almost completely to upfield (Figure 1B,C), confirming that the product from the reaction of **15** with azodicarboxylate is equally reactive with thiols to produce directly unprotected glycosyl disulfides.

**Scheme 6:**
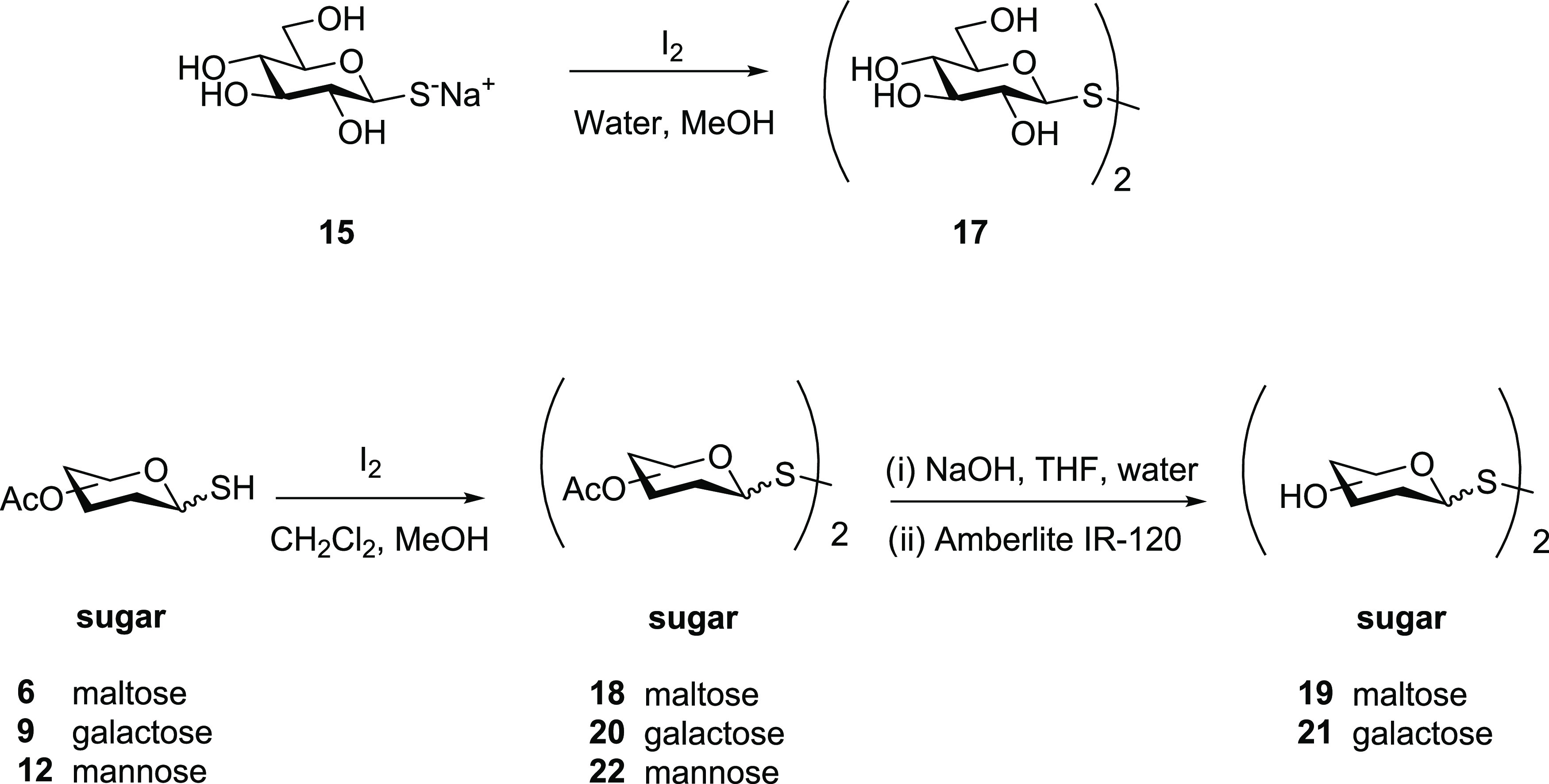
Synthesis of Symmetrical Dithiosaccharides

Despite these initially encouraging data, further NMR analysis of the pure product from the reaction of thioglucose **15** with DEAD (achieved by precipitation from diethyl ether) indicated an absence of the expected proton signals for the ethyl group, as well as the carbon signals for the ethyl ester group, typical of a glucosulfenyl hydrazine dicarboxylate diethyl intermediate. Instead, NMR analysis of the pure product from the reaction of **15** with DEAD indicated the presence of the unprotected symmetrical glucosyl disulfide, which was apparently formed instantly as the sole product despite the slow addition of the diluted methanolic solution of sugar to a solution of excess DEAD (2 equiv). To confirm this finding, unprotected symmetrical glucosyl disulfide **17** was intentionally prepared using an alternative route, specifically by oxidation of the unprotected thiosugar with iodine (Scheme 6).^37^ Comparison of the NMR spectra (^1^H/^13^C) revealed the same compound as previously obtained with the reaction of **15** with DEAD. This was a surprising observation since these were the same stoichiometric conditions as employed previously for the formation of protected glucosulfenyl hydrazine **5**.

To establish whether the formation of the symmetrical glycosyl disulfide is the result of using a protic solvent (the reaction of the protected 1-thioglucose was performed in THF) or if it was a structural effect (due to the acetate protecting groups or their absence), we reacted per-*O*-acetylated thioglucose **1** with DEAD (2 equiv) in methanol under the same conditions as for unprotected thioglucose **15**. In this case, the glucosulfenyl hydrazine **5** was again the main product, albeit with the residual formation of the per-*O*-acetylated symmetrical glucosyl disulfide. We speculated that potential hydrogen bonding between the thiol group with the neighboring acetate in compound **1**, which is absent in thioglucose **15**, might stabilize the thiol, and thus decrease its reactivity toward the electrophilic sulfur of intermediate **5**. Also, and perhaps more significantly, the sulfur in compound **15** is in the thiolate form, which is a much better nucleophile.

Despite the formation of the symmetrical glucosyl disulfide as the sole product when unprotected thioglucose **15** was reacted with azo reagent, synthesis of the desired unprotected phenyl glycosyl disulfide **16** was nevertheless achieved efficiently through a subsequent thiol–disulfide exchange reaction, instead of nucleophilic attack to the electrophilic unprotected glucosyl sulfenyl intermediate.

**Figure 2:**
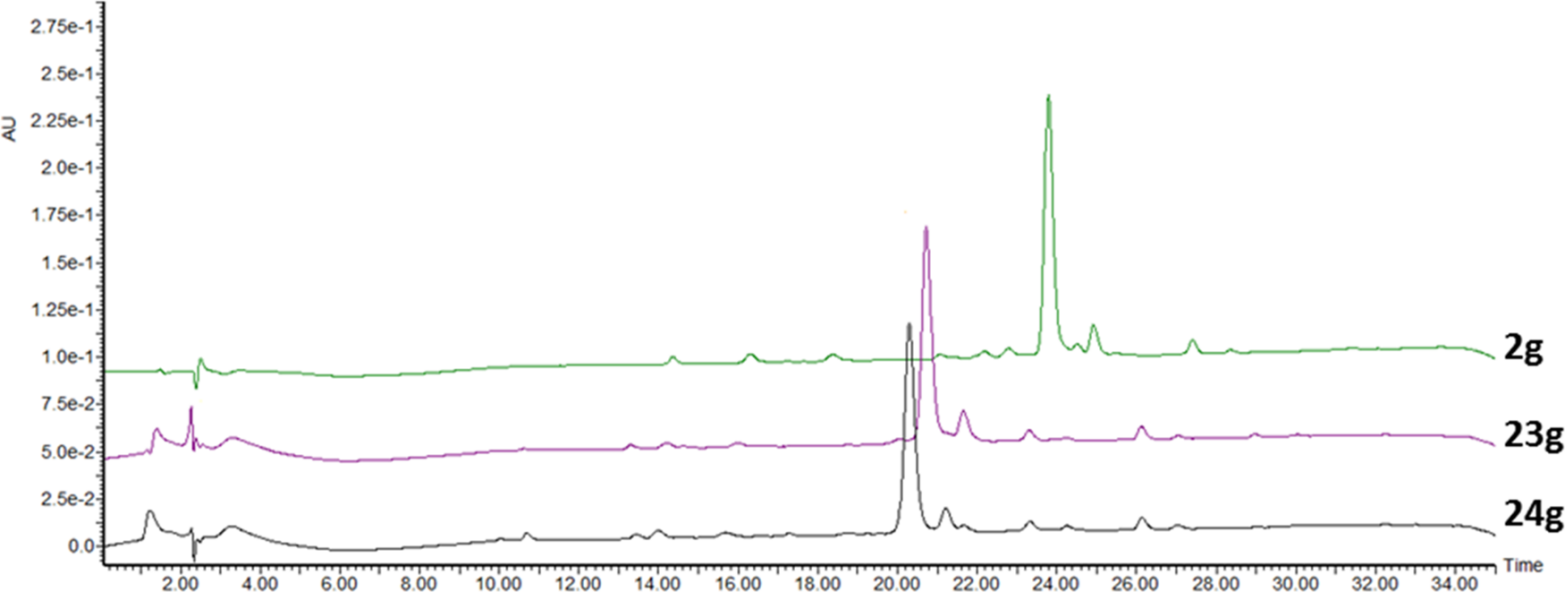
High-performance liquid chromatography (HPLC) chromatogram (λ = 254 nm) of peptide **2g** (green), and crude reaction mixtures after reaction with symmetrical glycosyl disulfide **17** (yielding compound **23g**, purple), and **19** (yielding compound **24g**, black), respectively.

Following these findings, the reactivity of symmetrical disulfide **17** with model peptides **2c**, **2g**, **2h**, and **2i** was explored (Table 2, entries 1–4). As shown in Figure 2 for peptide **2g**, symmetrical dithiodiglucoside (**17**) was equally susceptible to thiol–disulfide exchange by the cysteine—the starting peptide was fully converted into disulfide-linked glucopeptide **23g** within 30 min. Similarly, unprotected symmetrical dithiodimaltopyranoside (**19**) and dithiogalactopyranoside (**21**), which were prepared *via* acetate hydrolysis of the respective per-*O*-acetylated precursors **18** and **20** (Scheme 6), were found to be suitable for the glycosylation of peptides (Table 2, entries 5–12). Furthermore, we prepared the fully per-*O*-acetylated symmetrical dithiodiglucoside and reacted it with peptide **2c**. This was shown to be similarly efficient to glycosylate peptides *via* a disulfide bond (data not shown). While glycosylation of peptide **2g** with both mannosulfenyl transfer reagents **13** and **22** was equally efficient (Table 1, entry 23; Table 2, entry 14), for peptides **2c** and **2i**, the results were clearly superior when symmetrical dithiodimannopyranoside **22** was used, with excellent conversion to the desired product within 30 min, and insignificant formation of the cystine peptide dimer (Table 2, entries 13 and 15). Previously, we reported the synthesis of glycosyl disulfides *via* the exchange of thiosugar with symmetrical alkyl and aryl disulfides;^25^ however, to the best of our knowledge, this is the first report of a thiol–disulfide exchange reaction that utilizes symmetrical glycosyl (mono- and disaccharide) disulfides for the synthesis of disulfide-linked glycopeptides. Moreover, the facile synthesis of the unprotected symmetrical glycosyl disulfides and their stability for long-term storage prove to be superior to glycosylate peptides than the use of other glycosulfenyl transfer agents such as the glycosulfenic acid or glycosulfenamides.^32,33^ In addition to a more elaborate synthesis, glycosulfenic acids are unstable and were only shown to glycosylate fully protected cysteine,^32^ while glycosulfenamide was only used as fully per-*O*-acetylated.^33^

**Table 2:**
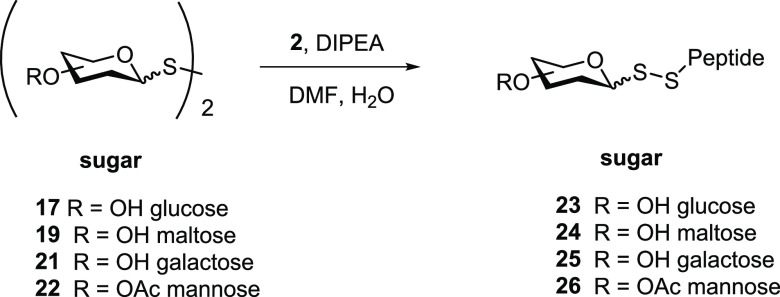
Synthesis of Fully Deprotected Disulfide-Linked Glycopeptides *via* Disulfide Exchange Reaction

entry	glyco	peptide	product (%)	ES MS found (calcd)
**1**	**17**	**2c** (Fmoc-βAla-Cys-Lys-Leu-OH)	**23c** (>90%)	850.0 [M + H]^+^ (849.00)
**2**	**17**	**2g** (Fmoc-Met-Pro-Ala-Cys-Gly-Ser-Ser-OH)	**23g** (>90%)	1068.5 [M + H]^+^ (1067.18)
**3**	**17**	**2h** (Fmoc-βAla-Gly-Cys-Ala-Cit-Leu-His-Leu-OH)	**23h** (>90%)	629.4 [M + 2H]^2+^ (1256.51)
**4**	**17**	**2i** (Fmoc-βAla-Arg-Cys-Gly-Asn-Leu-OH)	**23i** (>90%)	1049.6 [M + H]^+^ (1048.39)
**5**	**19**	**2c** (Fmoc-βAla-Cys-Lys-Leu-OH)	**24c** (>90%)	1012.5 [M + H]^+^ (1011.38)
**6**	**19**	**2g** (Fmoc-Met-Pro-Ala-Cys-Gly-Ser-Ser-OH)	**24g** (>90%)	637.7 [M + 2Na]^2+^ (1229.38)
**7**	**19**	**2h** (Fmoc-βAla-Gly-Cys-Ala-Cit-Leu-His-Leu-OH)	**24h** (>90%)	710.6 [M + 2H]^2+^ (1418.57)
**8**	**19**	**2i** (Fmoc-βAla-Arg-Cys-Gly-Asn-Leu-OH)	**24i** (>90%)	617.3 [M + H + Na]^2+^ (1210.45)
**9**	**21**	**2c** (Fmoc-βAla-Cys-Lys-Leu-OH)	**25c** (>90%)	850.3 [M + H]^+^ (849.00)
**10**	**21**	**2g** (Fmoc-Met-Pro-Ala-Cys-Gly-Ser-Ser-OH)	**25g** (>90%)	1068.2 [M + H]^+^ (1067.18)
**11**	**21**	**2h** (Fmoc-βAla-Gly-Cys-Ala-Cit-Leu-His-Leu-OH)	**25h** (>90%)	629.5 [M + 2H]^2+^ (1256.51)
**12**	**21**	**2i** (Fmoc-βAla-Arg-Cys-Gly-Asn-Leu-OH)	**25i** (>90%)	1049.3 [M + H]^+^ (1048.39)
**13**	**22**	**2c** (Fmoc-βAla-Cys-Lys-Leu-OH)	**26c** (>90%)	1018.3 [M + H]^+^ (1017.37)
**14**	**22**	**2g** (Fmoc-Met-Pro-Ala-Cys-Gly-Ser-Ser-OH)	**26g** (>90%)	619.7 [M + 2H]^2+^ (1236.34)
**15**	**22**	**2i** (Fmoc-βAla-Arg-Cys-Gly-Asn-Leu-OH)	**26i** (∼80%)	609.3 [M + 2H]^2+^ (1217.32)

## Conclusions

We have presented efficient new routes to the synthesis of disulfide-linked glycopeptides that allow introduction of both protected and fully deprotected carbohydrates and conditions that are compatible with both solution-phase chemistry and solid-phase peptide synthesis. The use of azo compounds for the synthesis of disulfides is well established, and herein, we have expanded their scope, through a glycosyl–DEAD intermediate, to the synthesis of disulfide-linked glycopeptides, both with mono- and disaccharides. For this application, it was found to be advantageous to isolate the protected glycosyl sulfenylhydrazine reagent, which can be synthesized in a rapid and facile manner—this can be conveniently stored as a reactive building block ready for future use. This then provides instant access to the desired glycopeptides, with full site-specific control within the peptide to cysteine residues through a disulfide bond. We have shown this methodology to be a highly versatile strategy that does not require prior amino acid side-chain protection within the peptide, a significant advantage since this is a common drawback of existing methods. In addition, exploitation of the glycosyl–DEAD reagents allows access to disulfide-linked glycopeptides either in solution phase, or with peptides immobilized on a solid support, as is typically the case in solid-phase peptide synthesis. To the best of our knowledge, this is the first report of methodology for glycosylation of peptides linked through a disulfide bond at the anomeric carbon suitable for solid-phase peptide synthesis.

Further application of the methodology to unprotected sugars demonstrated that in this case, symmetrical dithiosaccharides were instead responsible for glycosylation of the peptides *via* a thiol–disulfide reaction. The simplicity of utilizing glycosyl disulfide exchange to prepare either protected or unprotected glycosyl disulfides, including disulfide-linked glycopeptides, is very attractive. Moreover, this thiol–disulfide exchange offers the advantage to glycosylate peptides with fully unprotected glycosyl reagents, with no need for a postmodification deprotection step. Equally importantly, the compatibility of this methodology with aqueous conditions additionally opens up the possibility for use in the glycosylation of proteins.

Given the importance of glycosylated peptides in structural glycobiology, pharmacology, and therapeutics, the methodology outlined provides easy access to disulfide-linked glycopeptides as molecules with multiple biological applications.

## Experimental Section

### General Information

NMR spectra were recorded on a Bruker AMX 400 NMR spectrometer and are reported in parts per million (ppm) on the δ scale relative to residual CDCl_3_ (δ 7.25 or δ 77.0), CD_3_OD (δ 3.31 or δ 49.00), or D_2_O (δ 4.79). Spectral assignments were accomplished using two-dimensional (2D) COSY and HSQC experiments. The progress of the reactions was monitored by analytical thin-layer chromatography (Merck, TLC 60 F_254_ plates) and/or by LC/MS using Waters Alliance 2695 Separations Module, Waters 996 PDA Detector, and Waters Micromass ZQ Mass Detector. TLC plates were visualized first with UV (254 nm) and then illuminated by sulfuric acid solution (10% sulfuric acid in ethanol), followed by heating. High-resolution mass spectra were recorded using Thermo scientific, LTQ Orbitrap no. 01289B. Column chromatography was performed using silica gel (230–400 mesh). The solvent compositions for all separations are on a volume/volume (v/v) basis. All solvents were of reagent grade. 1-Thio-β-d-glucose tetraacetate, diisopropyl azodicarboxylate, d-(+)-maltose, and 1-thio-β-d-glucose sodium salt were purchase from Aldrich. Diethyl azodicarboxylate solution 40 wt % in toluene was purchased from Carbosynth. 1-Thio-β-d-maltose heptaacetate was synthesized from d-(+)-maltose in the following steps: (1) per-*O*-acetylation using Ac_2_O, 4-dimethylaminopyridine (DMAP), pyridine, (2) bromination at the anomeric carbon using 33% HBr/CH_3_COOH, and (3) reaction of the brominated with thiourea followed by basic hydrolysis.^38,39^ Peptides were synthesized using standard Fmoc solid-phase peptide synthesis.^40^ Fmoc-Leu-Wang resin was used for the synthesis of peptides **2a**, **2b**, **2c**, **2d**, **2f**, **2h**, **2i**, and **2j**; H-Cys(Trt)-2-chlorotrityl resin was used for the synthesis of peptide **2e**; and Fmoc-Ser(*t*Bu)-Wang resin was used for the synthesis of peptide **2g**. Peptide **2l** was synthesized after loading of Fmoc-Gly-OH onto Rink Amide MHBA. All amino acids and resins were purchased from Novabiochem. LC/MS details: column: Hichrom RPB microbore column (150 × 2.1 mm^2^); mobile phase A: 90% water, 10% MeOH, 0.1% formic acid; mobile phase B: 90% MeOH, 10% water, 0.1% formic acid; flow: 0.25 mL/min; gradient:

time (min)mobile phase
A (%)mobile
phase B (%)09555505028010030010035955

#### Synthesis of β-d-Glucopyranose, 4-*O*-(2,3,4,6-Tetra-*O*-acetyl-α-d-glucopyranosyl)-1-thio-, 2,3,6-Triacetate (**6**)

To a solution of d-(+)-maltose (10.0 g, 29.2 mmol) in pyridine (55 mL) and acetic anhydride (35 mL) was added DMAP (340 mg, 2.8 mmol), and the reaction mixture was stirred overnight at room temperature. This was then diluted with ethyl acetate (150 mL) and extracted with 1 M HCl (5 × 100 mL), NH_4_Cl (100 mL), and brine (100 mL). The organic phase was dried over MgSO_4_ and filtered, and the filtrate was evaporated under vacuum to afford β-d-maltose octaacetate (quantitative). Then, β-d-maltose octacetate (5.13 g, 7.5 mmol) was dissolved in CH_2_Cl_2_ (5 mL) and stirred with 48% HBr/acetic acid (15.5 mL). After 30 min, the reaction mixture was diluted with CH_2_Cl_2_ (150 mL) and was extracted with sat NaHCO_3_ (3 × 100 mL). The organic phase was dried over MgSO_4_, and after evaporation of the filtrate, the resulting solid was refluxed with acetone (12 mL) and thiourea (2.56 g, 33.6 mmol) for 2 h. The solvent was evaporated, and the residue was redissolved in dichloroethane (21 mL) and refluxed with water (25 mL) and sodium metabisulfite (1.5 g). After 20 min, the reaction mixture was extracted with DCM (100 mL). The organic phase was dried over MgSO_4_ and filtered, and the filtrate was evaporated under vacuum. Column chromatography on silica gel (hexane/ethyl acetate 1:1 → 1:1.5) afforded compound **7** as white solid (2.6 g, 53%) - ^1^H NMR (CDCl_3_, 400 MHz) δ 5.40 (d, 1H, *J*_1′,2′_ = 4.0 Hz, H1′), 5.35 (dd, 1H, *J*_3′,4′_ = 9.4 Hz, *J*_2′,3′_ = 10.5 Hz, H3′), 5.24 (dd, 1H, *J*_2,3_ = 9.6 Hz, *J*_3,4_ = 9.6 Hz, H3), 5.04 (dd, 1H, *J*_3′,4′_ = 9.4 Hz, *J*_4′,5′_ = 9.4 Hz, H4′), 4.85 (dd, 1H, *J*_1′2′_ = 4.0 Hz, *J*_2′,3′_ = 10.5 Hz, H2′), 4.80 (dd, 1H, *J*_1,2_ = 9.6 Hz, *J*_2,3_ = 9.6 Hz, H2), 4.58 (dd, *J*_1,2_ = 9.6 Hz, *J*_1,SH_ = 9.6 Hz, H1), 4.45 (dd, 1H, *J*_5,6b_ = 2.6 Hz, *J*_6a,6b_ = 12.3 Hz, H6a), 4.19–4.25 (m, 2H, H6b, H6a′), 4.04 (dd, 1H, *J*_5′,6a′_ = 2.4 Hz, *J*_6a′,6b′_ = 12.7 Hz, H6b′), 4.00 (dd, 1H, *J*_4,5_ = 8.8 Hz, *J*_3,4_ = 9.6 Hz, H4), 3.92–3.96 (m, 1H, H5′), 3.70–3.75 (m, 1H, H5), 2.27 (d, 1H, *J*_1,SH_ = 9.6 Hz, SH), 2.17, 2.12, 2.07, 2.06, 2.04, 2.03, 2.02 (7s, 21H, 7OAc); ^13^C{^1^H} NMR (CDCl_3_, 100 MHz) δ 95.6, 78.2, 76.5, 76.0, 74.3, 72.6, 69.9, 69.3, 68.6, 67.9, 63.0, 61.4, 20.9, 20.8, 20.7 (2C), 20.6, 20.5 (2C); High-resolution mass spectrometry (HRMS) (ESI^–^) [M – H]^−^ calcd for C_26_H_35_O_16_S 651.1595; found 651.1598.

#### General Procedure for the Synthesis of Glycosulfenyl Hydrazines **5**, **7**, **10**, and **13**

A solution of saccharide (1 mmol) in THF (15 mL) was added dropwise to a solution of DEAD (2 mmol, 0.75 mL, 40% sol in toluene) in THF (15 mL) at RT. At the end of the addition, the solvent was evaporated, and the residue was purified by column chromatography on silica gel (hexane/EtOAc 1.5:1 → 1:1 → 1:2) to produce desired compounds.

#### 1,2-Hydrazinedicarboxylic Acid, 1-(2′,3′,4′,6′-Tetra-*O*-acetyl-1′-thio-β-d-glucopyranosyl)-1,2-diethyl Ester (**5**)

η = 84% (450 mg); white solid; ^1^H NMR (CDCl_3_, 400 MHz) δ 7.24 (bs, 1H, NH), 5.27 (dd, 1H, *J*_2,3_ = 9.6 Hz, *J*_3,4_ = 9.6 Hz, H3), 5.12 (dd, 1H, *J*_3,4_ = 9.6 Hz, *J*_4,5_ = 9.6 Hz, H4), 5.01 (dd, 1H, *J*_1,2_ = 9.6 Hz, *J*_2,3_ = 9.6 Hz, H2), 4.93 (d, 1H, *J*_1,2_ = 9.6 Hz, H1), 4.29 (m, 6H, 2CH_2_, H6a, H6b), 3.77 (m, 1H, H_5_), 2.12, 2.09, 2.05, 2.02 (4s, 12H, 4OAc), 1.31 (t, 3H, CH_3_, *J* = 7.2 Hz), 1.29 (t, 3H, CH_3_, *J* = 7.2 Hz); ^13^C{^1^H} NMR (CDCl_3_, 100 MHz) δ 170.7, 170.1, 169.6, 169.3, 156.1, 155.5, 88.2, 75.9, 73.6, 68.4, 68.0, 64.5, 62.3, 61.8, 20.6, 20.6, 20.5, 20.5, 14.4, 14.2; HRMS (ESI) [M + H]^+^ calcd for C_20_H_31_O_13_N_2_S 539.1546; found 539.1620.

#### 1,2-Hydrazinedicarboxylic Acid, 1-[(2′,3′,6′-Tri-*O*-acetyl-4-*O*-(2′,3′,4′,6′-tetra-*O*-acetyl-α-d-glucopyranosyl)-1′-thio-β-d-glucopyranosyl)]-1,2-diethyl Ester (**7**)

η = 80% (660 mg); white solid; ^1^H NMR (CDCl_3_, 400 MHz) δ 5.35 (d, 1H, *J*_1′,2′_ = 4.0 Hz, H1′), 5.34 (dd, 1H, *J*_2′3′_ = 10.4 Hz, *J*_3′4′_ = 9.6 Hz, H3′), 5.29 (m, 1H, H2), 5.04 (dd, 1H, H4′, *J*_3′,4′_ = *J*_4′,5′_ = 9.6 Hz, H4′), 4.84 (m, 2H, H2′, H1), 4.59 (d, 1H, *J*_6a,6b_ = 9.2 Hz, H6a), 4.16–4.27 (m, 6H, 2CH_2_, H6b, H6′a), 3.91–4.06 (m, 3H, H3, H4, H6′b), 3.73 (m, 1H, H5), 2.14, 2.09, 2.03, 2.01, 1.99, 1.99 (6s, 21H, 7OAc), 1.26 (m, 6H, 2CH_3_); ^13^C{^1^H} NMR (CDCl_3_, 100 MHz) δ 170.7, 170.5, 170.1, 169.9, 169.8, 169.4, 156.1, 155.5, 95.6, 77.2, 76.4, 76.2, 72.1, 70.0, 69.2, 69.2, 68.5, 67.9, 64.5, 62.4, 62.3, 61.4, 21.0, 20.9, 20.8, 20.6, 20.6, 20.5, 14.4, 14.2; HRMS (ESI) [M + Na]^+^ calcd for C_32_H_46_O_21_N_2_SNa 849.2211; found 849.2326.

#### 1,2-Hydrazinedicarboxylic Acid, 1-(2′,3′,4′,6′-Tetra-*O*-acetyl-1′-thio-β-d-galactopyranosyl)-1,2-diethyl Ester (**10**)

η = 90% (1.25 g); white solid; ^1^H NMR (CDCl_3_, 400 MHz) δ 7.10 (bs, 1H, NH), 5.44 (dd, 1H, *J*_4,5_ = 1.2 Hz, *J*_3,4_ = 3.2 Hz, H4), 5.12 (m, 2H, H2 and H3), 4.99 (d, 1H, *J*_1,2_ = 8.0 Hz, H1), 4.17 (m, 6H, 2CH_2_, H6a, H6b), 3.96 (m, 1H, H5), 2.15, 2.08, 2.05, 1.98 (4s, 12H, 4OAc), 1.30 (t, 3H, CH_3_, *J* = 7.2 Hz), 1.28 (t, 3H, CH_3_, *J* = 7.2 Hz); ^13^C{^1^H} NMR (CDCl_3_, 100 MHz) δ 170.4, 170.1, 169.9, 156.0, 155.5, 74.7, 71.6, 67.1, 65.5, 64.4, 62.3, 20.6, 20.6, 29.5, 14.4, 14.3; HRMS (ESI) [M + H]^+^ calcd for C_20_H_31_O_13_N_2_S 539.1546; found 539.1579.

#### 1,2-Hydrazinedicarboxylic Acid, 1-(2′,3′,4′,6′-Tetra-*O*-acetyl-1′-thio-α-d-mannopyranosyl)-1,2-diethyl Ester (**13**)

η = 45% (670 mg); oil; ^1^H NMR (CDCl_3_, 400 MHz) δ 7.34 (bs, 1H, NH), 5.66 (s, 1H, H1), 5.39 (d, 1H, *J*_2,3_ = 3.2 Hz, H2), 5.30 (dd, 1H, *J*_3,4_ = 9.6 Hz, *J*_4,5_ = 10.2 Hz), 5.02 (dd, 1H, *J*_2,3_ = 3.2 Hz, *J*_3,4_ = 9.6 Hz, H3), 4.51 (m, 1H, H5), 4.17 (m, 6H, 2CH_2_, H6a, H6b), 2.16, 2.13, 2.06, 1.98 (4s, 12H, 4OAc), 1.30 (t, 3H, CH_3_, *J* = 7.2 Hz), 1.28 (t, 3H, CH_3_, *J* = 7.2 Hz); ^13^C{^1^H} NMR (CDCl_3_, 100 MHz) δ 170.4, 169.8, 169.6, 155.9, 155.2, 70.3, 69.6, 67.5, 66.0, 64.7, 62.5, 20.8, 20.7, 20.6, 20.5, 14.4, 14.3,14.1; HRMS (ESI) [M + H]^+^ calcd for C_20_H_31_O_13_N_2_S 539.1546; found 539.1583.

#### Synthesis of 1,1′-Dithio-β-diglucopyranoside (**17**)

To a solution of unprotected thioglucose sodium salt (325 mg, 1.5 mmol) in MeOH (5 mL) and water (1 mL) was added iodine (375 mg, 1.5 mmol). After 30 min, diethyl ether was added, and the solid was filtered. The precipitate was redissolved in methanol and reprecipitated from diethyl ether and washed thoroughly with dichloromethane to provide **17** (285 mg, 98%) as a white solid. No further purification was carried out—^1^H NMR (CD_3_OD, 400 MHz) δ 4.43 (d, 1H, *J*_1,2_ = 9.6 Hz, H1), 3.89 (dd, 1H, *J*_5,6b_ = 1.2 Hz, *J*_6a,6b_ = 11.8 Hz, H6a), 3.69 (dd, 1H, *J*_5,6a_ = 5.6 Hz, *J*_6a,6b_ = 11.8 Hz, H6b), 3.51 (dd, 1H, *J*_2,3_ = 8.6 Hz, *J*_1,2_ = 9.6 Hz, H2), 3.40 (dd, 1H, *J*_3,4_ = 9.0 Hz, *J*_2,3_ = 8.6 Hz, H3), 3.29–3.36 (m, 2H, H4 and H5); ^13^C{^1^H} NMR (CD_3_OD, 100 MHz) δ 90.0, 81.0, 78.1, 71.7, 69.7, 61.4; HRMS (ESI) [M + H]^+^ calcd for C_12_H_23_O_10_S_2_ 391.0732; found 391.0728.

### General Procedure for the Synthesis of Peracetylated 1,1′-Dithio-glycosides **18**, **20**, and **22**

To a solution of saccharide (1 mmol) in CH_2_Cl_2_ (4 mL) and methanol (2 mL) was added iodine (1.1 mmol). After 30 min, CH_2_Cl_2_ (50 mL) was added and was extracted with sat. solution of sodium metabisulfite (50 mL) and water (50 mL). The organic phase was dried over MgSO_4_ and filtered, and the filtrate was concentrated under vacuum. Column chromatography on silica gel (hexane/ethyl acetate 1:1) was performed to provide desired compounds.

#### Bis[2,3,4,6-tetra-*O*-acetyl-α-d-glucopyranosyl-(1 → 4)-(2,3,6-tri-*O*-acetyl-1-deoxy-1-thio-β-d-glycopyranosyl)]-1,1′-disulfide (**18**)

η = 90% (340 mg); white solid; ^1^H NMR (CDCl_3_, 400 MHz) δ 5.44 (d, 1H, *J*_1′,2′_ = 4.0 Hz, H1′), 5.36 (dd, 1H, *J*_3′,4′_ = 9.6 Hz, *J*_2′,3′_ = 10.5 Hz, H3′), 5.30 (dd, 1H, *J*_2,3_ = 9.6 Hz, *J*_3,4_ = 9.4 Hz, H3), 5.07 (dd, 1H, *J*_3′,4′_ = 9.6 Hz, *J*_4′,5′_ = 9.6 Hz, H4′), 4.96 (dd, 1H, *J*_2,3_ = 9.6 Hz, *J*_1,2_ = 9.6 Hz, H2), 4.86 (dd, 1H, *J*_1′,2′_ = 4.0 Hz, *J*_2′,3′_ = 10.5 Hz, H2′), 4.67 (dd, 1H, *J*_5,6b_ = 2.4 Hz, *J*_6a,6b_ = 12.5 Hz, H6a), 4.61 (d, 1H, *J*_1,2_ = 9.6 Hz), 4.21–4.28 (m, 2H, H6b, H6a′), 4.07 (dd, 1H, *J*_5′,6a′_ = 2.4 Hz, *J*_6a′,6b′_ = 12.6 Hz, H6b′), 4.02 (dd, 1H, *J*_4,5_ = 8.8 Hz, *J*_3,4_ = 9.4 Hz, H4), 3.94–3.98 (m, 1H, H5′), 3.75–3.79 (m, 1H, H5), 2.19, 2.10, 2.05, 2.03, 2.02, 2.01, 2.00 (7s, 21H, 7OAc); ^13^C{^1^H} NMR (CDCl_3_, 100 MHz) δ 170.5 (2C), 170.3, 170.1, 169.9, 169.4, 169.4, 95.6, 77.2, 76.5, 76.3, 72.1, 70.7, 70.0, 69.3, 68.5, 67.9, 62.3, 61.3, 20.9, 20.9, 20.7, 20.6, 20.6, 20.6, 20.5; HRMS (ESI) [M + Na]^+^ calcd for C_52_H_70_NaO_34_S_2_ 1325.3087; found 1325.3118.

#### Bis[2,3,6-tri-*O*-acetyl-1-deoxy-1-thio-β-d-galactopyranosyl]-1,1′-disulfide (**20**)

η = 54% (650 mg); white solid; ^1^H NMR (CDCl_3_, 400 MHz) δ 5.44 (dd, 1H, *J*_4,5_ = 1.0 Hz, *J*_3,4_ = 3.4 Hz, H4), 5.35 (dd, 1H, *J*_1,2_ = *J*_2,3_ = 9.9 Hz, H2), 5.08 (dd, 1H, *J*_3,4_ = 3.4 Hz, *J*_2,3_ = 9.9 Hz), 4.57 (d, 1H, *J*_1,2_ = 9.9 Hz, H1), 4.23 (dd, 1H, *J*_5,6b_ = 5.8 Hz, *J*_6a,6b_ = 11.2 Hz, H6a), 4.11 (dd, 1H, *J*_5,6a_ = 6.8 Hz, *J*_6a,6b_ = 11.2 Hz, H6b), 4.03 (ddd, 1H, *J*_4,5_ = 1.0 Hz, *J*_5,6b_ = 5.8 Hz, *J*_5,6a_ = 6.8 Hz, H5), 2.17, 2.09, 2.05, 1.98 (4s, 24H, 8OAc); ^13^C{^1^H} NMR (CDCl_3_, 100 MHz) δ 170.2, 170.1, 170.0, 169.3, 88.5, 74.8, 71.8, 67.6, 67.0, 60.8, 20.8, 20.6, 20.6, 20.5; HRMS (ESI) [M + Na]^+^ calcd for C_28_H_38_NaO_18_S_2_ 749.1397; found 749.1437.

#### Bis[2,3,6-tri-*O*-acetyl-1-deoxy-1-thio-α-d-mannopyranosyl]-1,1′-disulfide (**22**)

η = 53% (160 mg); white solid; ^1^H NMR (CDCl_3_, 400 MHz) δ 5.35 (dd, 2H, *J*_1,2_ = 1.7 Hz, *J*_2,3_ = 3.6 Hz, H2), 5.22 (d, 2H, *J*_1,2_ = 1.7 Hz, H1), 5.17 (dd, 2H, *J*_3,4_ = *J*_4,5_ = 9.6 Hz, H4), 5.13 (dd, 2H, *J*_2,3_ = 3.6 Hz, *J*_3,4_ = 9.6 Hz, H3), 4.21 (dd, 2H, *J*_5,6b_ = 5.6 Hz, *J*_6a,6b_ = 12.4 Hz, H6a), 4.10 (m, 2H, H5), 3.98 (dd, 2H, 1H, *J*_5,6a_ = 2.4 Hz, *J*_6a,6b_ = 12.4 Hz, H6b), 2.05, 1.99, 1.95, 1.89 (4s, 24H, 8OAc); ^13^C{^1^H} NMR (CDCl_3_, 100 MHz) δ 170.4, 169.6, 169.5, 169.4, 87.3, 70.8, 69.5, 68.7, 65.7, 61.8; HRMS (ESI) [M + Na]^+^ calcd for C_28_H_38_NaO_18_S_2_ 749.1397; found 749.1385.

### General Procedure for the Synthesis of 1,1′-Dithio-glycosides **19** and **21**

A solution of peracetylated 1,1′-dithio-glycoside (1 mmol) in THF (20 mL), water (80 mL), and 0.5 M NaOH (40 mL) was stirred at room temperature. After 3 h, Amberlite IR-120 was added until the solution was pH 7, after which it was filtered. The filtrate was then partially concentrated under vacuum, followed by lyophilisation to provide compounds **19** and **21** (quantitative yield) with no further purification.

#### 1,1′-Dithio-β-dimaltopyranoside (**19**)

Amorphous solid; ^1^H NMR (D_2_O, 400 MHz) δ 5.42 (d, 1H, *J*_1′,2′_ = 4.0 Hz, H1′), 4.61 (d, 1H, *J*_1,2_ = 9.6 Hz, H1), 4.01–4.05 (m, 1H), 3.56–3.87 (m, 10H), 3.42 (dd, 1H, *J* = 9.6 Hz, *J* = 9.6 Hz); ^13^C{^1^H} NMR (D_2_O, 100 MHz) δ 99.5, 89.1, 78.8, 77.4, 76.1, 72.8, 72.6, 71.6, 71.0, 69.2, 60.7, 60.4; HRMS (ESI) [M + H]^+^ calcd for C_24_H_43_O_20_S_2_ 715.1789; found 715.1813.

#### 1,1′-Dithio-β-digalactopyranoside (**21**)

Amorphous solid; ^1^H NMR (D_2_O, 400 MHz) δ 4.64 (d, 2H, *J*_1,2_ = 9.6 Hz), 4.06 (d, 2H, *J*_3,4_ = 4.0 Hz, H4), 3.92 (dd, 2H, *J*_1,2_ = *J*_2,3_ = 9.6 Hz), 3.77–3.88 (m, 8H, H3, H5, H6a, H6b); ^13^C{^1^H} NMR (D_2_O, 100 MHz) δ 89.9, 79.4, 73.8, 68.7, 68.6, 61.1; HRMS (ESI) [M – H]^−^ calcd for C_12_H_21_O_10_S_2_ 389.0576; found 389.0607.

### General Procedure for the Glycosylation of Peptides with Per-Acetylated Saccharides in Solution

To a solution of cysteine-containing peptide (0.1 mmol) in DMF (2 mL) was added glycosyl sulfenylhydrazine **5**, **7**, **10**, or **13** (0.5 mmol) and DIPEA (0.1 mmol), and the reaction was stirred at RT. After 30 min, diethyl ether was added, which led to the formation of a solid. Filtration of the precipitate afforded the desired disulfide-linked glycopeptides as a white solid in 90–95% purity as determined by LC/MS. No further purification was carried out.

### General Procedure for Deacetylation of Glycopeptides

Fully acetylated disulfide-linked glycopeptide (15 mg) was suspended in water (15 mL), and 0.5 M NaOH (0.5 mL) was added at RT. After 2 h, a clear solution was developed, Amberlite IR-120 was added until pH 7, filtered, and the aqueous solution was lyophilized to provide fully deprotected disulfide-linked glycopeptide in 90–95% purity.

### General Procedure for the Glycosylation of Peptides with Per-Acetylated Saccharides on Solid Phase

Dry resin (100 mg, 0.7 mmol, 0.7 mmol/g) was washed with CH_2_Cl_2_ (1 × 20 min). Deprotection of the MMt was achieved by treatment with CH_2_Cl_2_/TFA/TIS 95:2:3 (5 mL, 3 × 10 min), which was subsequently washed with CH_2_Cl_2_ (3 × 1 min) and DMF (3 × 1 min). The resin was treated with glycosyl sulfenylhydrazine **5** or **7** (1.75 mmol) and DIPEA (0.7 mmol) in DMF (1 mL) and agitated for 2 × 30 min. The resin was then washed successively with DMF (3 × 1 min), MeOH (3 × 1 min), and CH_2_Cl_2_/MeOH 1:1 (3 × 1 min), followed by drying under vacuum for 1 h. The dried resin was treated with TFA/TIS/H_2_O (95:2.5:2.5) for 4 h at RT. The resin was filtered, and the cleavage mixture was evaporated under vacuum, precipitated with Et_2_O, centrifuged, and the pellet was redissolved in methanol for analysis by LCMS.

### General Procedure for the Glycosylation of Peptides with Unprotected Symmetrical Dithiodisaccharides

To a solution of cysteine-containing peptide (0.1 mmol) in DMF (1.2 mL) and water (0.3 mL) was added dithiodisaccharides **17**, **19**, **21**, or **22** (0.5 mmol) and DIPEA (0.1 mmol), and the reaction was stirred at RT. After 30 min, methanol and diethyl ether were added, which led to the formation of a solid. Filtration of the precipitate afforded desired unprotected disulfide-linked glycopeptides as a white solid in 90–95% purity. No further purification was carried out.

## Supporting Information Available

NMR spectra for compounds; characterization of glycopeptides by LCMS (PDF)

## References

[ref1] Banting F. G.; Best C. H.; Collip J. B.; Campbell W. R.; Fletcher A. A. Pancreatic Extracts in the Treatment of Diabetes Mellitus. Can. Med. Assoc. J. 1922, 12, 141–146. 20314060 PMC1524425

[ref2] Lau J. L.; Dunn M. K. Therapeutic peptides: Historical perspectives, current development trends, and future directions. Bioorg. Med. Chem. 2018, 26, 2700–2707. 10.1016/j.bmc.2017.06.052. 28720325

[ref3] Fosgerau K.; Hoffmann T. Peptide therapeutics: current status and future directions. Drug Discovery Today 2015, 20, 122–128. 10.1016/j.drudis.2014.10.003. 25450771

[ref4] Usmani S. S.; Bedi G.; Samuel J. S.; Singh S.; Kalra S.; Kumar P.; Ahuja A. A.; Sharma M.; Gautam A.; Raghava G. P. S. THPdb: Database of FDA-approved peptide and protein therapeutics. PLoS One 2017, 12, e0181748 10.1371/journal.pone.0181748. 28759605 PMC5536290

[ref5] Mathur D.; Prakash S.; Anand P.; Kaur H.; Agrawal P.; Mehta A.; Kumar R.; Singh S.; Raghava G. P. S. PEPlife: A Repository of the Half-life of Peptides. Sci. Rep. 2016, 6, 36617 10.1038/srep36617. 27819351 PMC5098197

[ref6] Moradi S. V.; Hussein W. M.; Varamini P.; Simerska P.; Toth I. Glycosylation, an effective synthetic strategy to improve the bioavailability of therapeutic peptides. Chem. Sci. 2016, 7, 2492–2425. 10.1039/C5SC04392A. 28660018 PMC5477030

[ref7] Seitz O. Glycopeptide Synthesis and the Effects of Glycosylation on Protein Structure and Activity. ChemBioChem 2000, 1, 214–246. 10.1002/1439-7633(20001117)1:4<214::AID-CBIC214>3.0.CO;2-B. 11828414

[ref8] Grogan M. J.; Pratt M. R.; Marcaurelle L. A.; Bertozzi C. R. Homogeneous glycopeptides and glycoproteins for biological investigation. Annu. Rev. Biochem. 2002, 71, 593–634. 10.1146/annurev.biochem.71.110601.135334. 12045107

[ref9] Taylor C. M. Glycopeptides and glycoproteins: Focus on the glycosidic linkage. Tetrahedron 1998, 54, 11317–11362. 10.1016/S0040-4020(98)00477-3.

[ref10] Stepper J.; Shastri S.; Loo T. S.; Preston J. C.; Novak P.; Man P.; Moore C. H.; Havlíček V.; Patchett M. L.; Norris G. E. Cysteine S-glycosylation, a new post-translational modification found in glycopeptide bacteriocins. FEBS Lett. 2011, 585, 645–650. 10.1016/j.febslet.2011.01.023. 21251913

[ref11] Malkinson J. P.; Falconer R. A. Solid-phase synthesis of C-terminal thio-linked glycopeptides. Tetrahedron Lett. 2002, 43, 9549–9552. 10.1016/S0040-4039(02)02419-X.

[ref12] Wang S. S.; Gao X.; Solar V. D.; Yu X.; Antonopoulos A.; Friedman A. E.; Matich E. K.; Atilla-Gokcumen G. E.; Nasirikenari M.; Lau J. T.; Dell A.; Haslam S. M.; Laine R. A.; Matta K. L.; Neelamegham S. Thioglycosides Are Efficient Metabolic Decoys of Glycosylation that Reduce Selectin Dependent Leukocyte Adhesion. Cell Chem. Biol. 2018, 25, 1519–1532 e5. 10.1016/j.chembiol.2018.09.012. 30344053 PMC6474417

[ref13] Hamachi I.; Nagase T.; Shinkai S. A General Semisynthetic Method for Fluorescent Saccharide-Biosensors Based on a Lectin. J. Am. Chem. Soc. 2000, 122, 12065–12066. 10.1021/ja002044d.

[ref14] Gamblin D. P.; Garnier P.; van Kasteren S.; Oldham N. J.; Fairbanks A. J.; Davis B. G. Glyco-SeS: Selenenylsulfide-Mediated Protein Glycoconjugation—A New Strategy in Post-Translational Modification. Angew. Chem. 2004, 116, 846–851. 10.1002/ange.200352975. 14767951

[ref15] Andre S.; Pei Z.; Siebert H.; Ramstrom O.; Gabius H. Glycosyldisulfides from dynamic combinatorial libraries as O-glycoside mimetics for plant and endogenous lectins: Their reactivities in solid-phase and cell assays and conformational analysis by molecular dynamics simulations. Bioorg. Med. Chem. 2006, 14, 6314–6326. 10.1016/j.bmc.2006.05.045. 16782346

[ref16] Ferrier R. J.; Furneaux R. H.; Tyler P. C. Observations on the possible application of glycosyl disulphides, sulphenic esters, and sulphones in the synthesis of glycosides. Carbohydr. Res. 1977, 58, 397–404. 10.1016/S0008-6215(00)84366-9.

[ref17] Davis B. G.; Ward S. J.; Rendle P. M. Glycosyldisulfides: a new class of solution and solid phase glycosyl donors. Chem. Commun. 2001, 189–190. 10.1039/b008734n.

[ref18] Grayson E. J.; Ward S. J.; Hall A. L.; Rendle P. M.; Gamblin D. P.; Batsanov A. S.; Davis B. G. Glycosyl Disulfides: Novel Glycosylating Reagents with Flexible Aglycon Alteration. J. Org. Chem. 2005, 70, 9740–9754. 10.1021/jo051374j. 16292802

[ref19] Liang C.-F.; Yan M.-C.; Chang T.-C.; Lin C.-C. Synthesis of S-Linked α(2→9) Octasialic Acid via Exclusive α S-Glycosidic Bond Formation. J. Am. Chem. Soc. 2009, 131, 3138–3139. 10.1021/ja808353m. 19215076

[ref20] Liang C.-F.; Kuan T.-C.; Chang T.-C.; Lin C.-C. Stereoselective Synthesis of SLinked α(2→8) and α(2→8)/α(2→9) Hexasialic Acids. J. Am. Chem. Soc. 2012, 134, 16074–16079. 10.1021/ja307797x. 22957651

[ref21] Bernardes G. J. L.; Grayson E. J.; Thompson S.; Chalker J. M.; Errey J. C.; El Oualid F.; Claridge T. D. W.; Davis B. G. From Disulfide- to Thioether-Linked Glycoproteins. Angew. Chem. 2008, 120, 2276–2279. 10.1002/ange.200704381. 18275052

[ref22] Fernández-González M.; Boutureira O.; Bernardes G. J. L.; Chalker J. M.; Young M. A.; Errey J. C.; Davis B. G. Site-selective chemoenzymatic construction of synthetic glycoproteins using endoglycosidases. Chem. Sci. 2010, 1, 709–715. 10.1039/c0sc00265h.

[ref23] Grayson E. J.; Bernardes G. J. L.; Chalker J. M.; Boutureira O.; Koeppe J. R.; Davis B. G. A Coordinated Synthesis and Conjugation Strategy for the Preparation of Homogeneous Glycoconjugate Vaccine Candidates. Angew. Chem., Int. Ed. 2011, 50, 4127–4132. 10.1002/anie.201006327. 21455919

[ref24] Ribeiro Morais G.; Falconer R. A. Glycosyl disulfides: importance, synthesis and application to chemical and biological systems. Org. Biomol. Chem. 2021, 19, 82–100. 10.1039/D0OB02079F. 33188377

[ref25] Ribeiro Morais G.; Springett B. R.; Pauze M.; Schröder L.; Northrop M.; Falconer R. A. Novel strategies for the synthesis of unsymmetrical glycosyl disulfides. Org. Biomol. Chem. 2016, 14, 2749–2754. 10.1039/C6OB00230G. 26853381

[ref26] Ge J. T.; Zhou L.; Zhao F.-L.; Domg H. Straightforward S–S Bond Formation via the Oxidation of S-Acetyl by Iodine in the Presence of N-Iodosuccinimide. J. Org. Chem. 2017, 82, 12613–12623. 10.1021/acs.joc.7b02367. 29084384

[ref27] Kundu M.; Misra A. K. Direct Synthesis of Unsymmetrical Glycosyl Disulfides from Glycosyl Bromides. Eur. J. Org. Chem. 2021, 2021, 3759–3767. 10.1002/ejoc.202100606.

[ref28] Kuan S. L.; Wang T.; Weil T. Site-Selective Disulfide Modification of Proteins: Expanding Diversity beyond the Proteome. Chem. - Eur. J. 2016, 22, 17112–17129. 10.1002/chem.201602298. 27778400 PMC5600100

[ref29] Imberty A.; Perez S. Stereochemistry of the N-glycosylation sites in glycoproteins. Protein Eng., Des. Sel. 1995, 8, 699–709. 10.1093/protein/8.7.699. 8577698

[ref30] Gamblin D. P.; Garnier P.; Ward S. J.; Oldham N. J.; Fairbanks A. J.; Davis B. G. Glycosyl phenylthiosulfonates (glyco-PTS): novel reagents for glycoprotein synthesis. Org. Biomol. Chem. 2003, 1, 3642–3644. 10.1039/b306990g. 14649893

[ref31] Macindoe W. M.; van Oijen A. H.; Boons G.-J. A unique and highly facile method for synthesising disulfide linked neoglycoconjugates: a new approach for remodelling of peptides and proteins. Chem. Commun. 1998, 7, 847–848. 10.1039/a708701b.

[ref32] Aversa M. C.; Barattucci A.; Bonaccorsi P. Efficient Synthesis of Unsymmetrical Disulfides through Sulfenic Acids. Eur. J. Org. Chem. 2009, 2009, 6355–6359. 10.1002/ejoc.200900986.

[ref33] Illyés T.-Z.; Szabo T.; Szilagyi L. Glycosylation via mixed disulfide formation using glycosylthio-phthalimides and -succinimides as glycosylsulfenyl-transfer reagents. Carbohydr. Res. 2011, 346, 1622–1627. 10.1016/j.carres.2011.04.020. 21571258

[ref34] Ribeiro Morais G.; Falconer R. A. Efficient one-pot synthesis of glycosyl disulfides. Tetrahedron Lett. 2007, 48, 7637–7641. 10.1016/j.tetlet.2007.08.106.

[ref35] Bulaj G.; Kortemme T.; Goldenberg D. P. Ionization–Reactivity Relationships for Cysteine Thiols in Polypeptides. Biochemistry 1998, 37, 8965–8972. 10.1021/bi973101r. 9636038

[ref36] Cao H. T.; Grée R. DEAD-(cat)ZnBr2 an efficient system for the oxidation of alcohols to carbonyl compounds. Tetrahedron Lett. 2009, 50, 1493–1494. 10.1016/j.tetlet.2009.01.080.

[ref37] Martín-Santamaría S.; André S.; Buzamet E.; Caraballo R.; Fernández-Cureses G.; Morando M.; Ribeiro J. P.; Ramírez-Gualito K.; De Pascual-Teresa B.; Cañada F. J.; Menéndez M.; Ramström O.; Jiménez-Barbero J.; Solís D.; Gabius H.-J. Symmetric dithiodigalactoside: Strategic combination of binding studies and detection of selectivity between a plant toxin and human lectins. Org. Biomol. Chem. 2011, 9, 5445–5455. 10.1039/c0ob01235a. 21660340

[ref38] Fujihira T.; Chida M.; Kamijo H.; Takido T.; Seno M. Novel synthesis of 1-thioglycopyranoses via thioiminium salts. J. Carbohydr. Chem. 2002, 21, 287–292. 10.1081/CAR-120013495.

[ref39] Morais G. R.; Humphrey A. J.; Falconer R. A. A facile preparation of trehalose analogues: 1,1-thiodisaccharides. Carbohydr. Res. 2009, 344, 1039–1045. 10.1016/j.carres.2009.03.017. 19368901

[ref40] Lloyd-Williams P.; Albericio F.; Giralt E. Convergent solid-phase peptide synthesis. Tetrahedron 1993, 49, 11065–11133. 10.1016/S0040-4020(01)81800-7. 9353728

