# Supplementary material for: Chemoselective Solution- and Solid-Phase Synthesis of Disulfide-Linked Glycopeptides
Source: J Org Chem. 2022 Oct 20;87(21):14026–36. doi: 10.1021/acs.joc.2c01651 (PMC9638999; doi:10.1021/acs.joc.2c01651)

Supplementary information

Chemoselective solution- and solid-phase synthesis of disulfide-linked glycopeptides

Katreen A. F. Banisalman,[‡] Athina Polykandritou,[‡] Francis M. Barnieh, Goreti Ribeiro Morais* and Robert A. Falconer*

Institute of Cancer Therapeutics, Faculty of Life Sciences, University of Bradford, Bradford BD7 1DP, U.K.

Email: g.ribeiromorais@bradford.ac.uk; r.a.falconer1@bradford.ac.uk

Contents:

NMR data for compound **5** (p. S2)

NMR data for compound **6** (p. S3)

NMR data for compound **7** (p. S5)

NMR data for compound **10** (p. S6)

NMR data for compound **13** (p. S8)

NMR data for thio-glucose sodium salt (**15**) (p. S9)

NMR data reaction of thio-glucose sodium salt plus DEAD (p. S11)

NMR data reaction of thio-glucose sodium salt plus DEAD followed by thiophenol (p. S13)

NMR data for compound **17** (p. S15)

NMR data for compound **18** (p. S17)

NMR data for compound **19** (p. S18)

NMR data for compound **20** (p. S20)

NMR data for compound **21** (p. S21)

NMR data for compound **22** (p. S23)

HPLC chromatogram of crude peptides, and corresponding mass spectra (p. S25-S57)

^{1}H NMR compound **5** (400 MHz, $CDCl_3$)

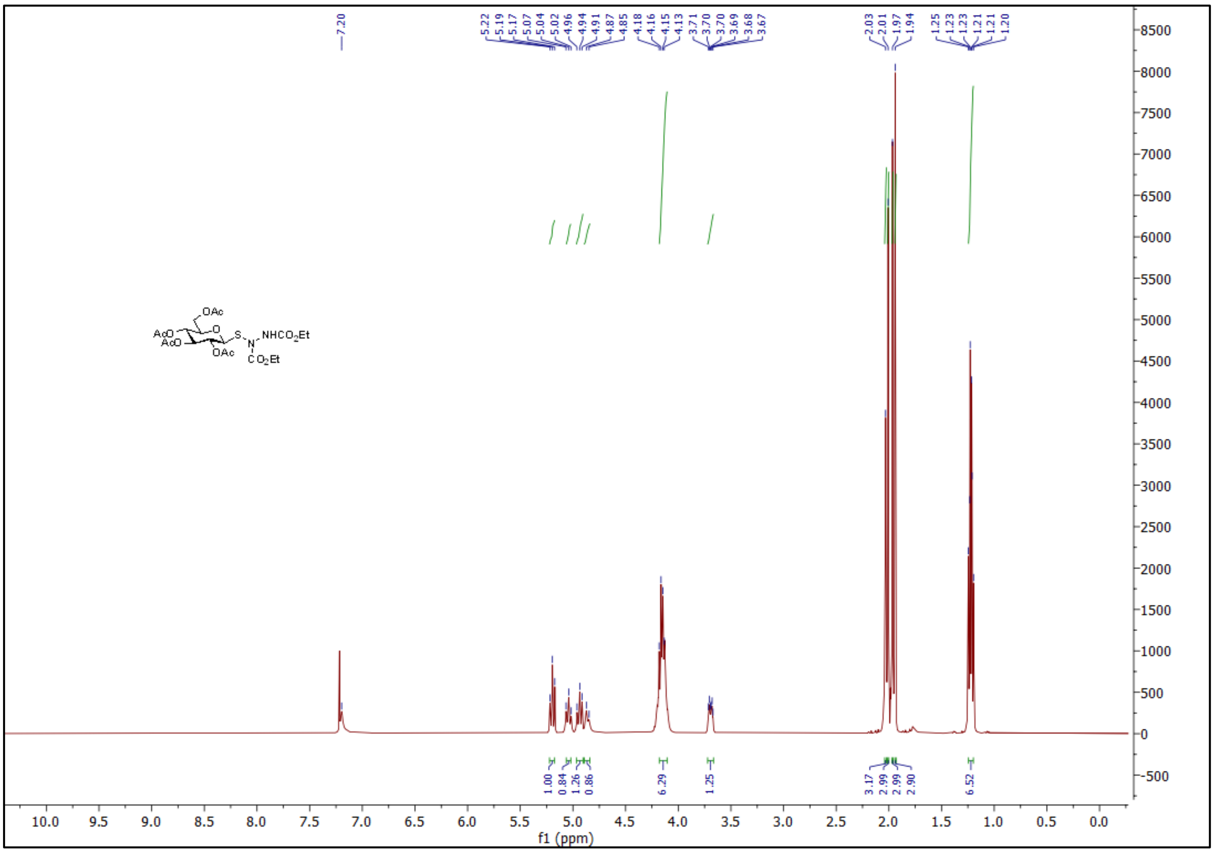

COSY NMR compound **5**

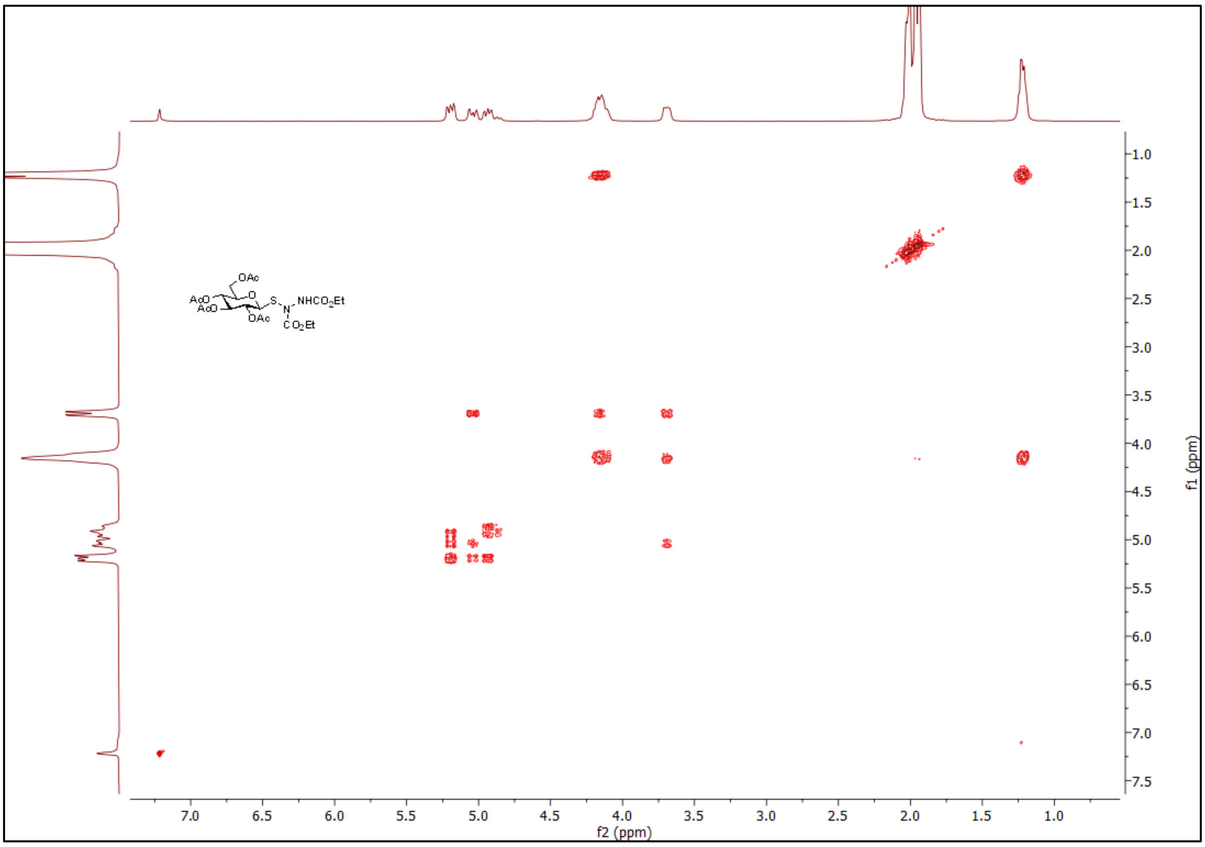

$^{13}C\{^{1}H\}$ NMR of compound **5** (100 MHz, $CDCl_3$)

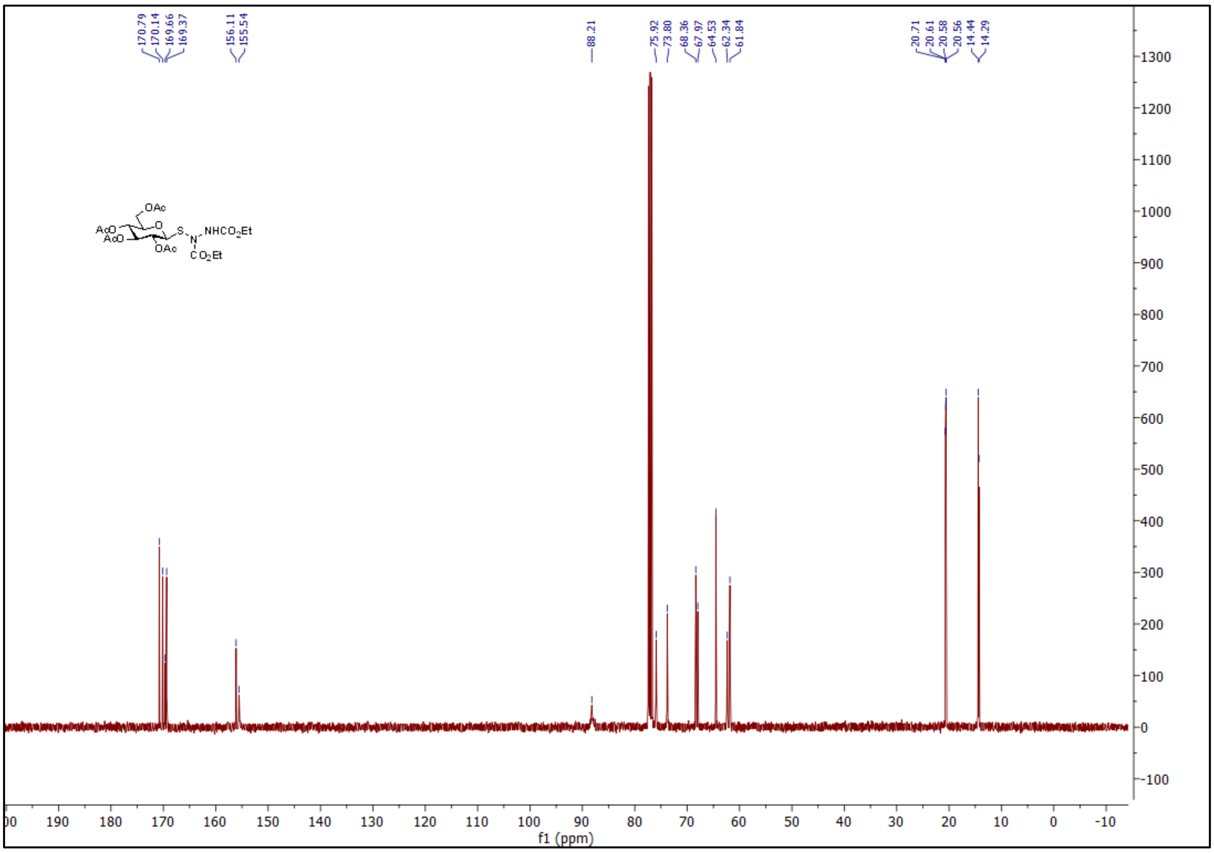

^{1}H NMR compound **6** (400 MHz, $CDCl_3$)

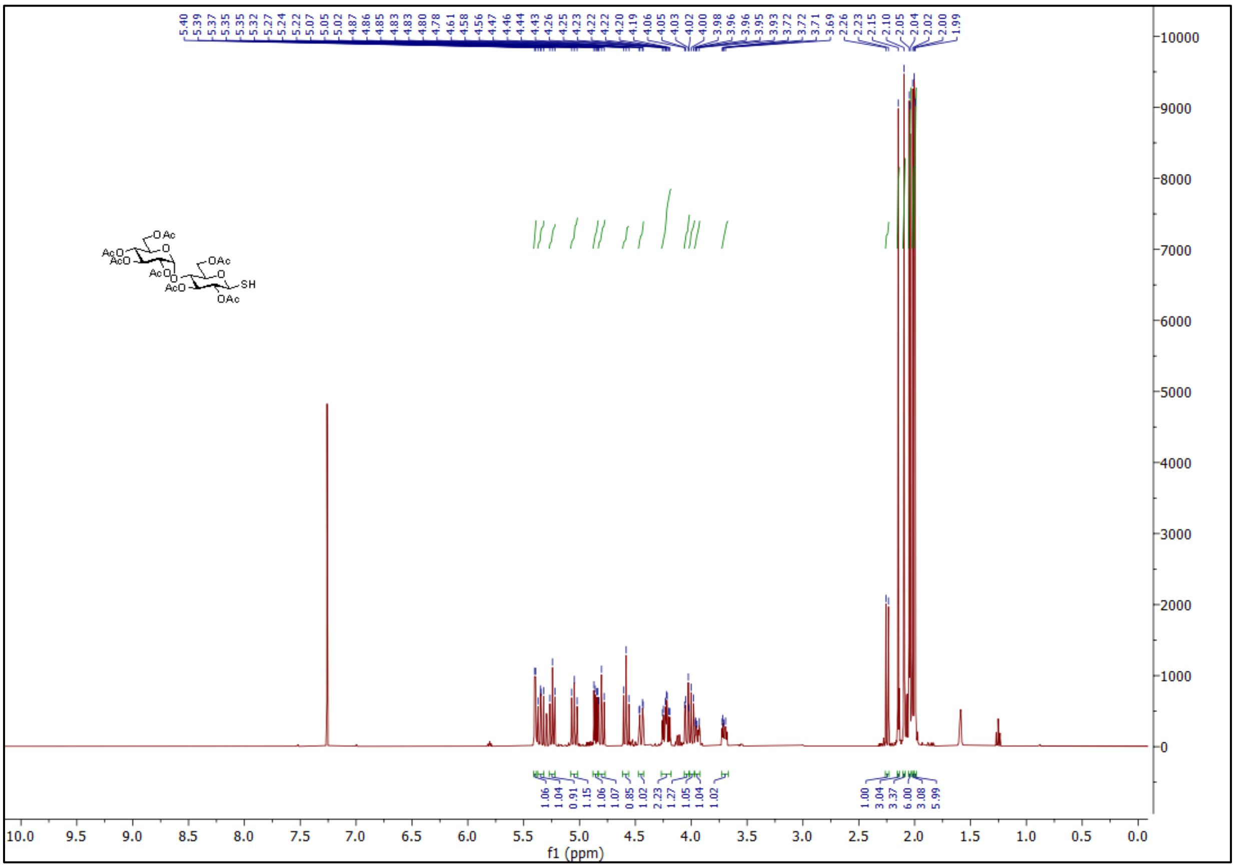

COSY NMR compound **6**

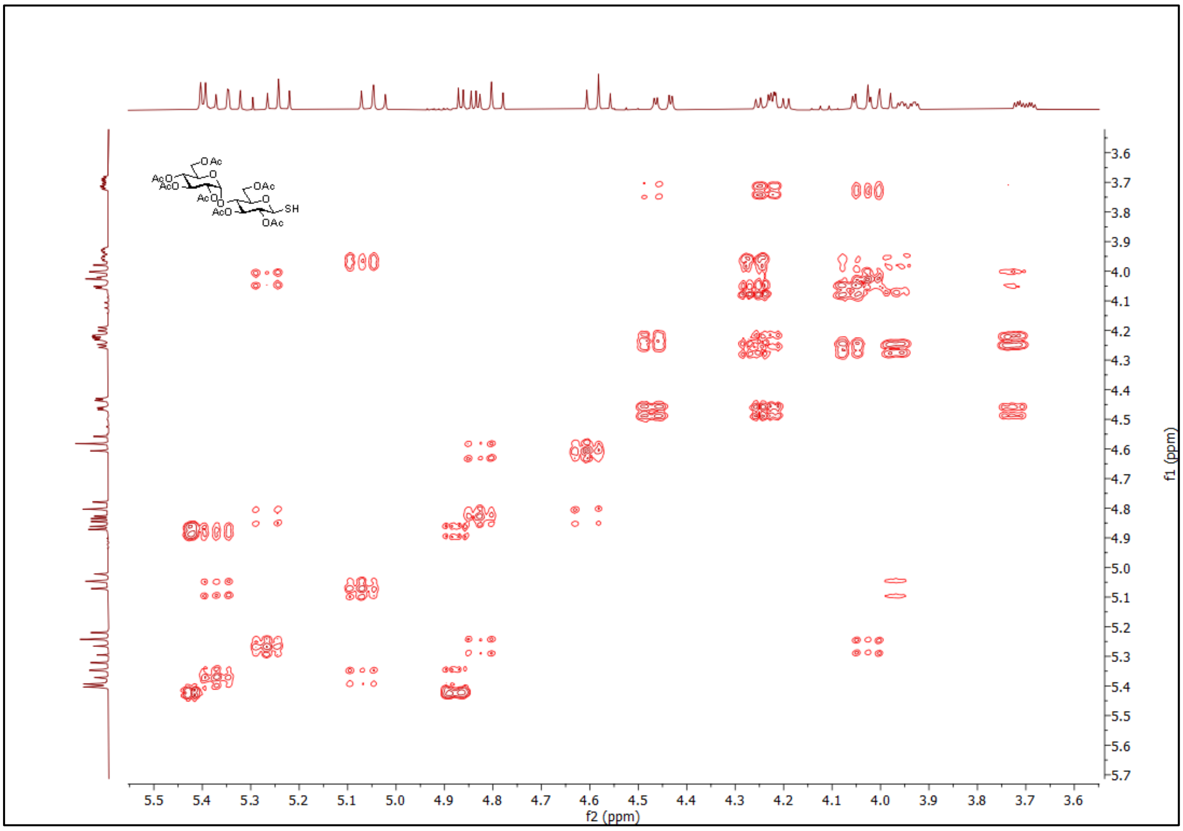

$^{13}C\{^1H\}$ NMR of compound **6** (100 MHz, $CDCl_3$)

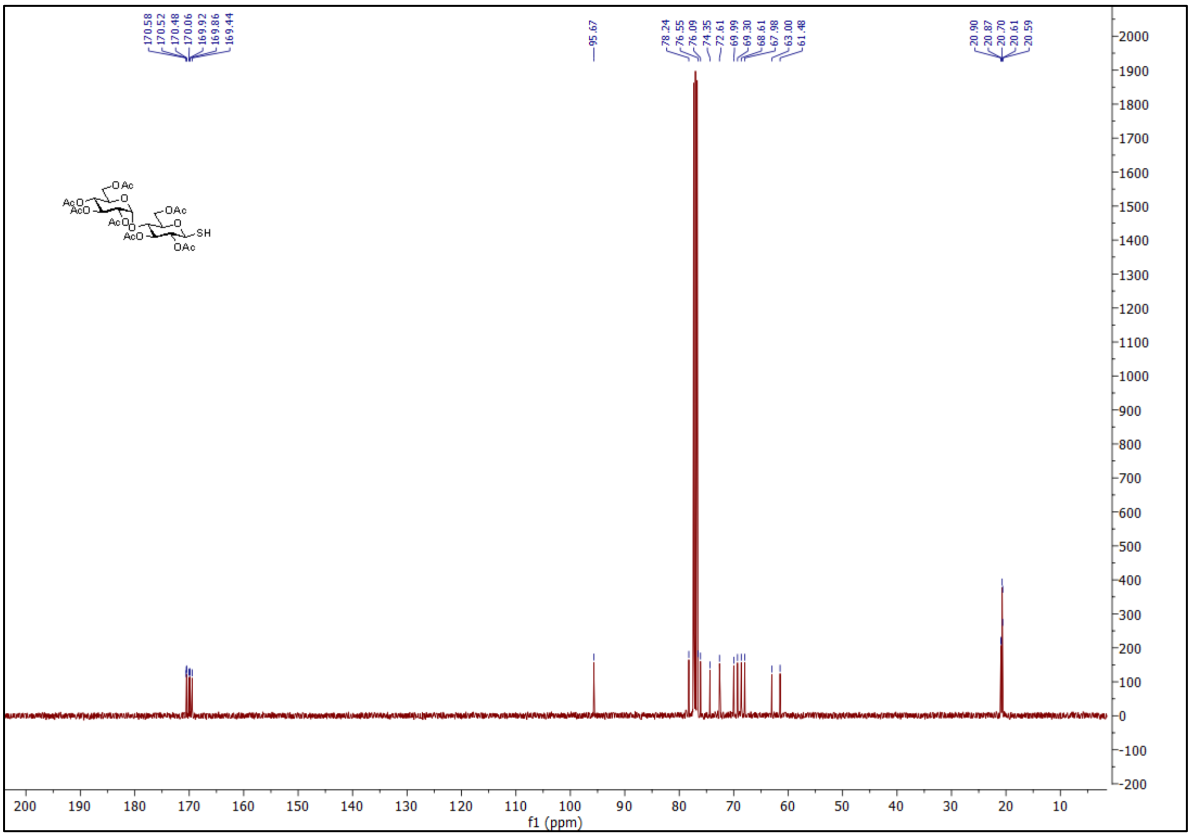

^{1}H NMR compound **7** (400 MHz, $CDCl_3$)

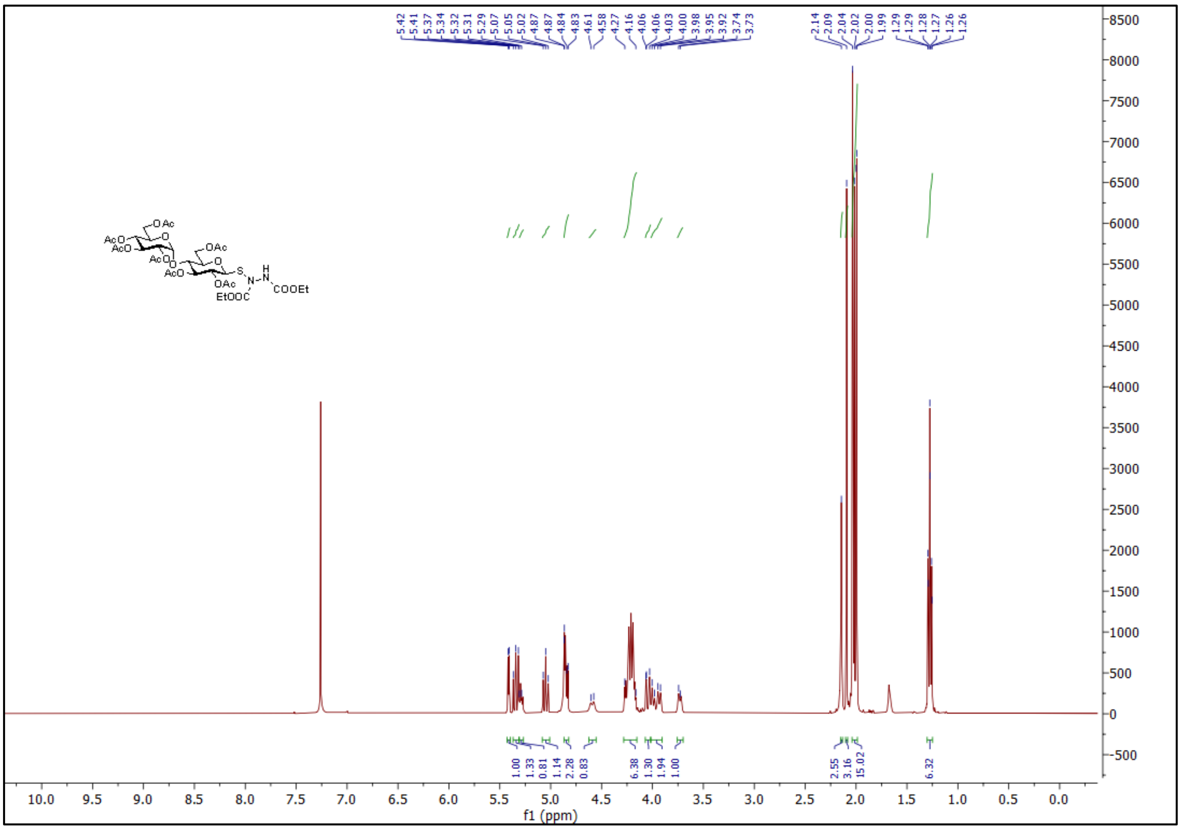

COSY NMR compound **7**

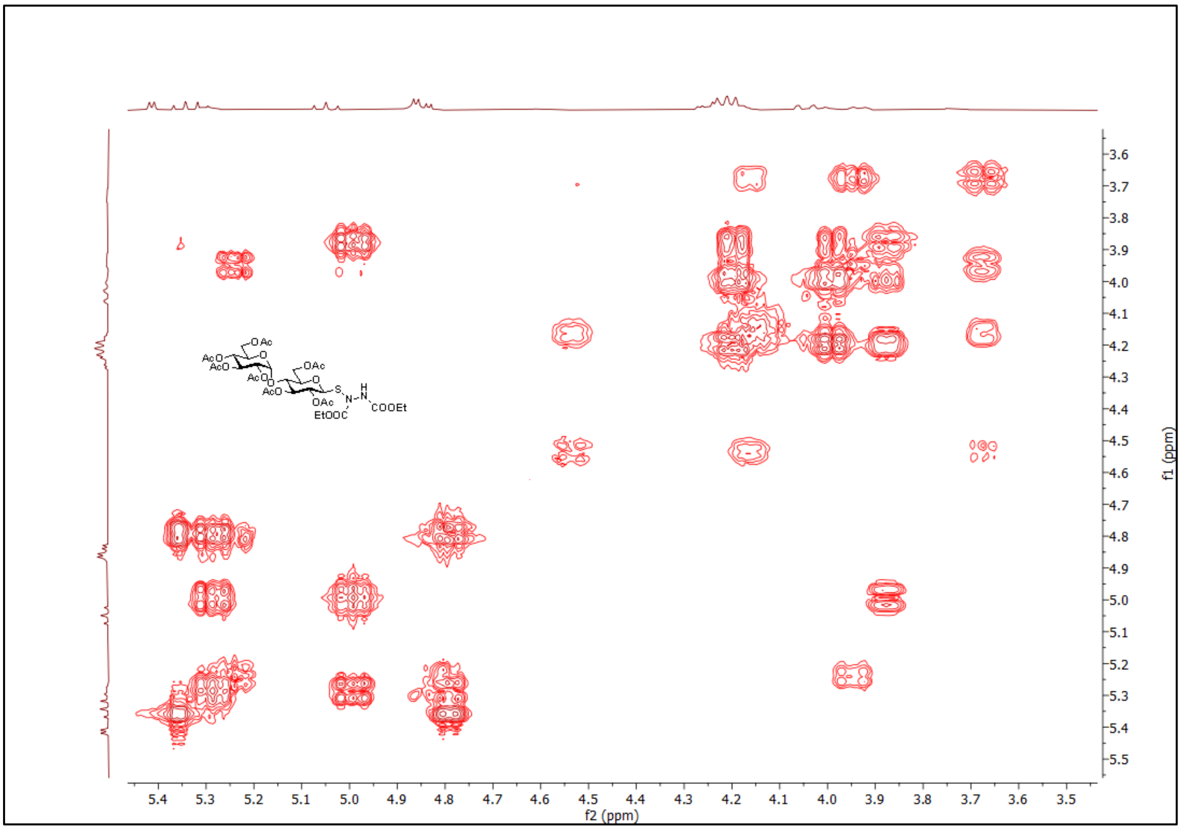

$^{13}C\{^1H\}$ NMR of compound **7** (100 MHz, $CDCl_3$)

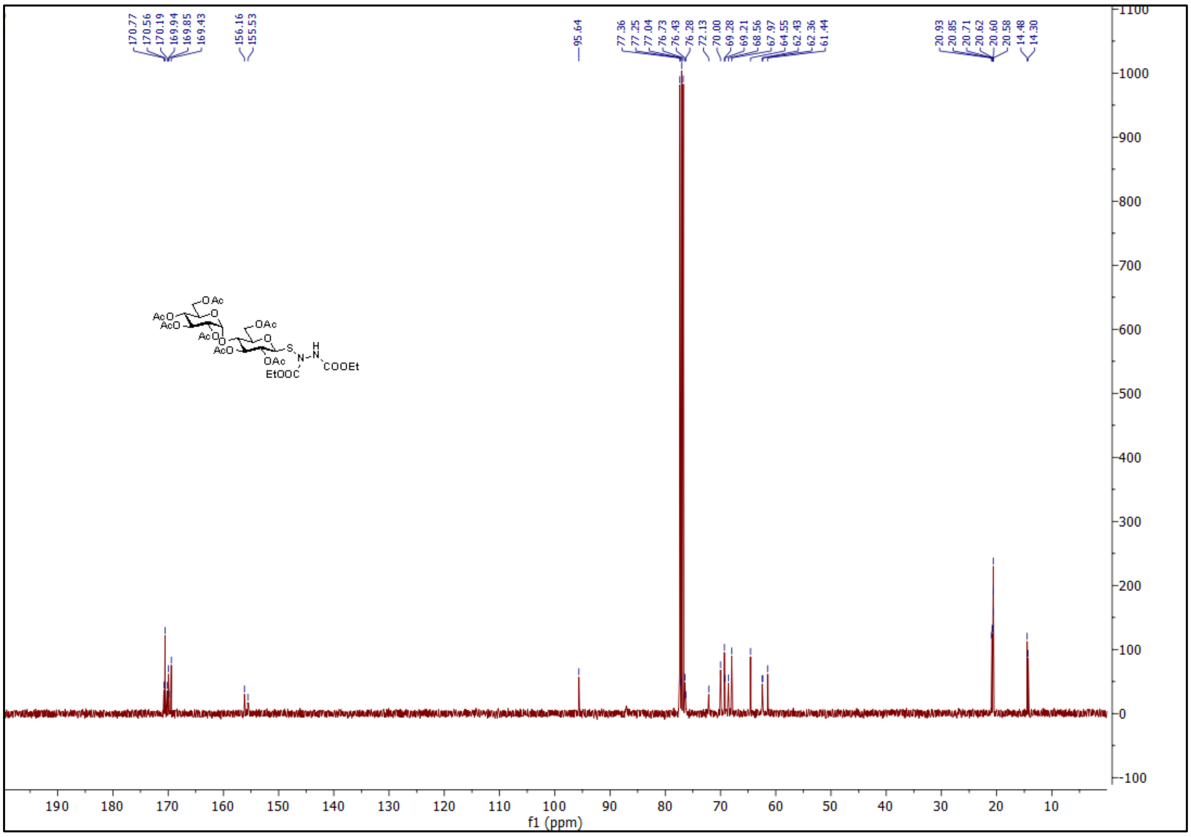

^1H NMR compound **10** (400 MHz, $CDCl_3$)

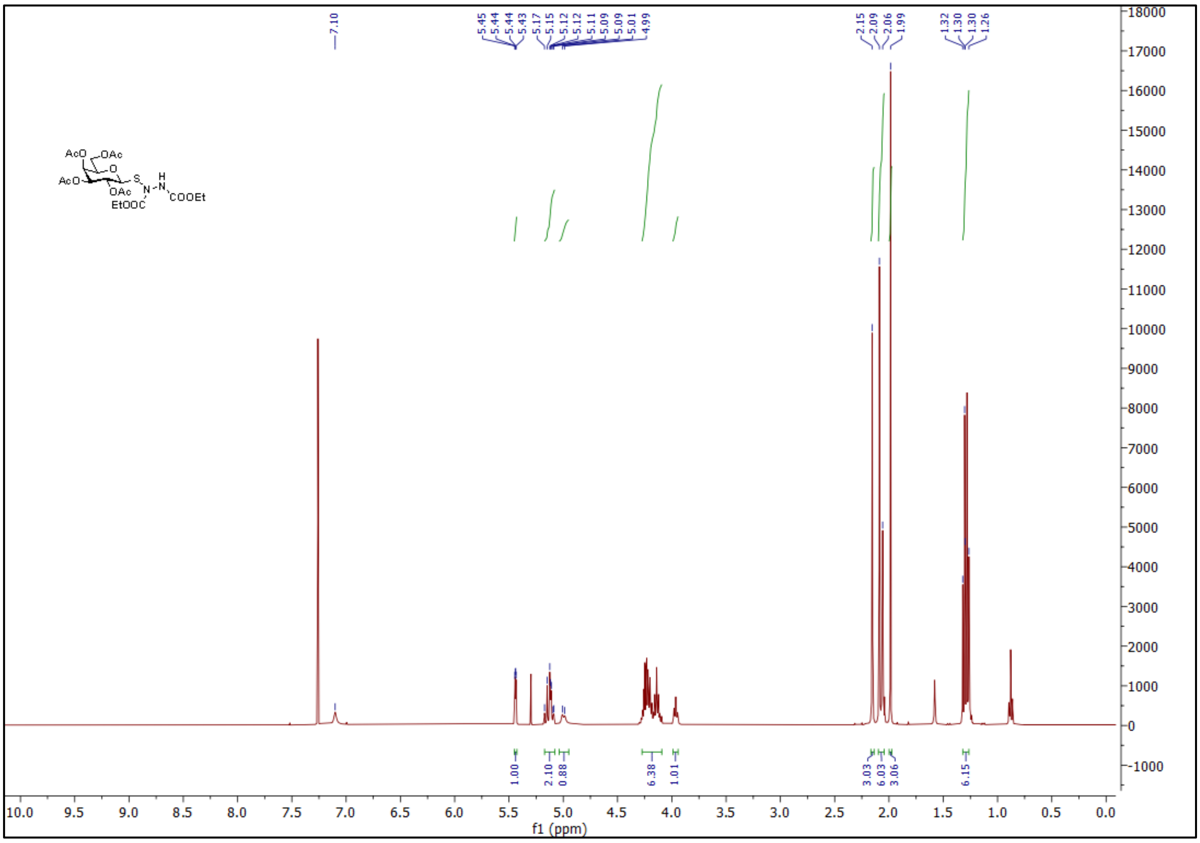

COSY NMR compound **10**

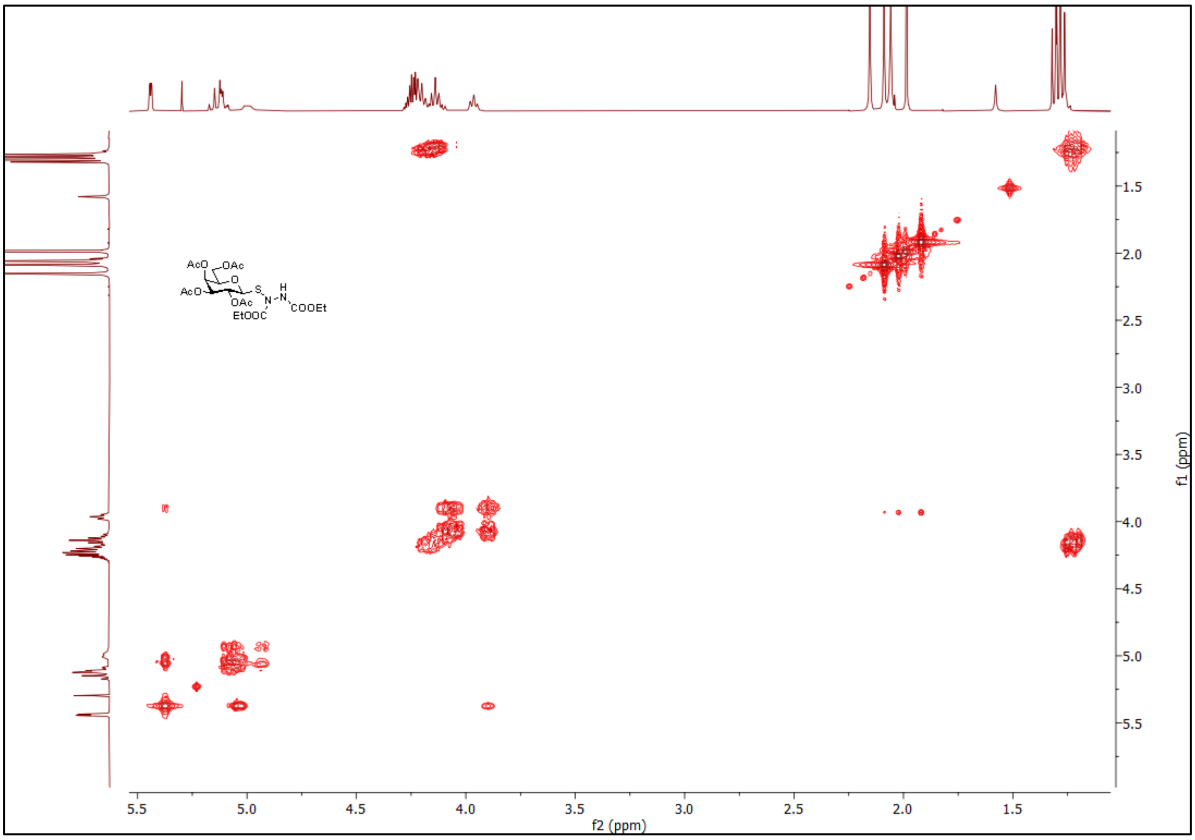

$^{13}C\{^{1}H\}$ NMR of compound **10** (100 MHz, $CDCl_3$)

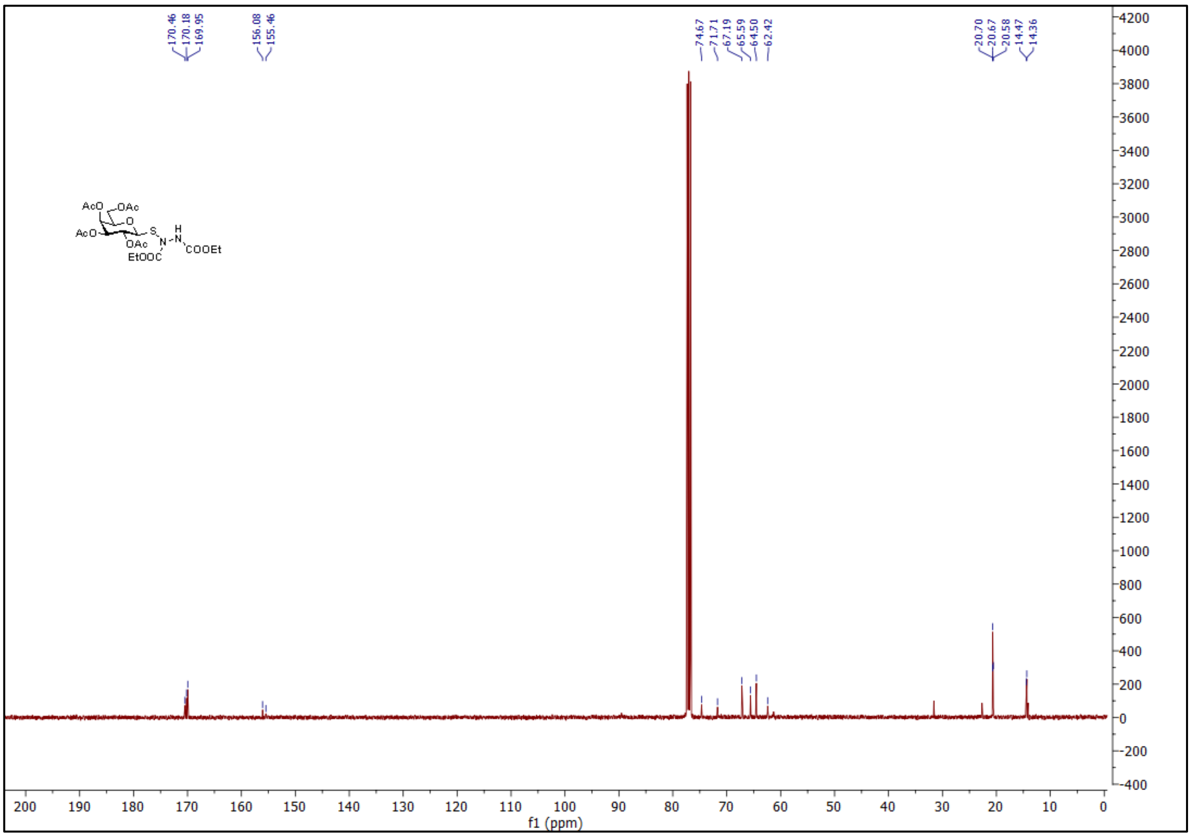

^{1}H NMR of compound **13** (400 MHz, $CDCl_3$)

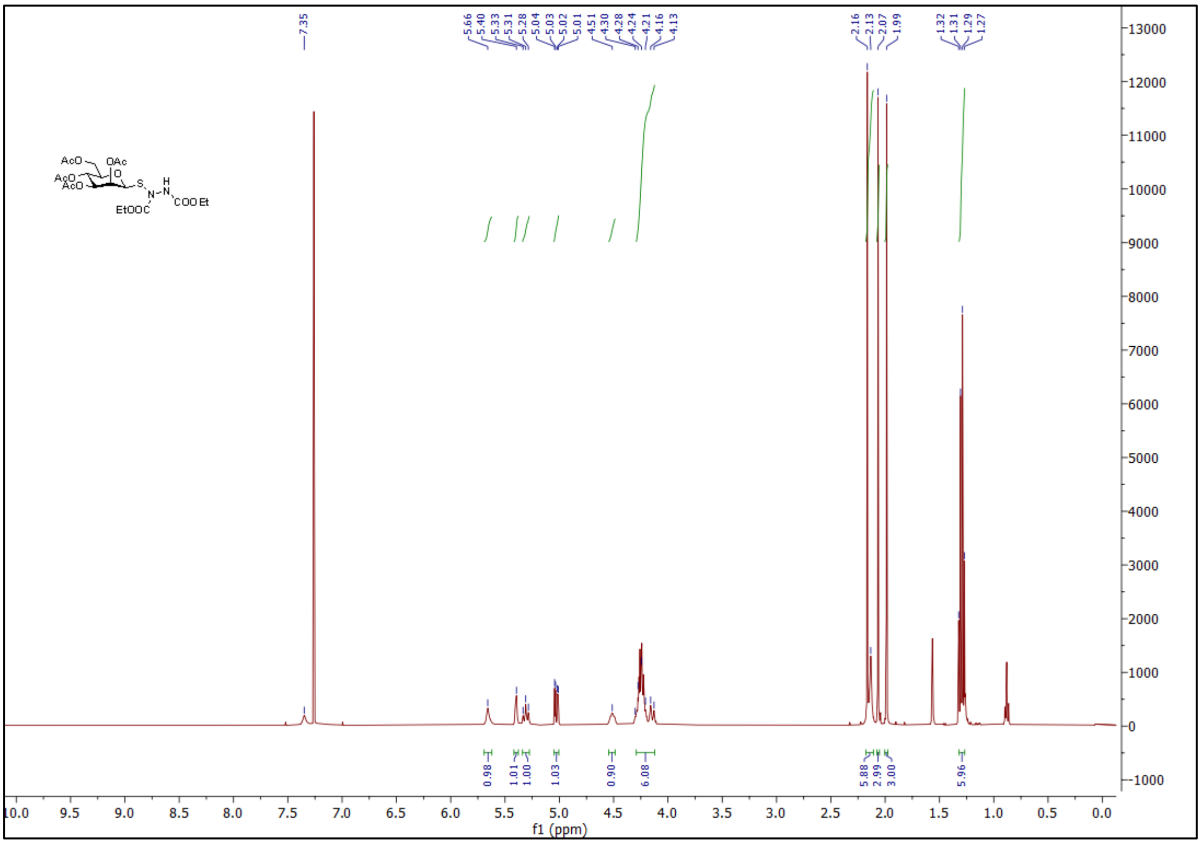

COSY NMR compound **13**

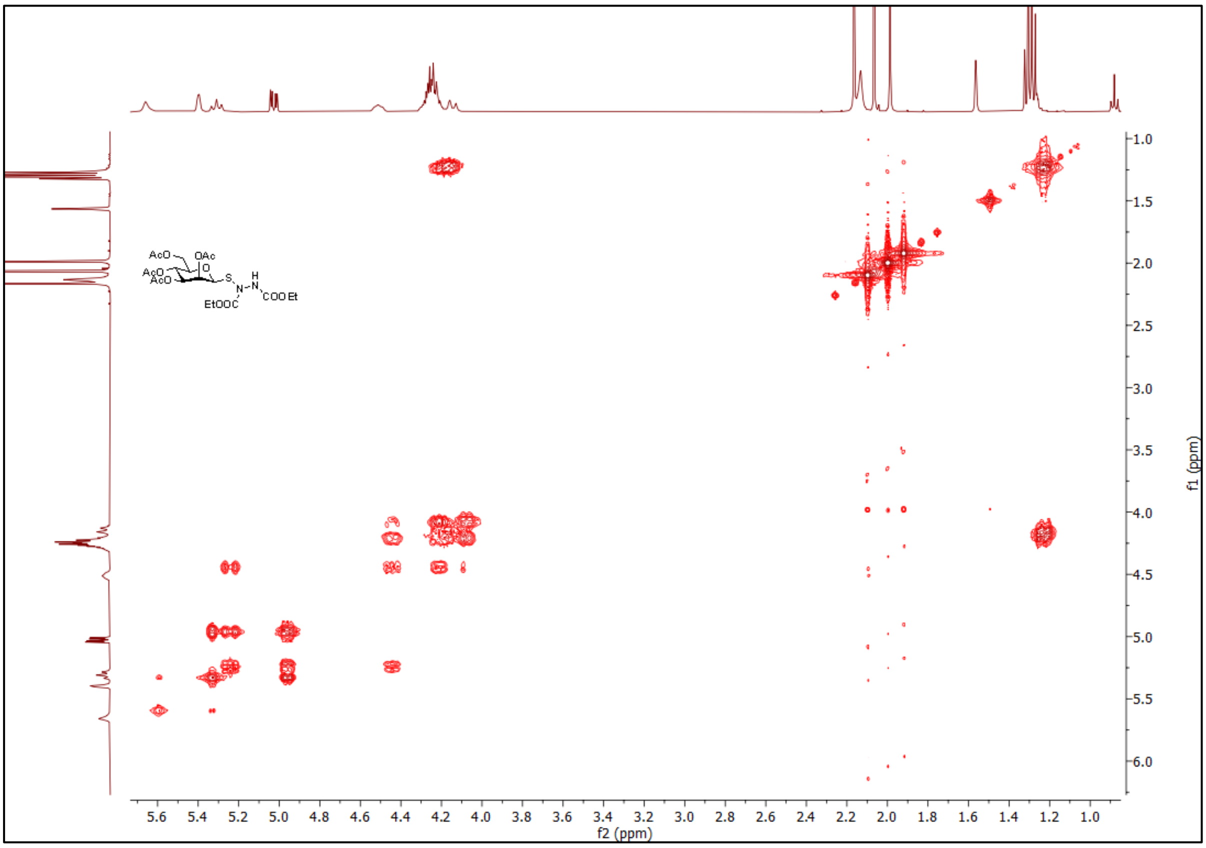

$^{13}C\{^{1}H\}$ NMR of compound **13** (100 MHz, $CDCl_3$)

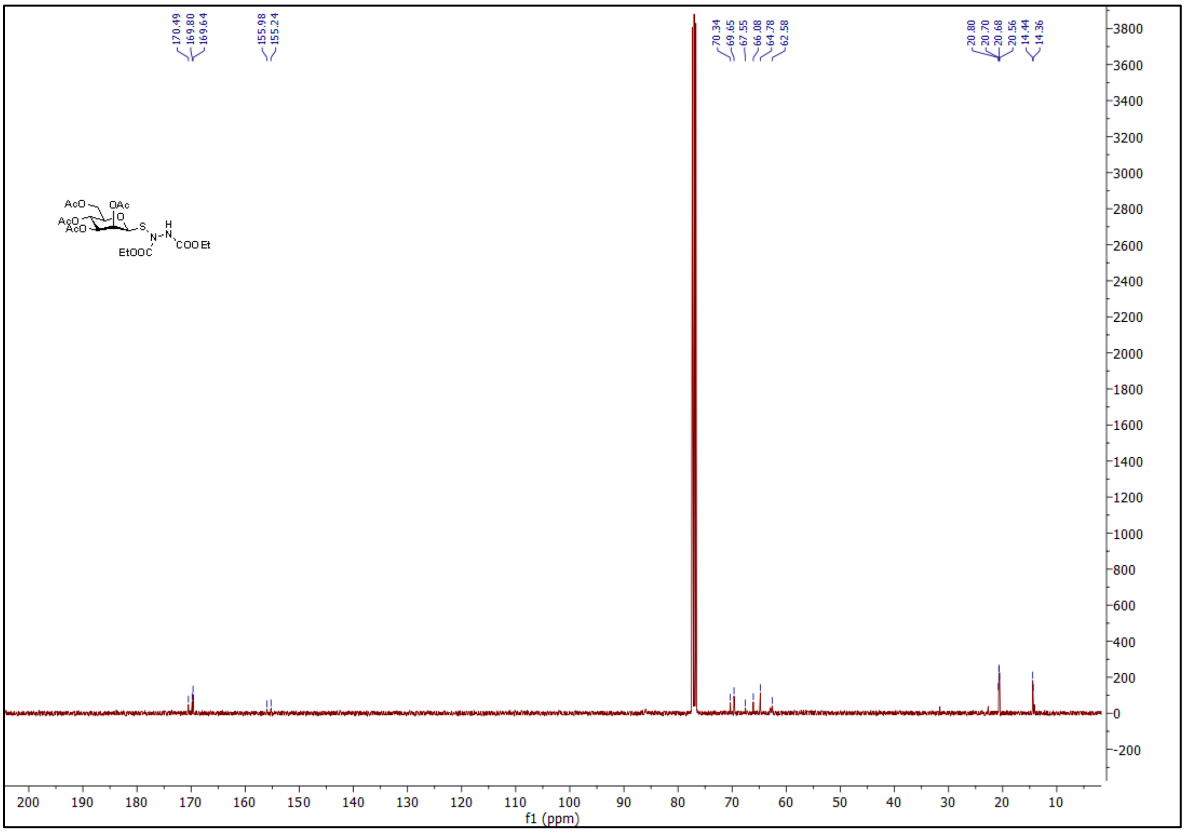

^{1}H NMR of thioglucose(OH)$_4$ sodium salt (**15**) (400 MHz, CD_3OD)

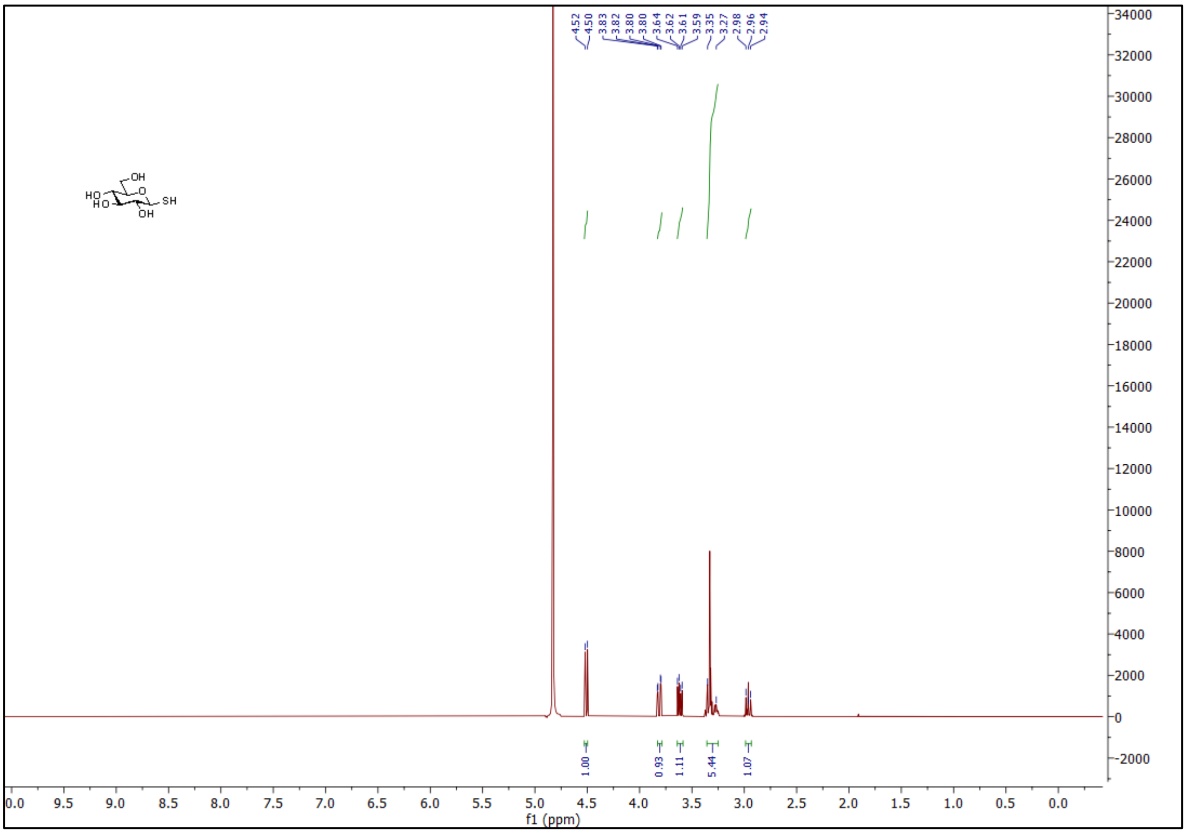

COSY NMR of thioglucose(OH)$_4$ sodium salt (CD_3OD) (**15**)

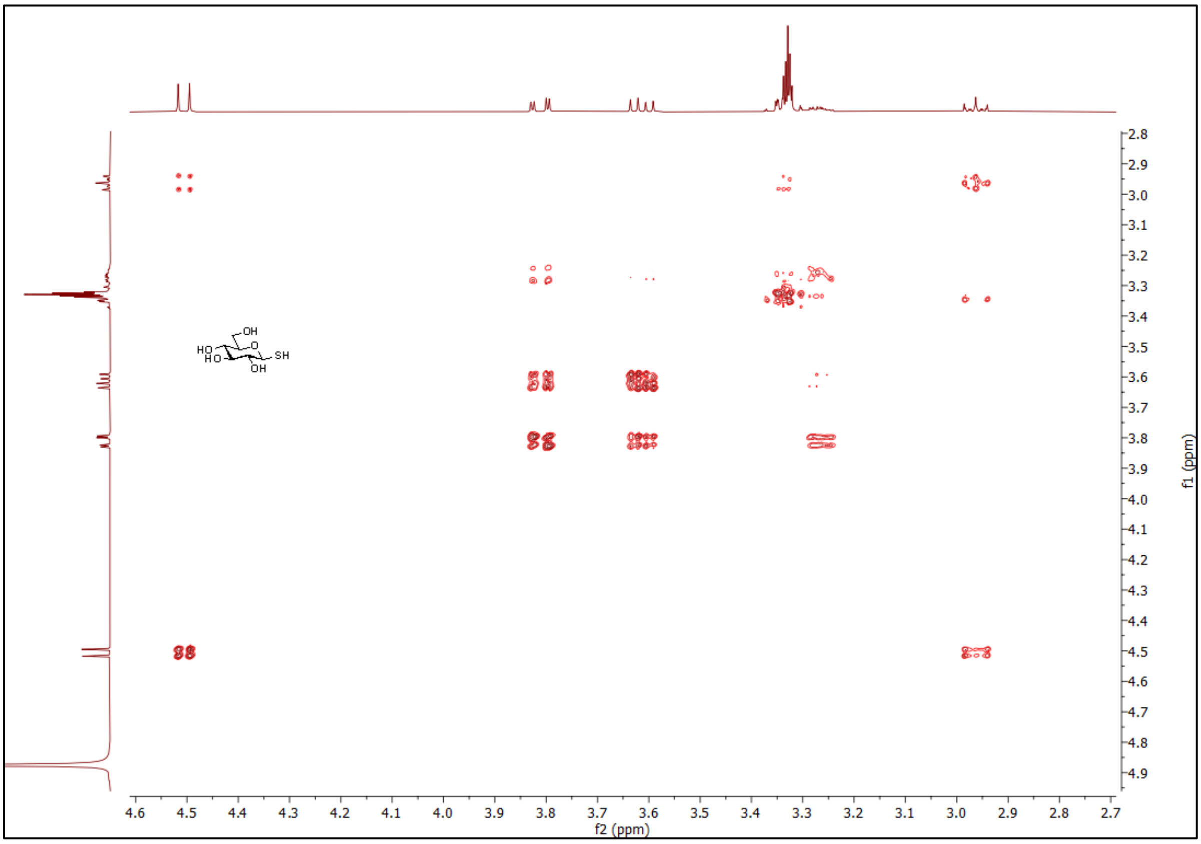

HSQC NMR of thioglucose(OH)$_4$ sodium salt (CD_3OD) (**15**)

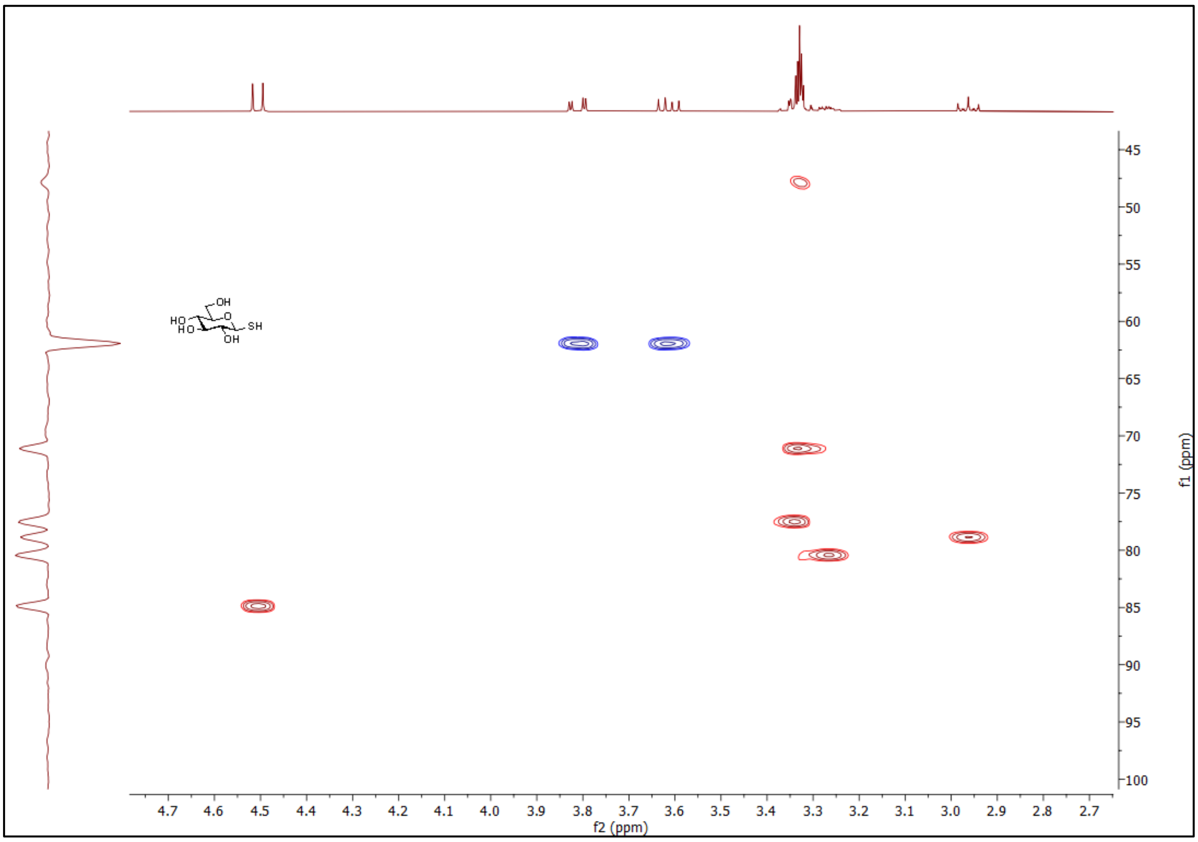

$^{13}C\{^{1}H\}$ NMR of thioglucose(OH)$_4$ sodium salt (CD_3OD) (**15**) (100 MHz, $CDCl_3$)

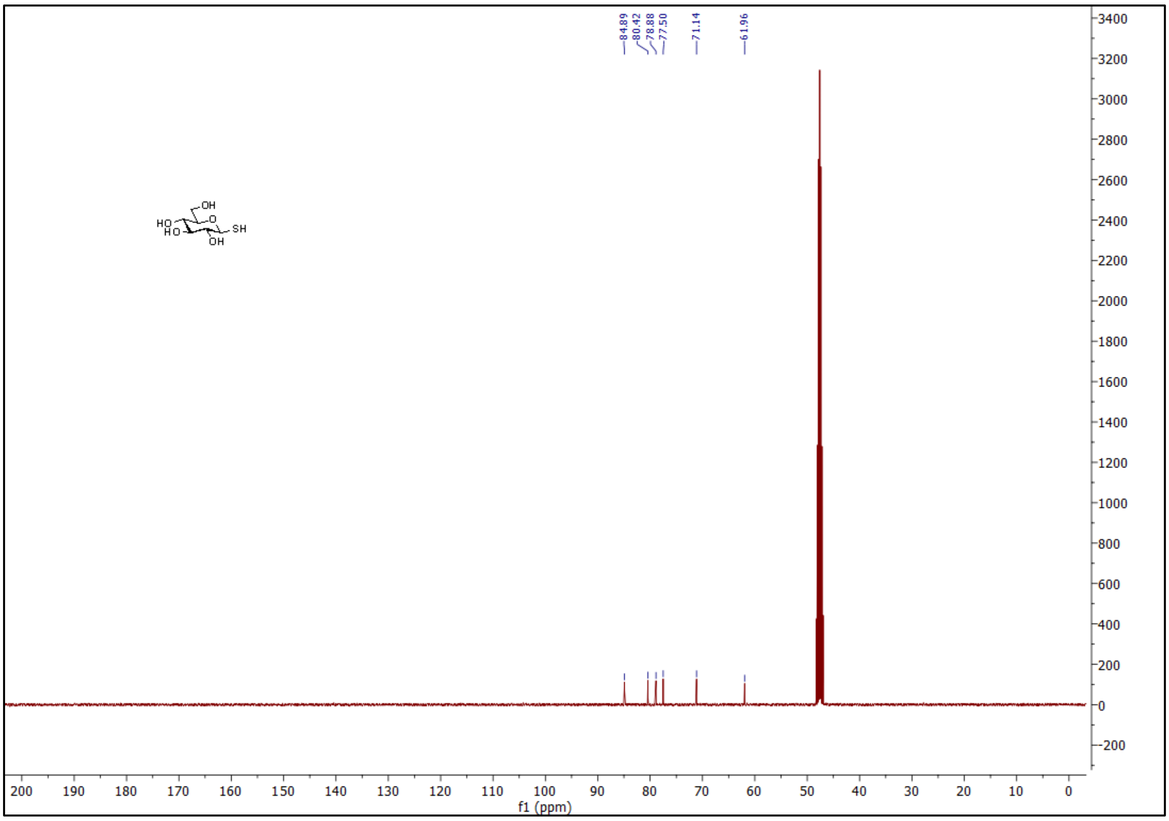

^{1}H NMR of reaction of thioglucose(OH)$_4$ with DEAD (40% in toluene) (400 MHz, CD_3OD)

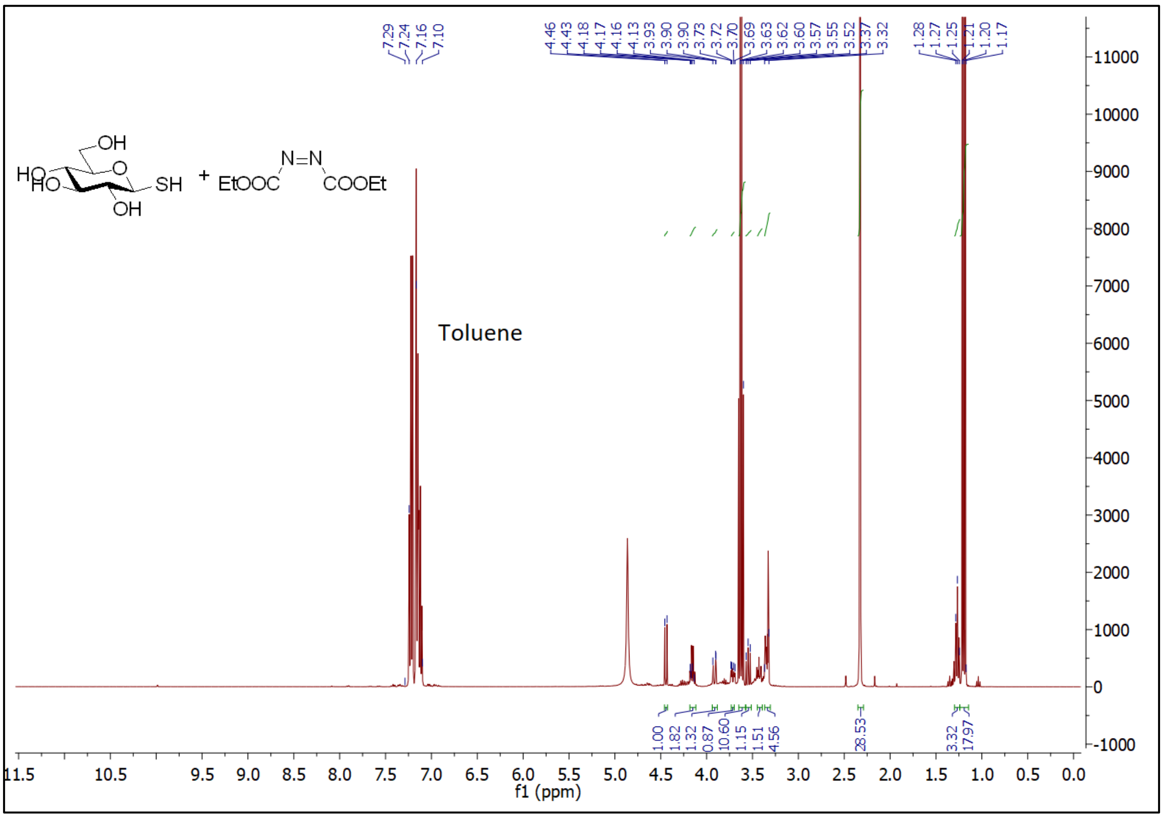

COSY NMR of reaction of thioglucose(OH)$_4$ with DEAD (40% in toluene) in CD_3OD

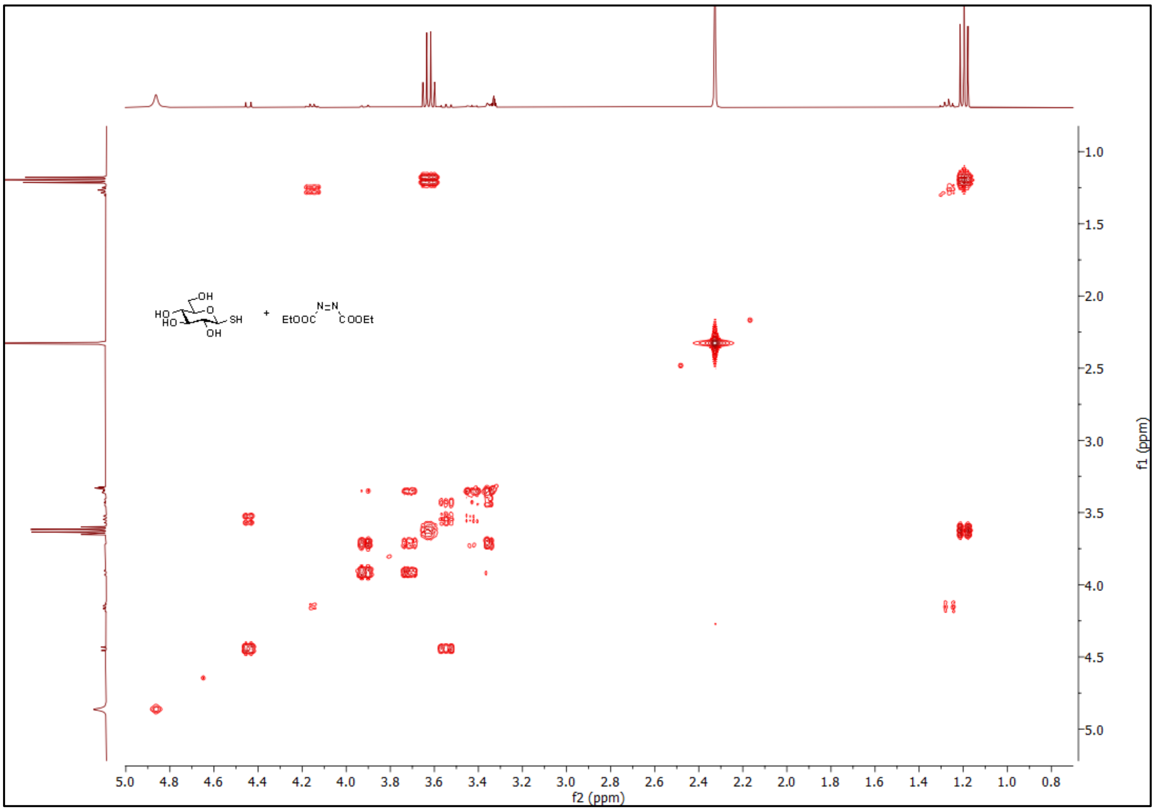

HSQC NMR of reaction of thioglucose(OH)$_4$ with DEAD (40% in toluene) in CD_3OD

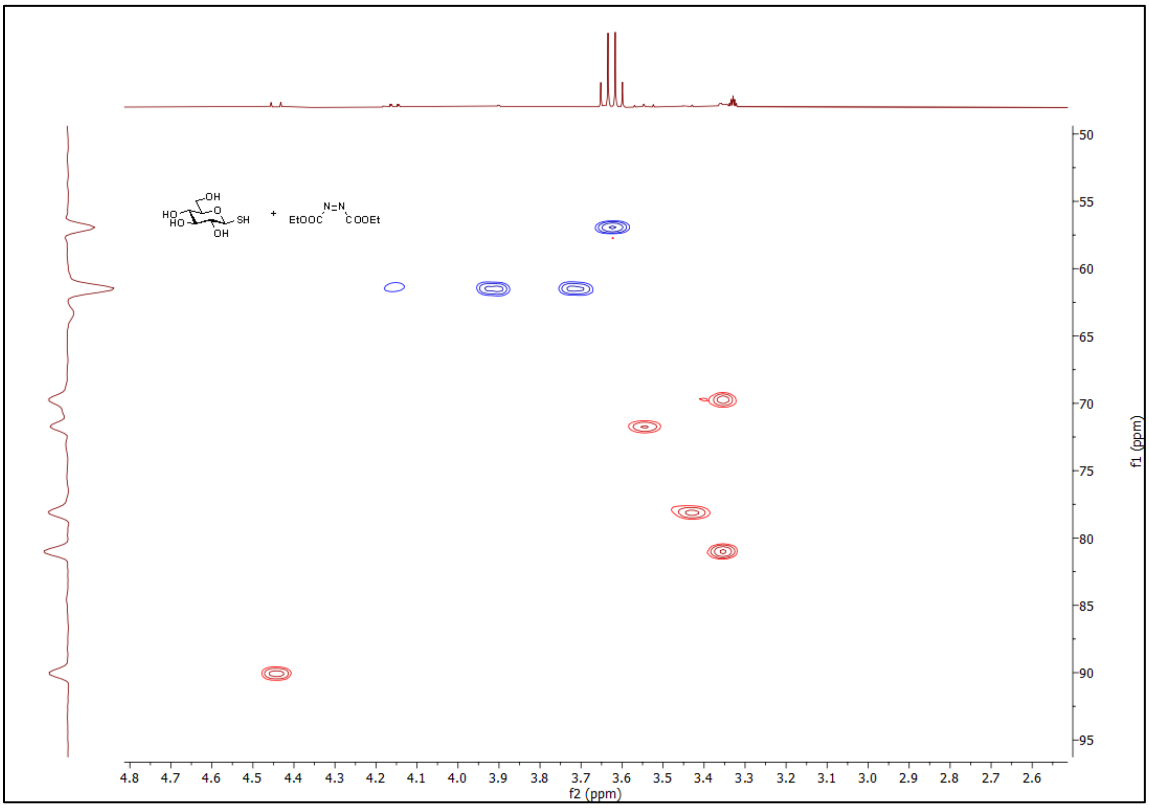

$^{13}C\{^1H\}$ NMR of reaction of thioglucose(OH)$_4$ with DEAD (40% in toluene) (100 MHz, CD_3OD)

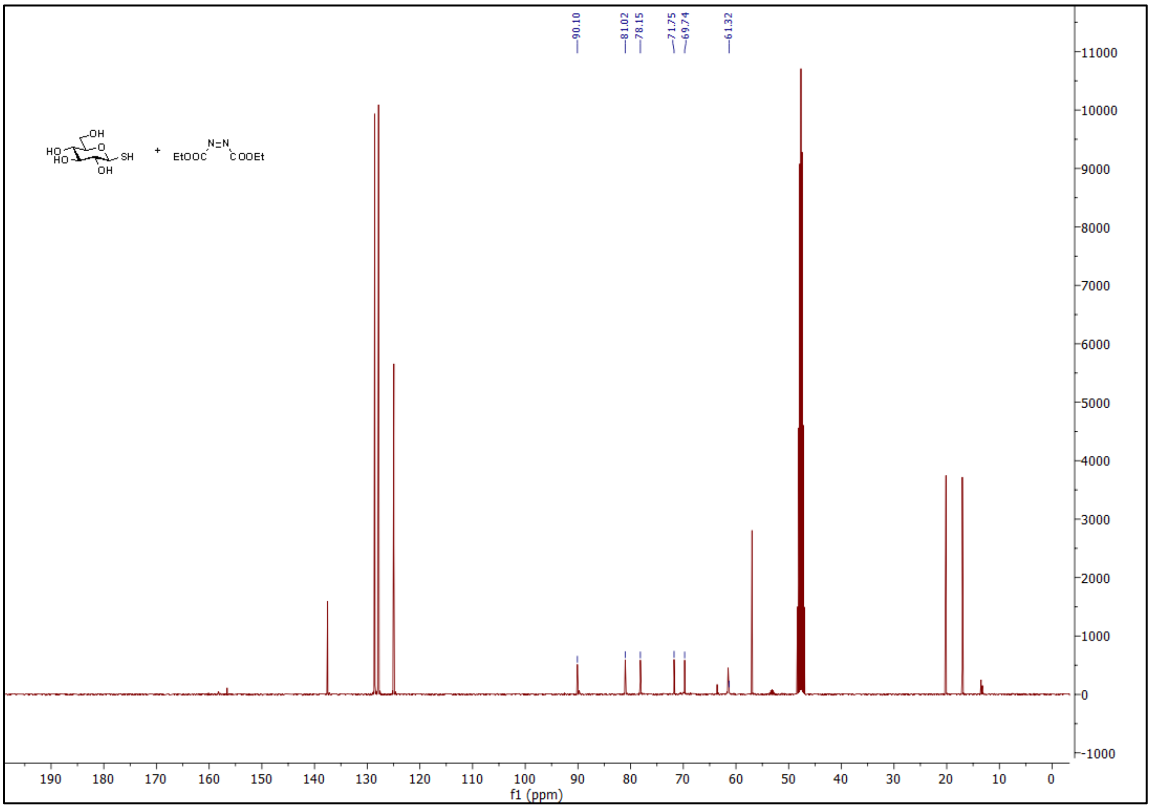

^1H NMR of thioglucose(OH)$_4$ plus DEAD followed by thiophenol (1h) (400 MHz,CD_3OD)

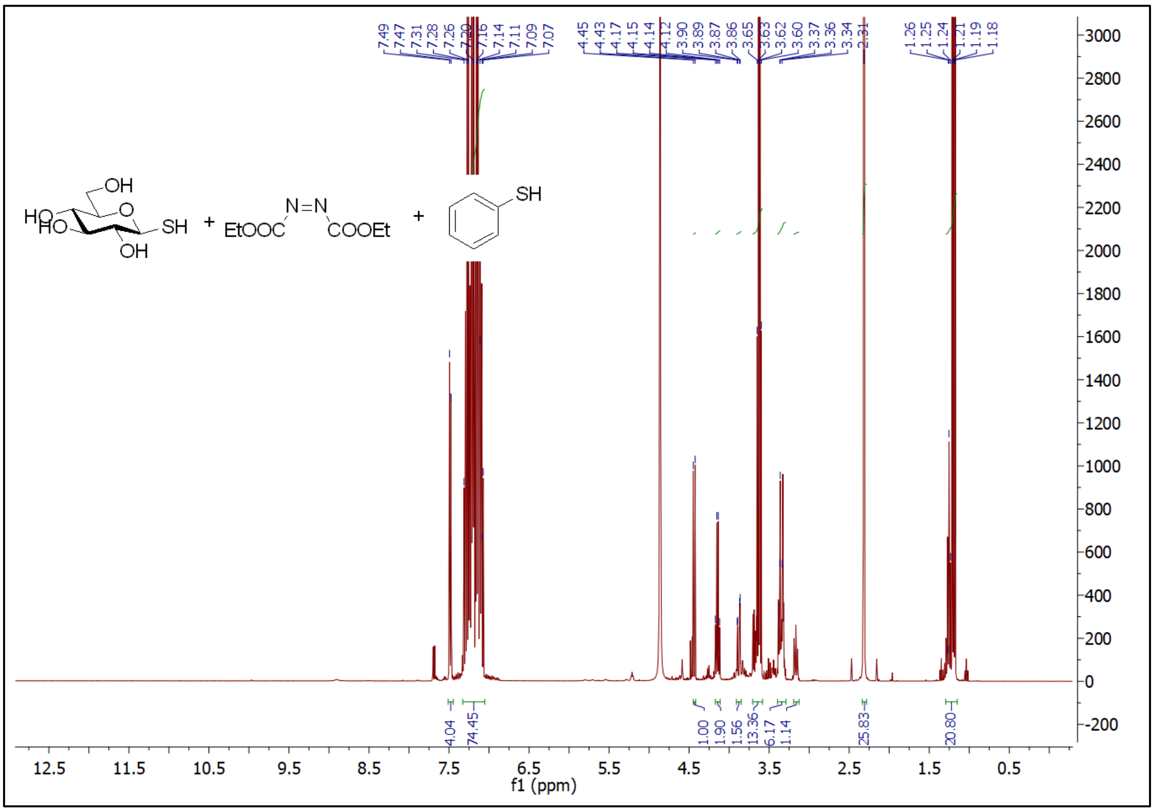

COSY NMR of thioglucose(OH)$_4$ plus DEAD followed by thiophenol in CD_3OD (1h)

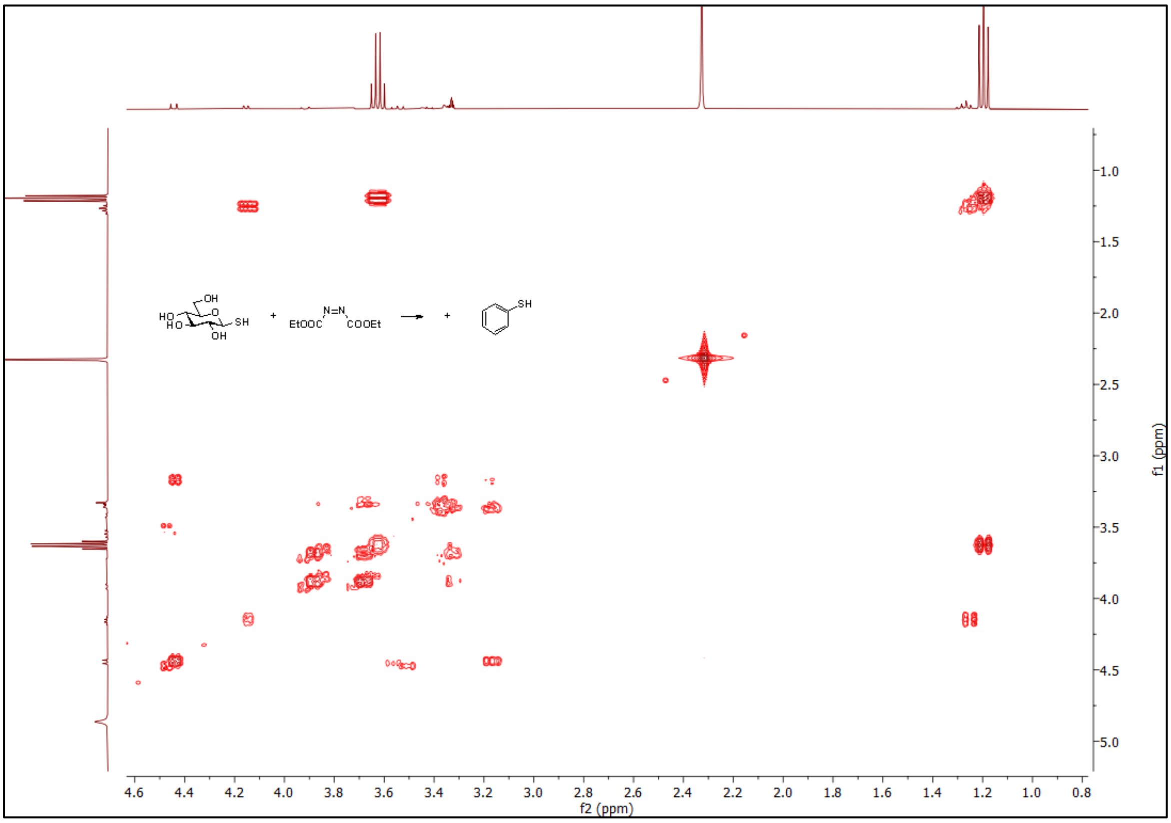

HSQC NMR of thioglucose(OH)$_4$ plus DEAD followed by thiophenol in CD_3OD (1h)

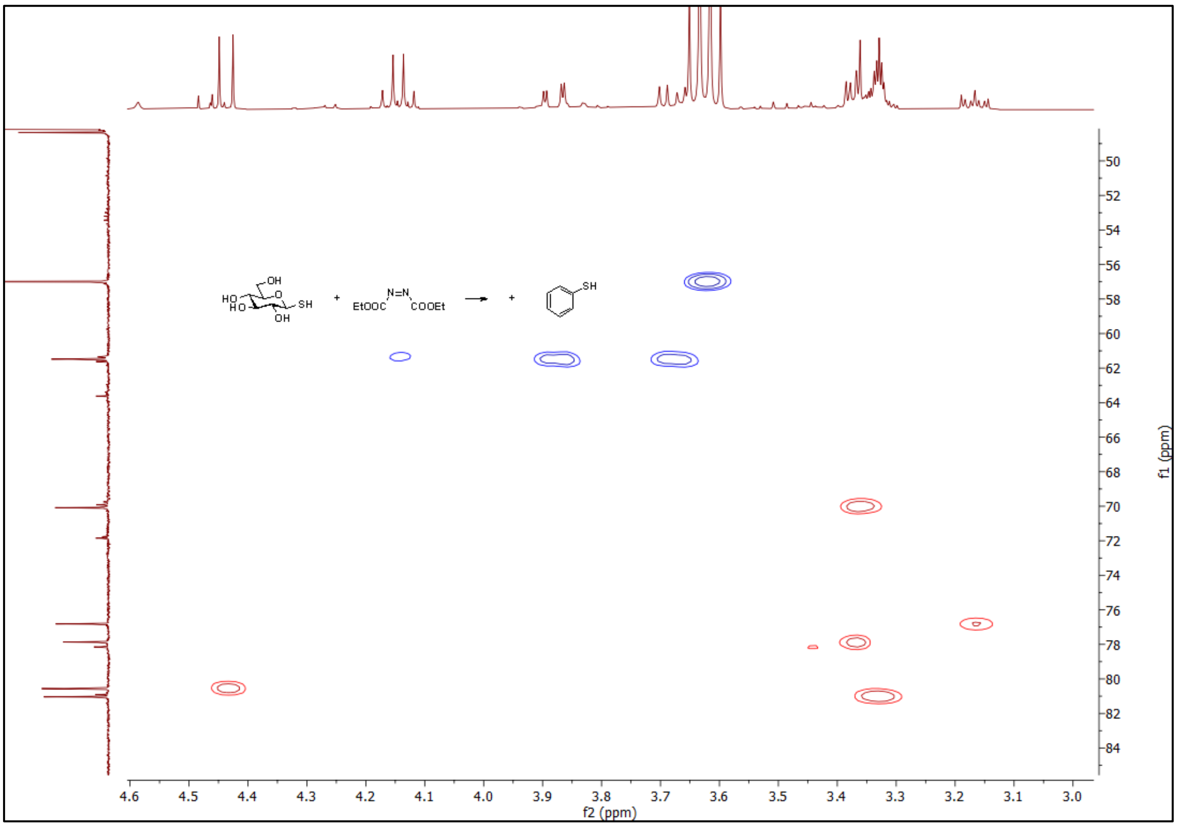

$^{13}C\{^{1}H\}$ NMR of thioglucose$(OH)_4$ plus DEAD followed by thiophenol (1h) (100 MHz, CD_3OD)

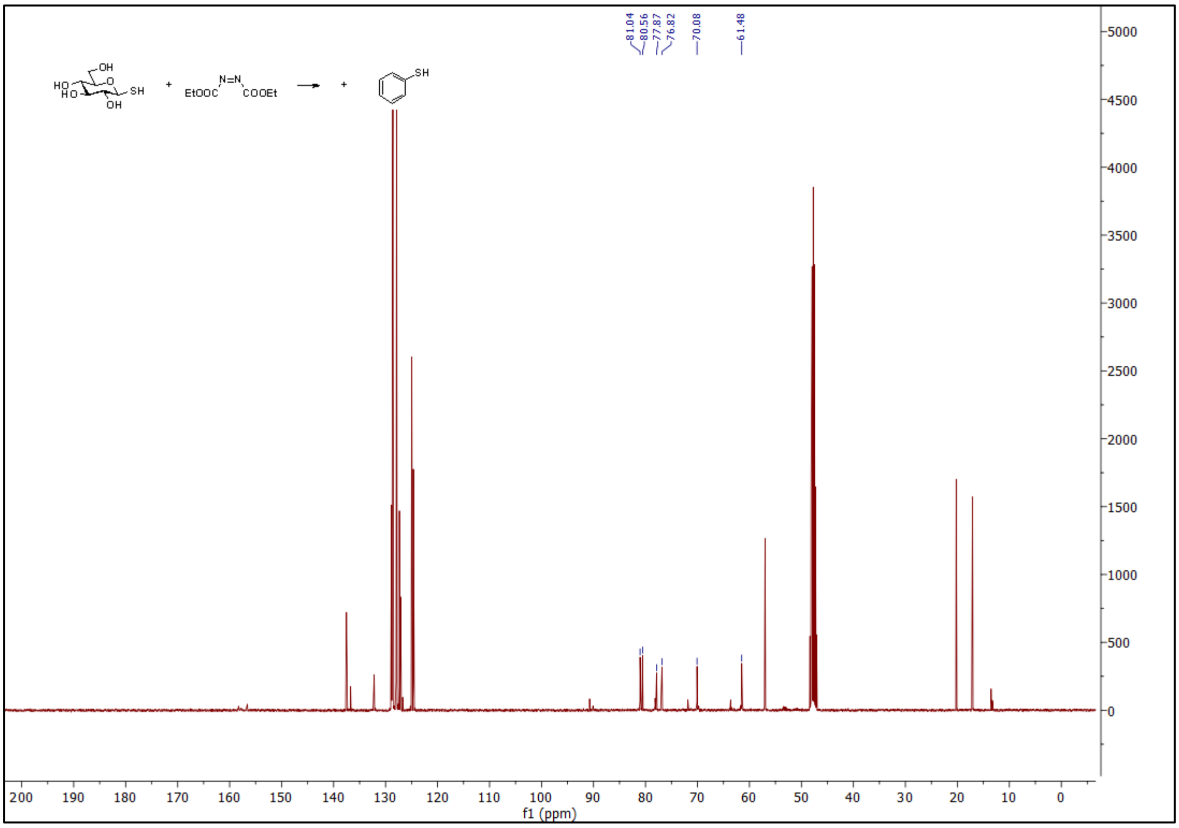

^{1}H NMR of compound **17** (400 MHz, CD_3OD)

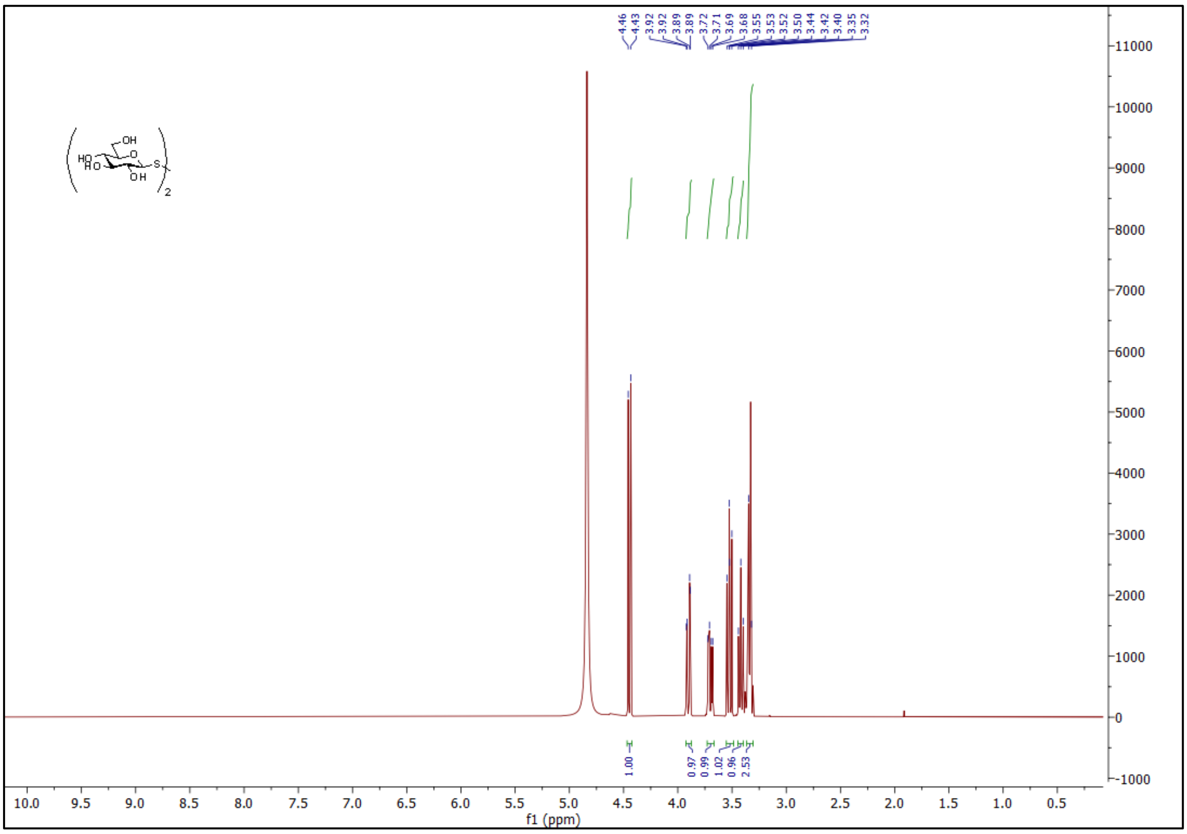

COSY NMR of compound **17**

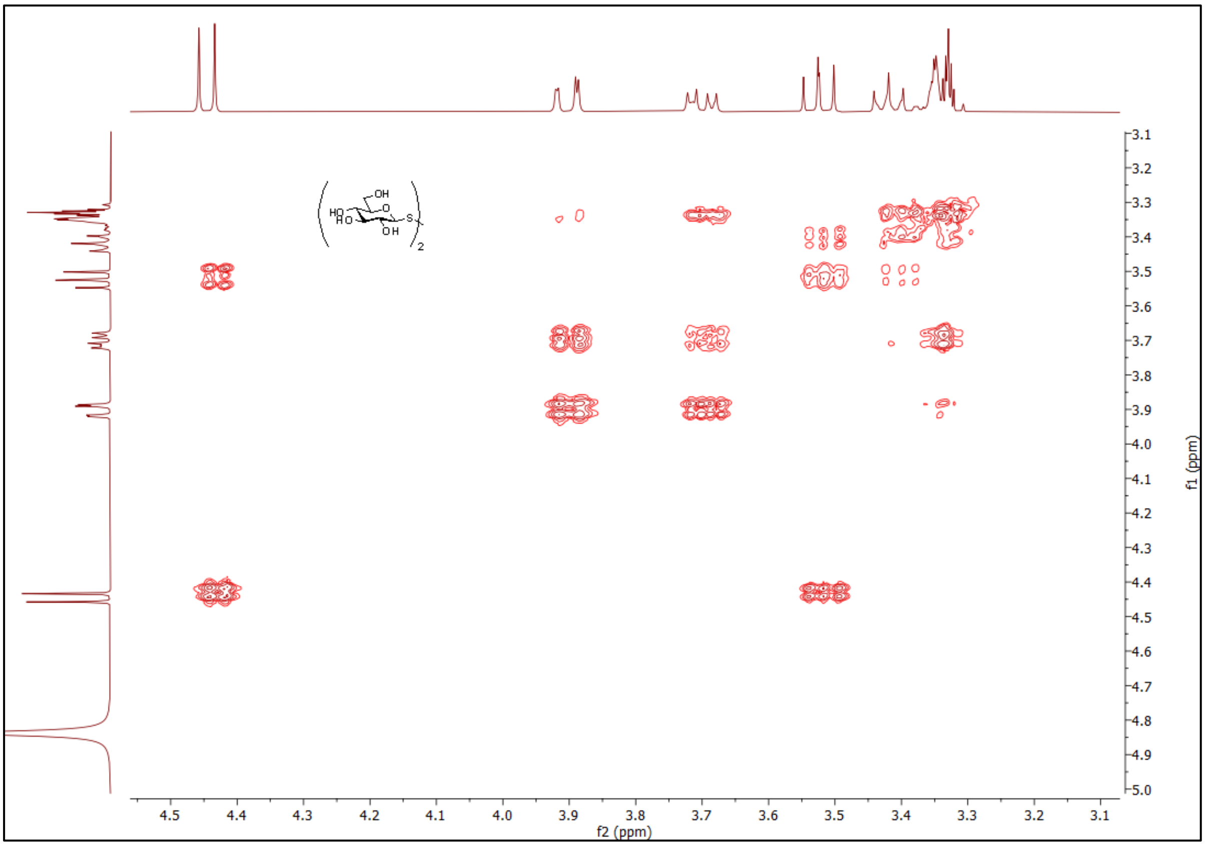

$^{13}C\{^{1}H\}$ NMR of compound **17** (100 MHz, CD_3OD)

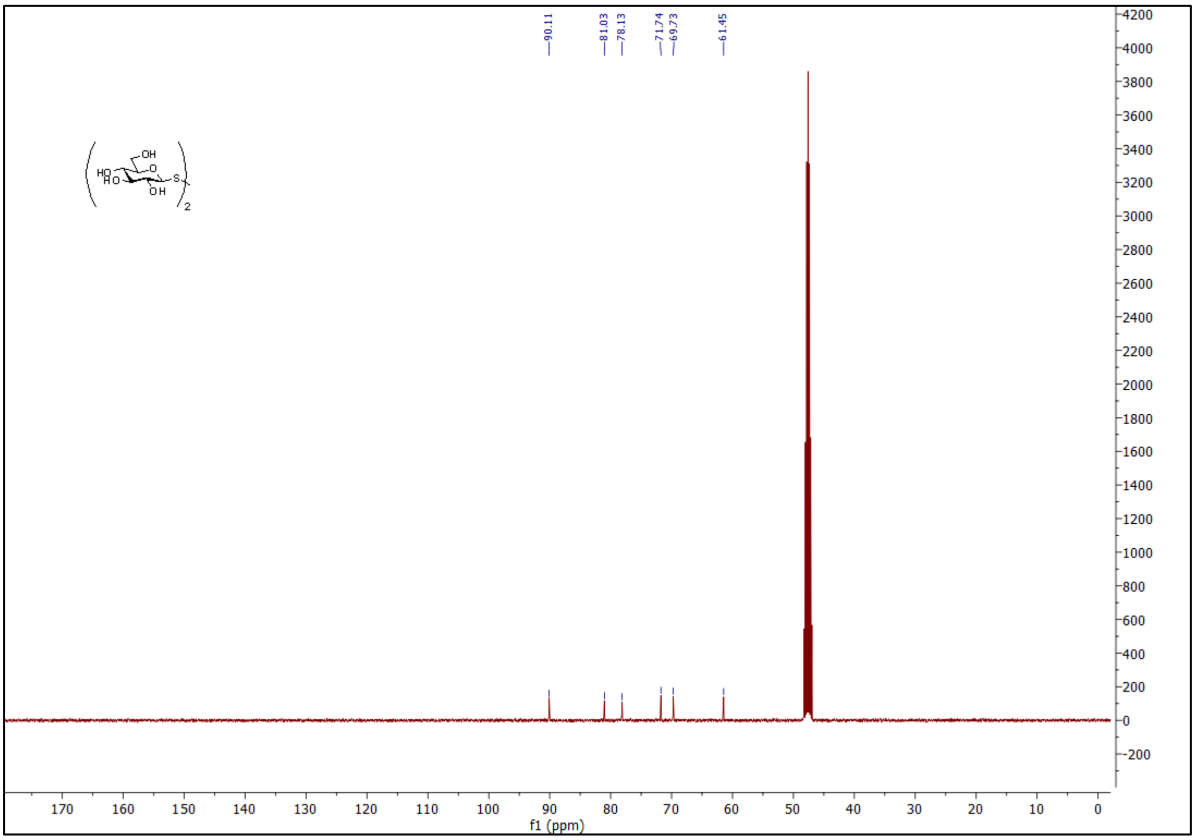

^{1}H NMR of compound **18** (400 MHz, $CDCl_3$)

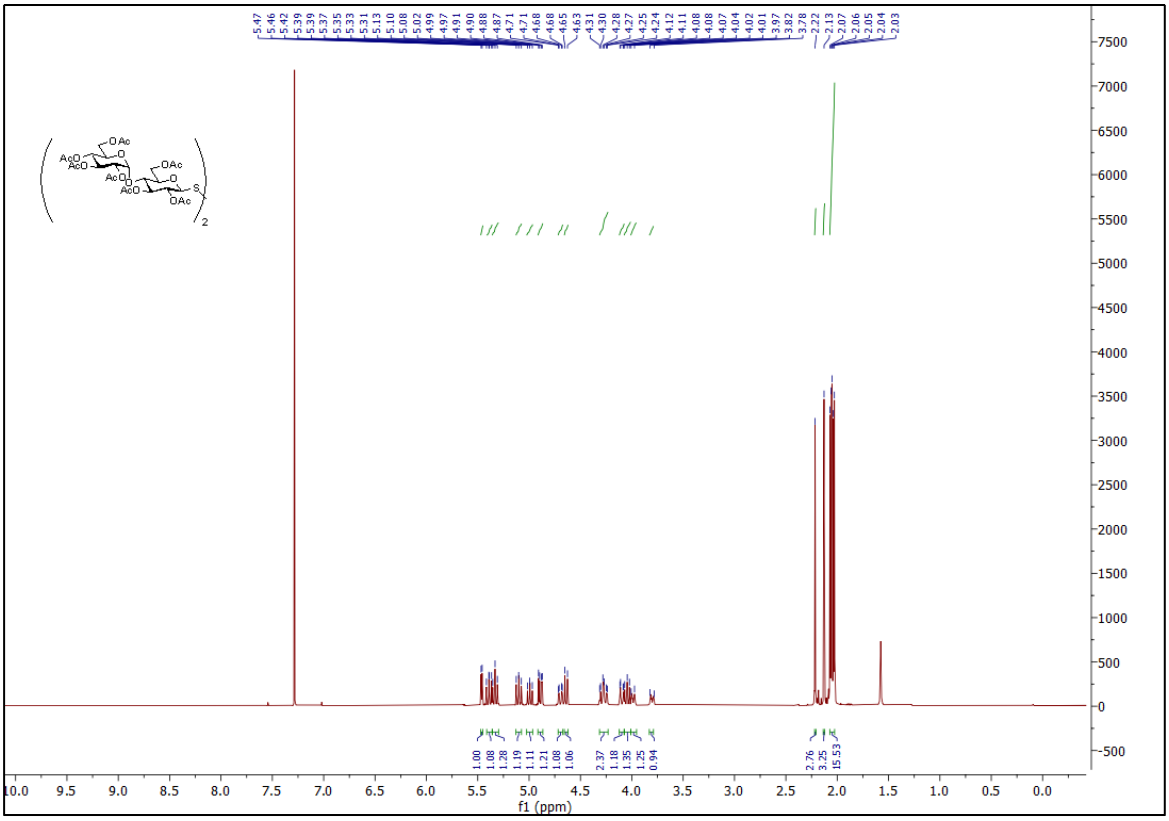

COSY NMR of compound **18**

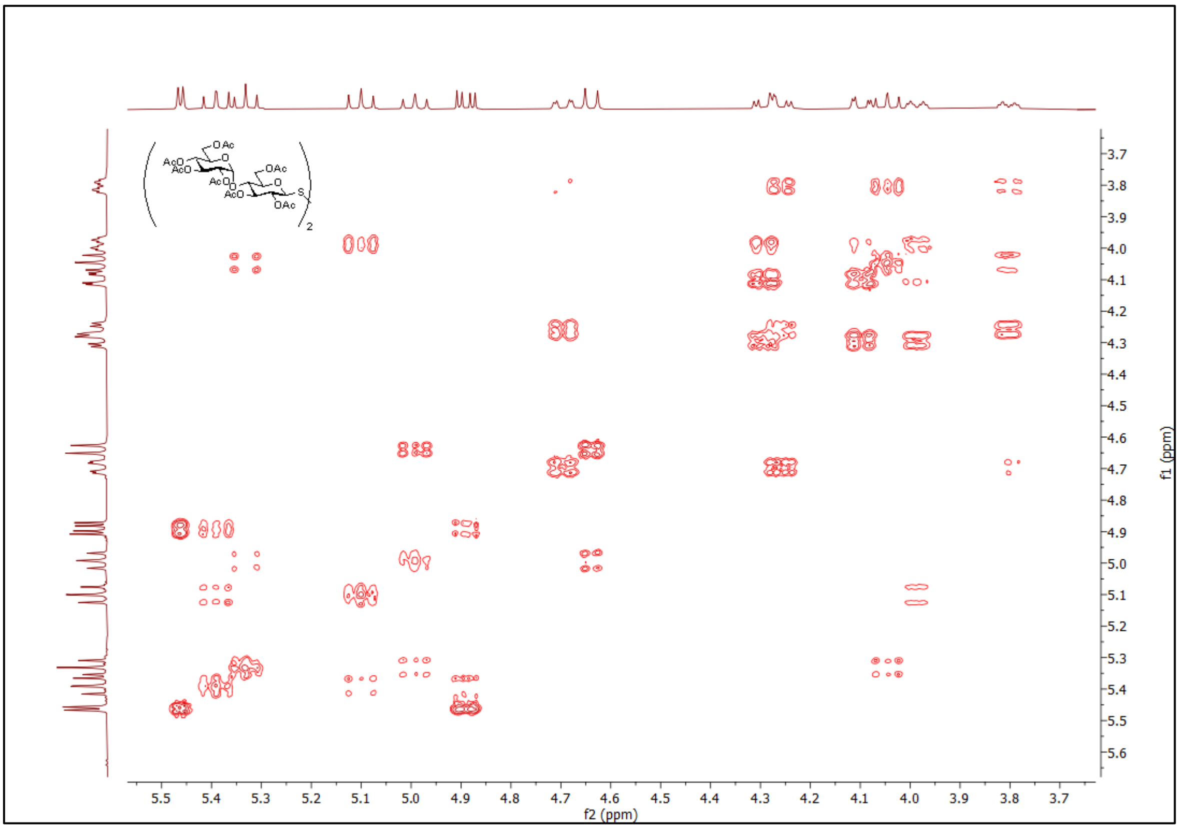

$^{13}C\{^{1}H\}$ NMR of compound **18** (100 MHz, $CDCl_3$)

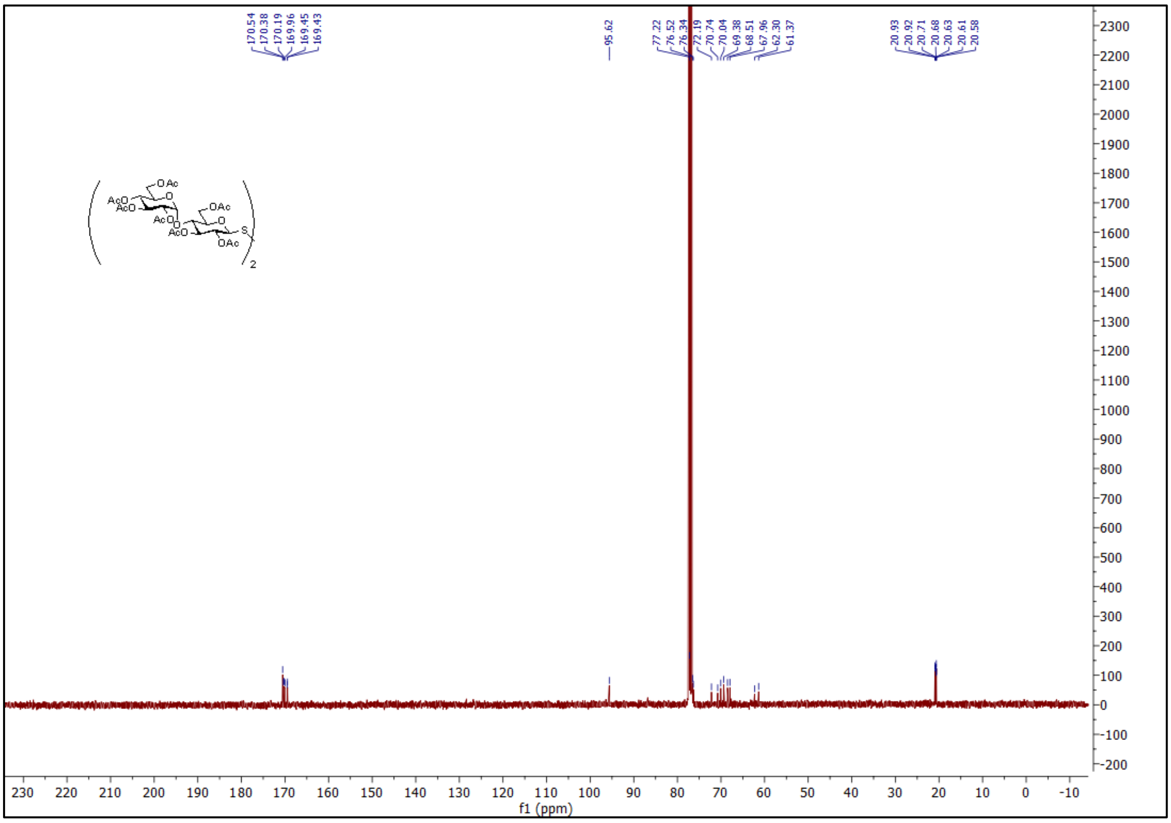

^{1}H NMR of compound **19** (400 MHz, D_2O)

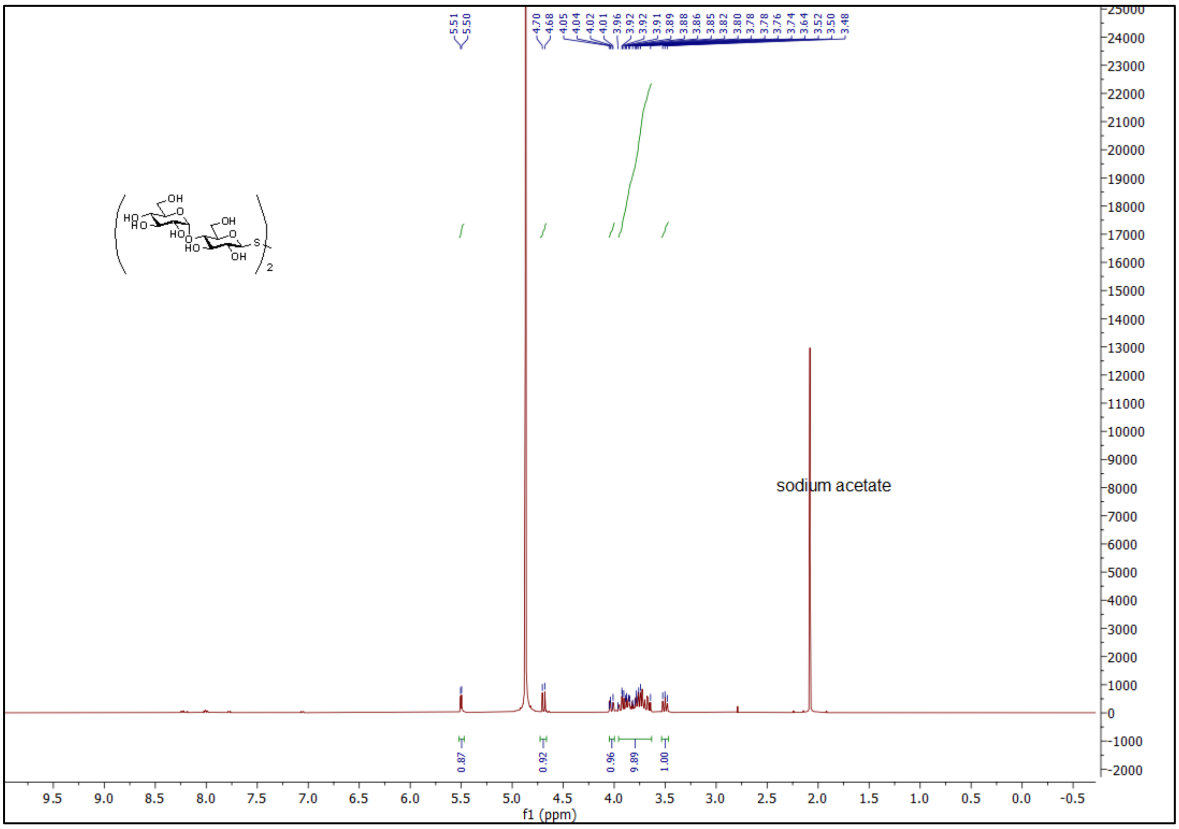

COSY NMR of compound **19**

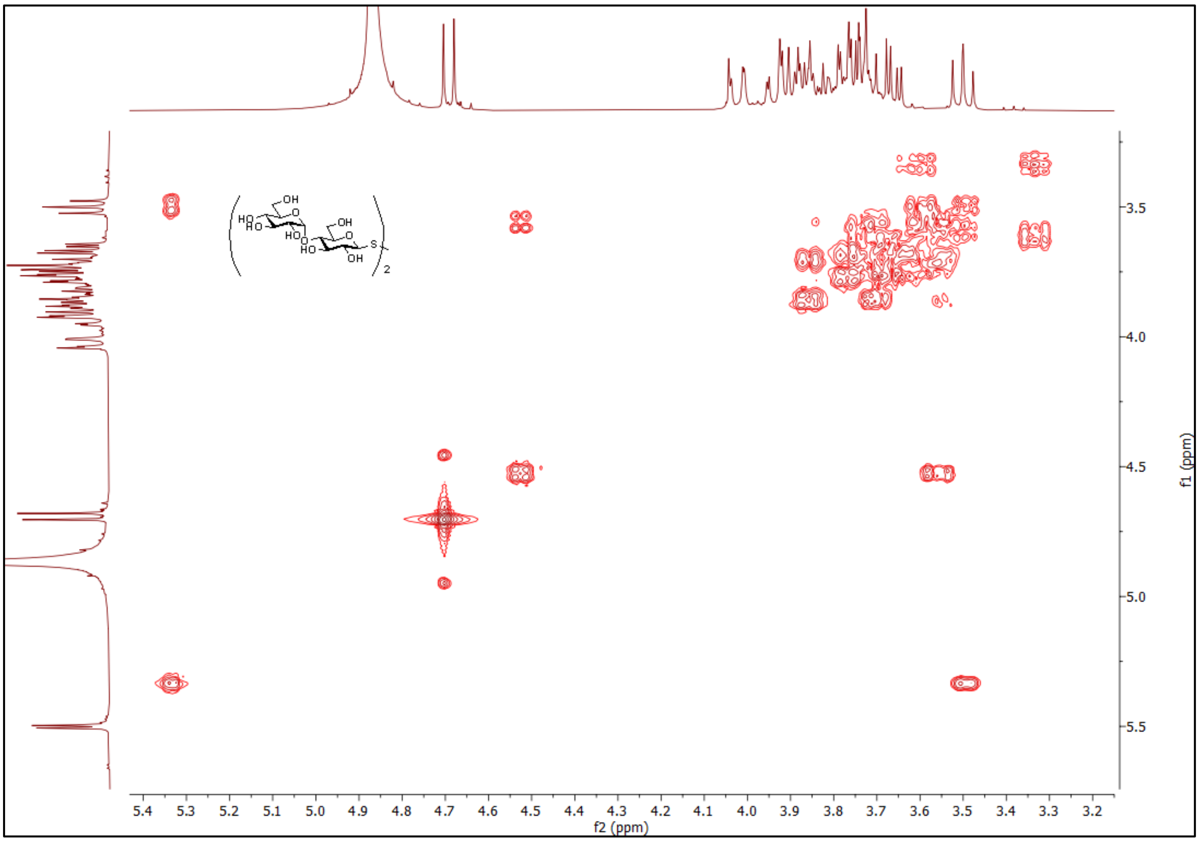

$^{13}C\{^{1}H\}$ NMR of compound **19** (100 MHz, D_2O)

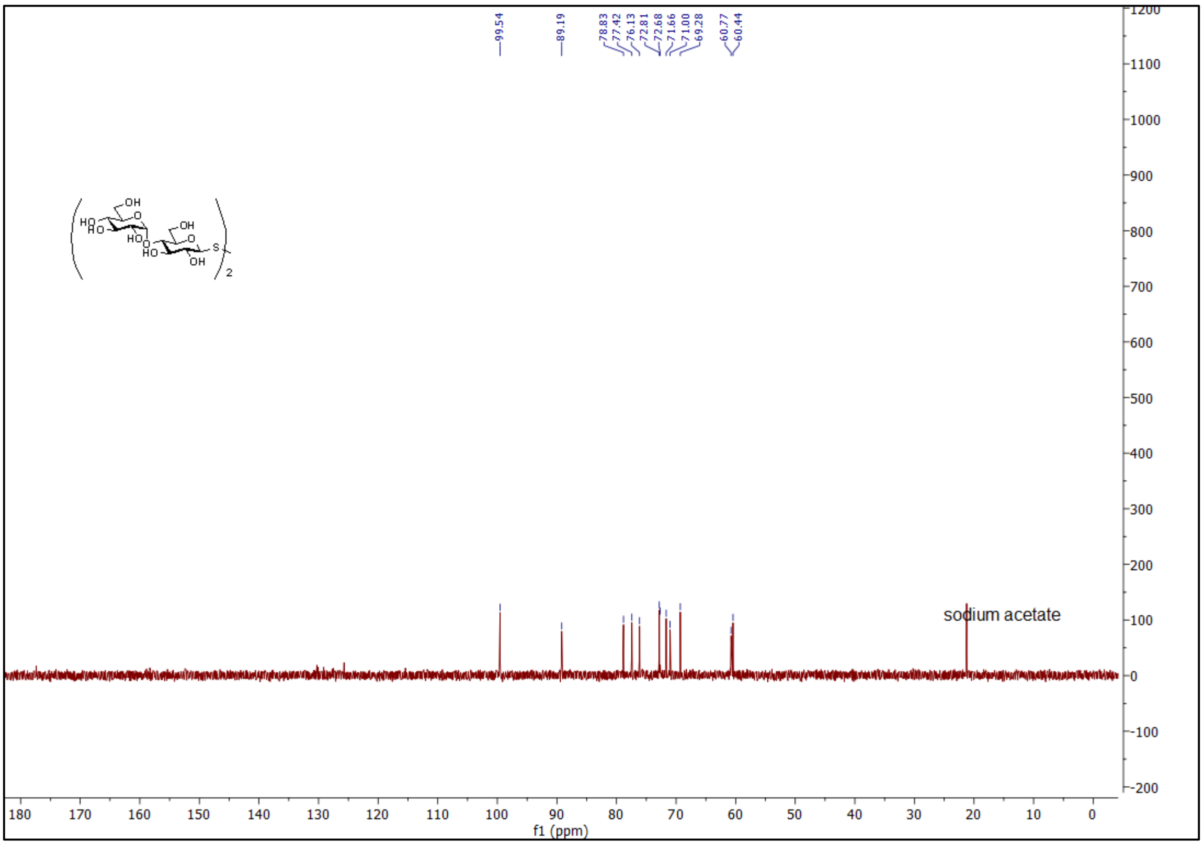

^{1}H NMR of compound **20** (400 MHz, $CDCl_3$)

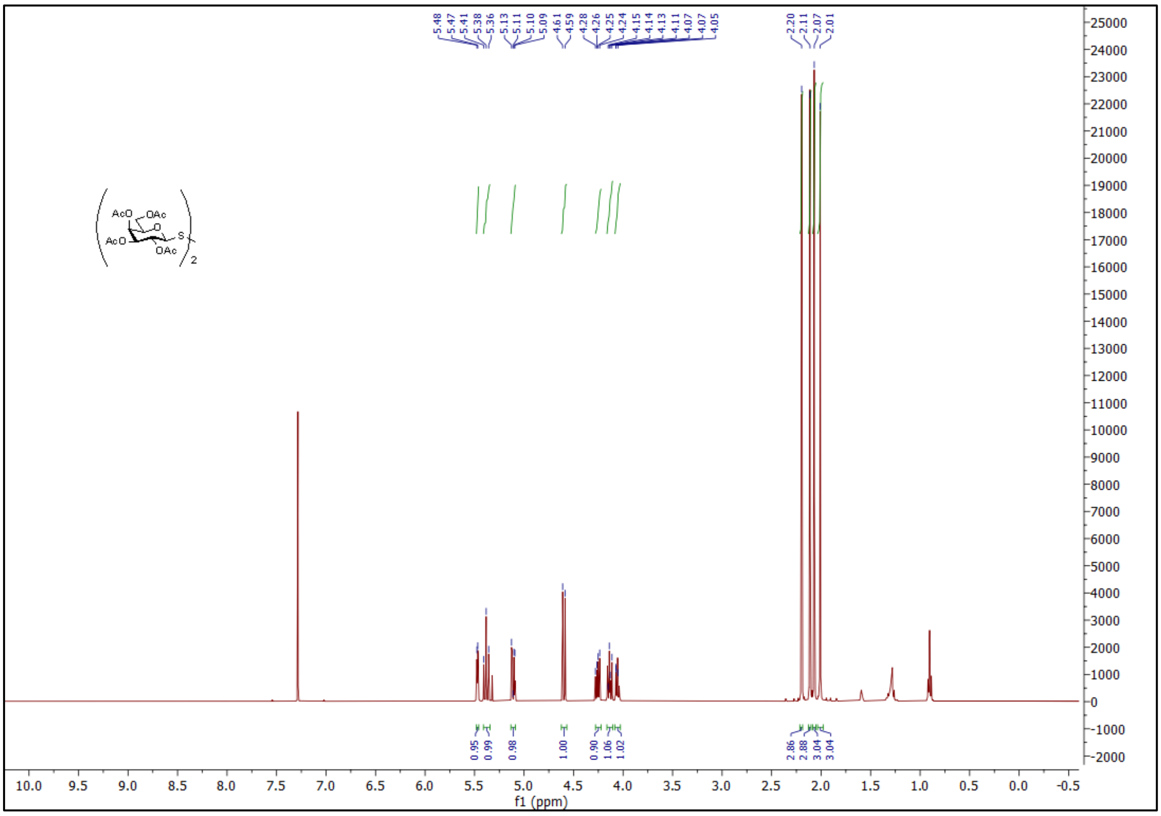

COSY of compound **20**

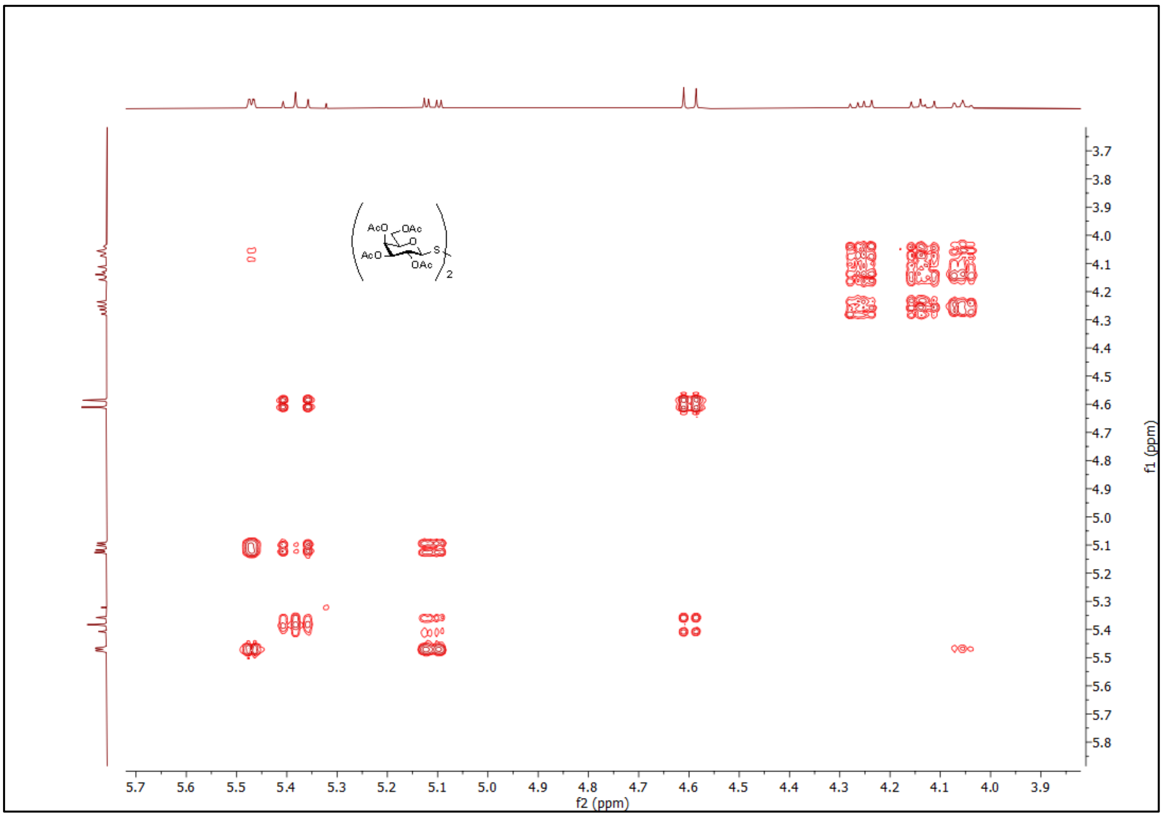

$^{13}C\{^{1}H\}$ NMR of compound **20** (100 MHz, $CDCl_3$)

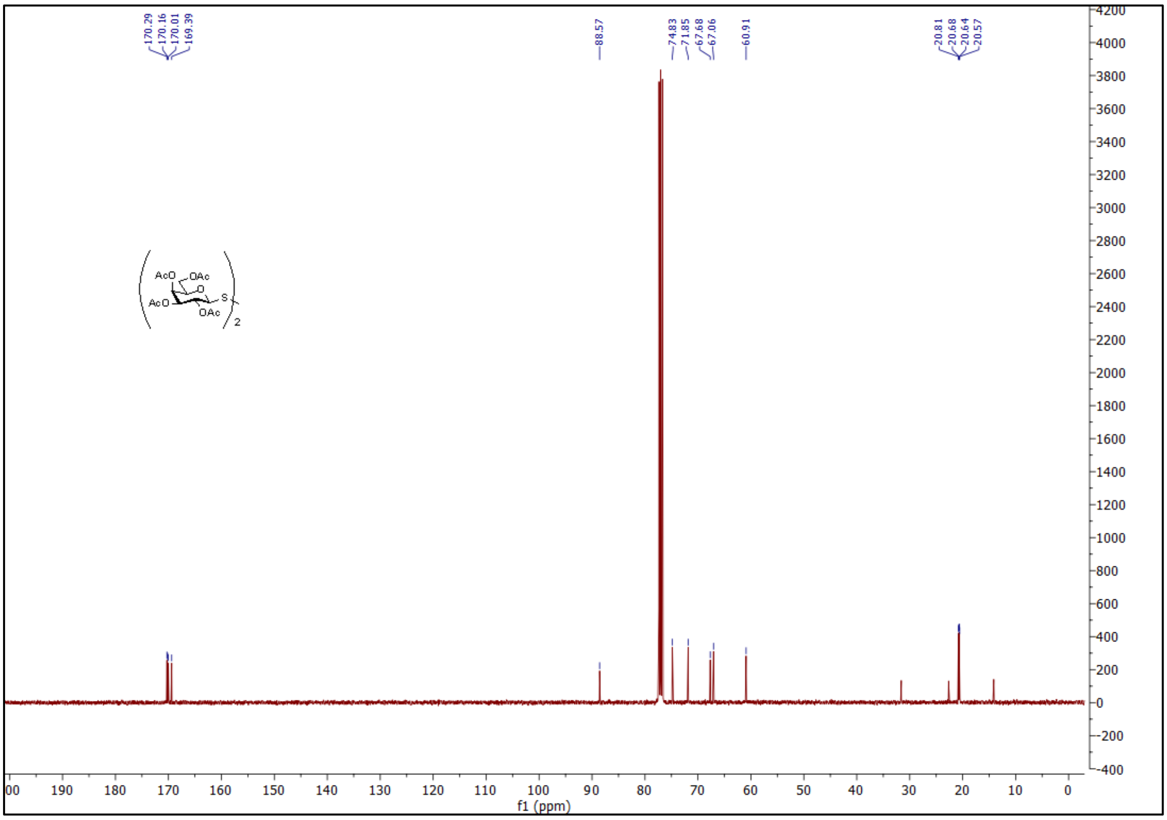

^{1}H NMR of compound **21** (400 MHz, D_2O)

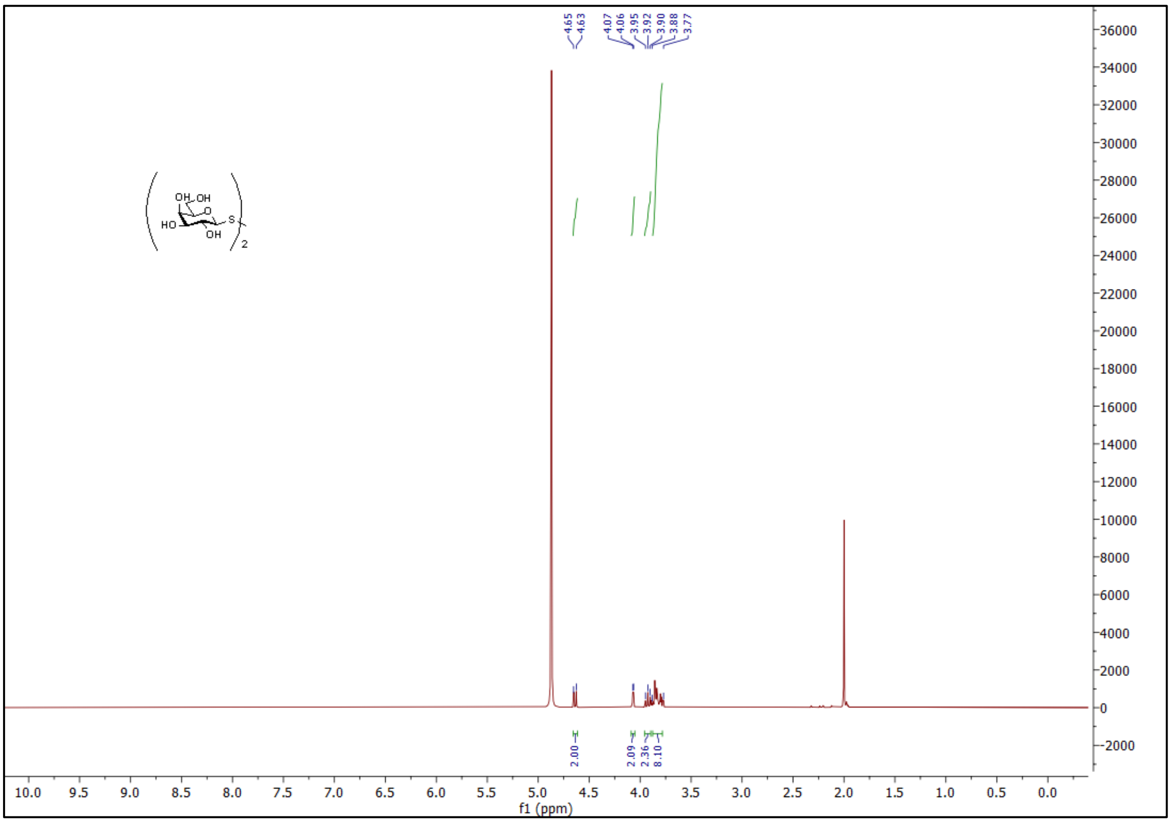

COSY NMR of compound **21**

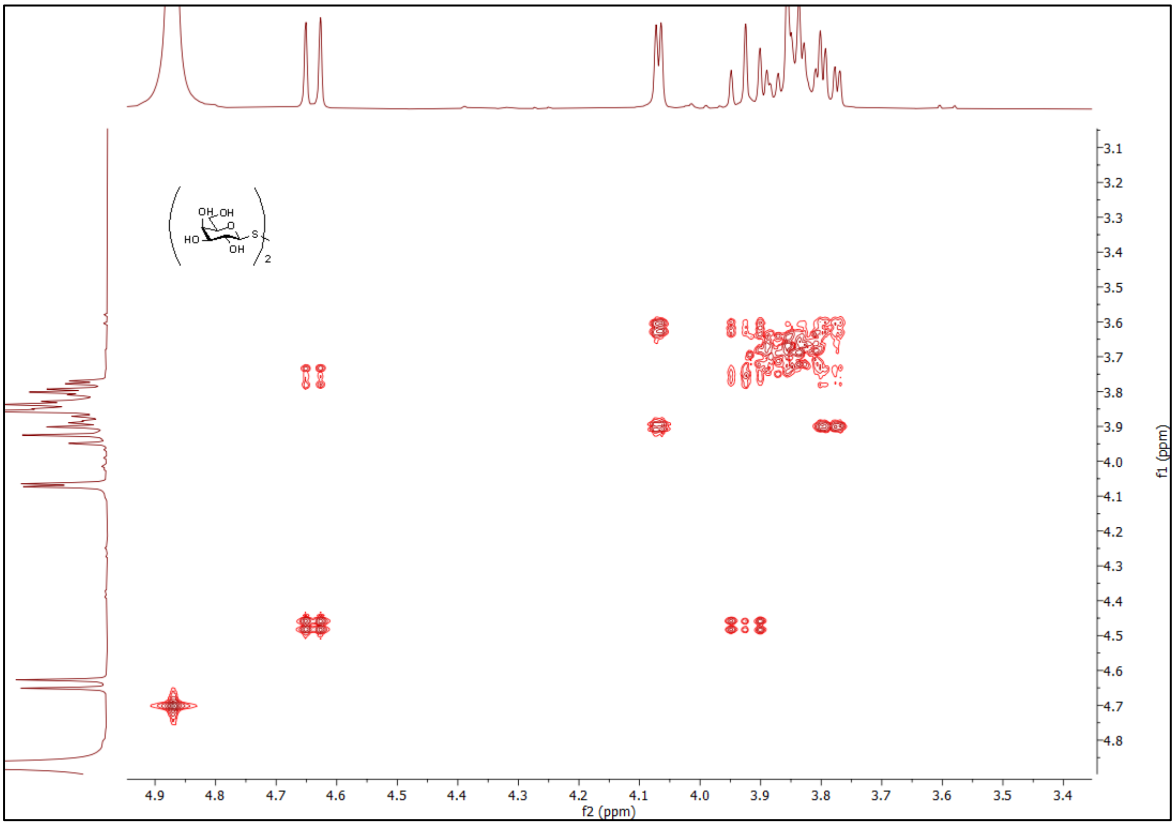

$^{13}C\{^1H\}$ NMR of compound **21** (100 MHz, D_2O)

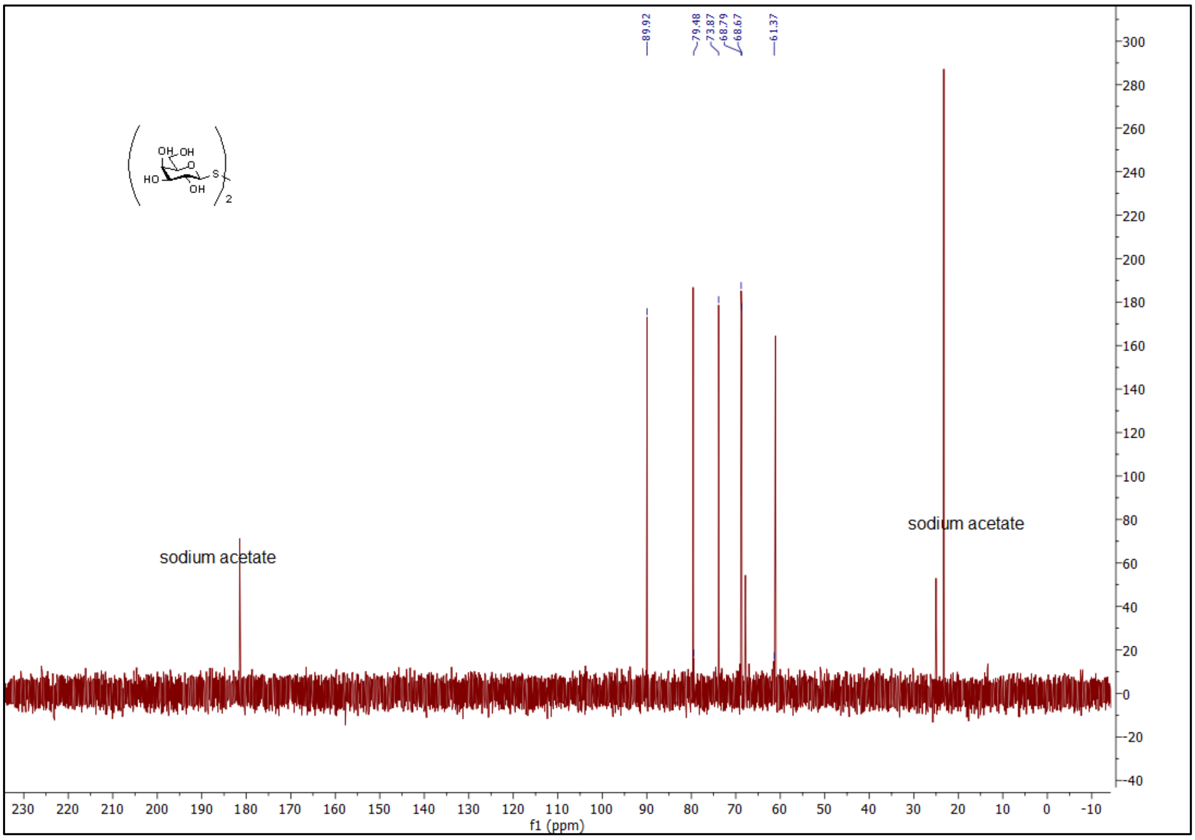

^{1}H NMR of compound **22** (400 MHz, $CDCl_3$)

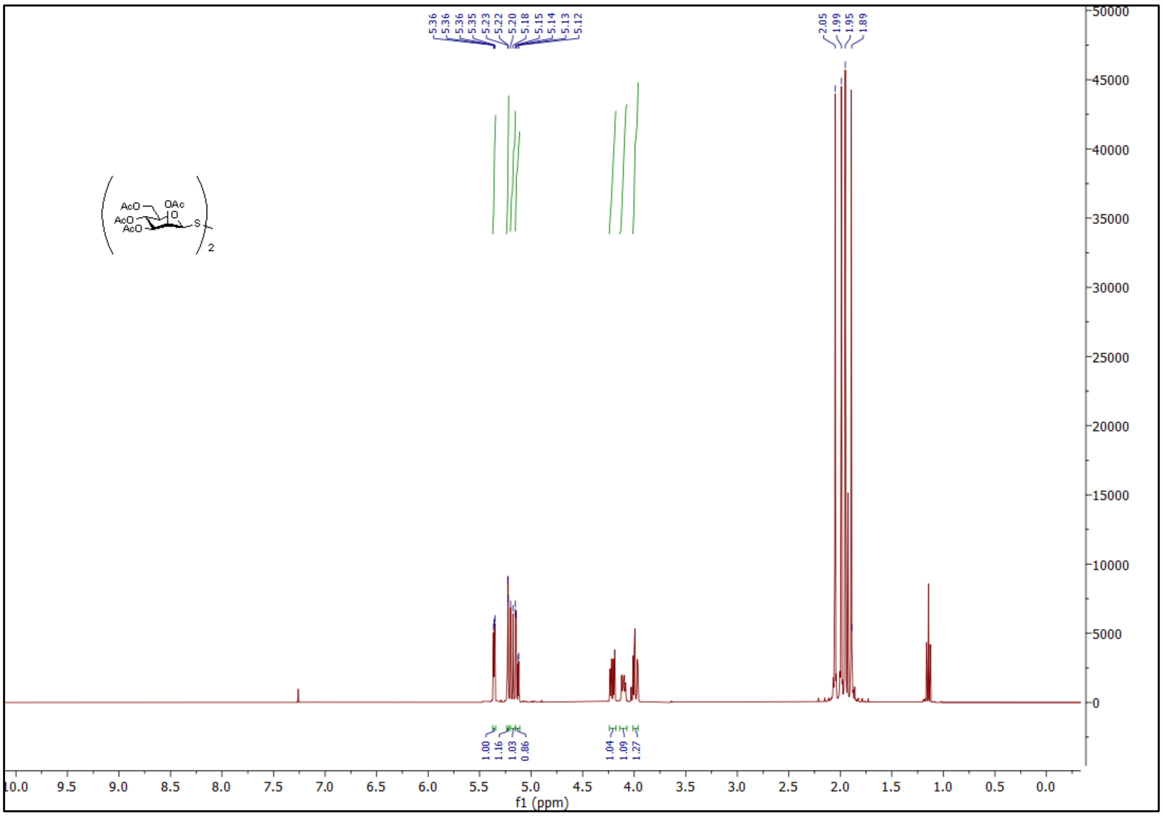

COSY NMR of compound **22**

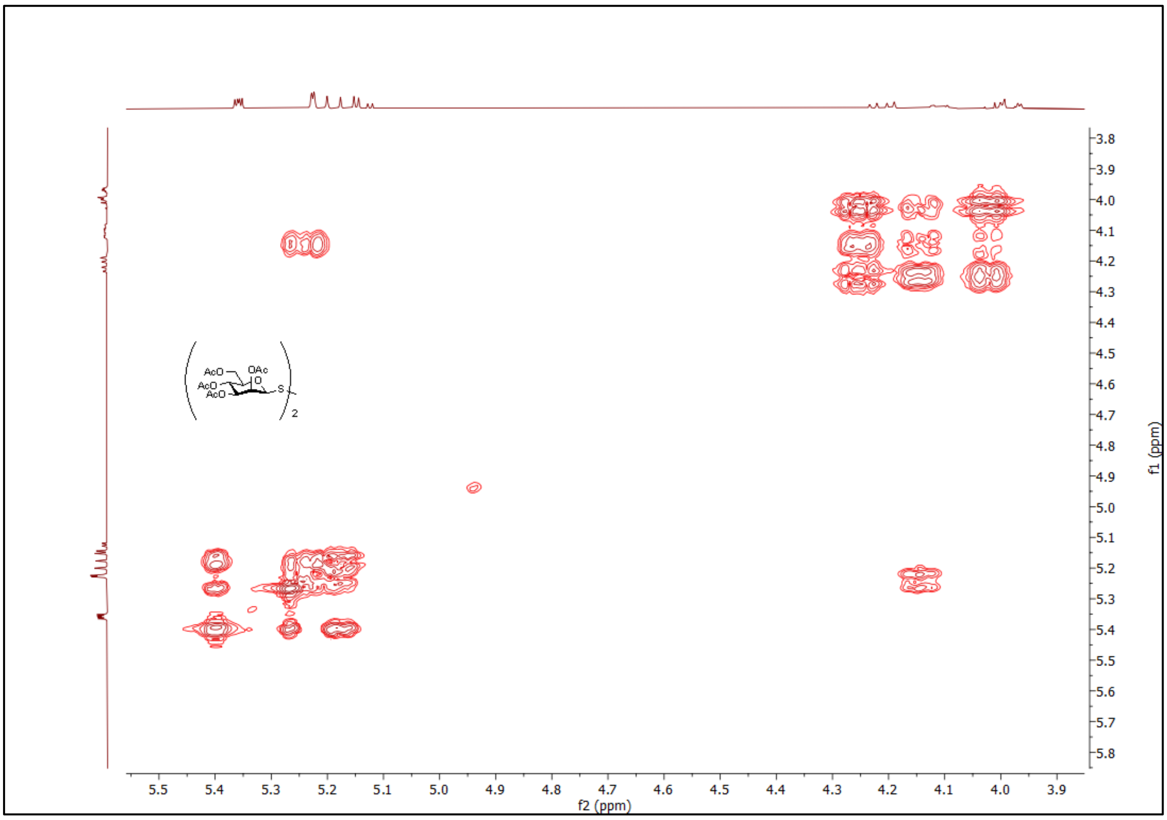

$^{13}C\{^{1}H\}$ NMR of compound **22** (100 MHz, $CDCl_3$)

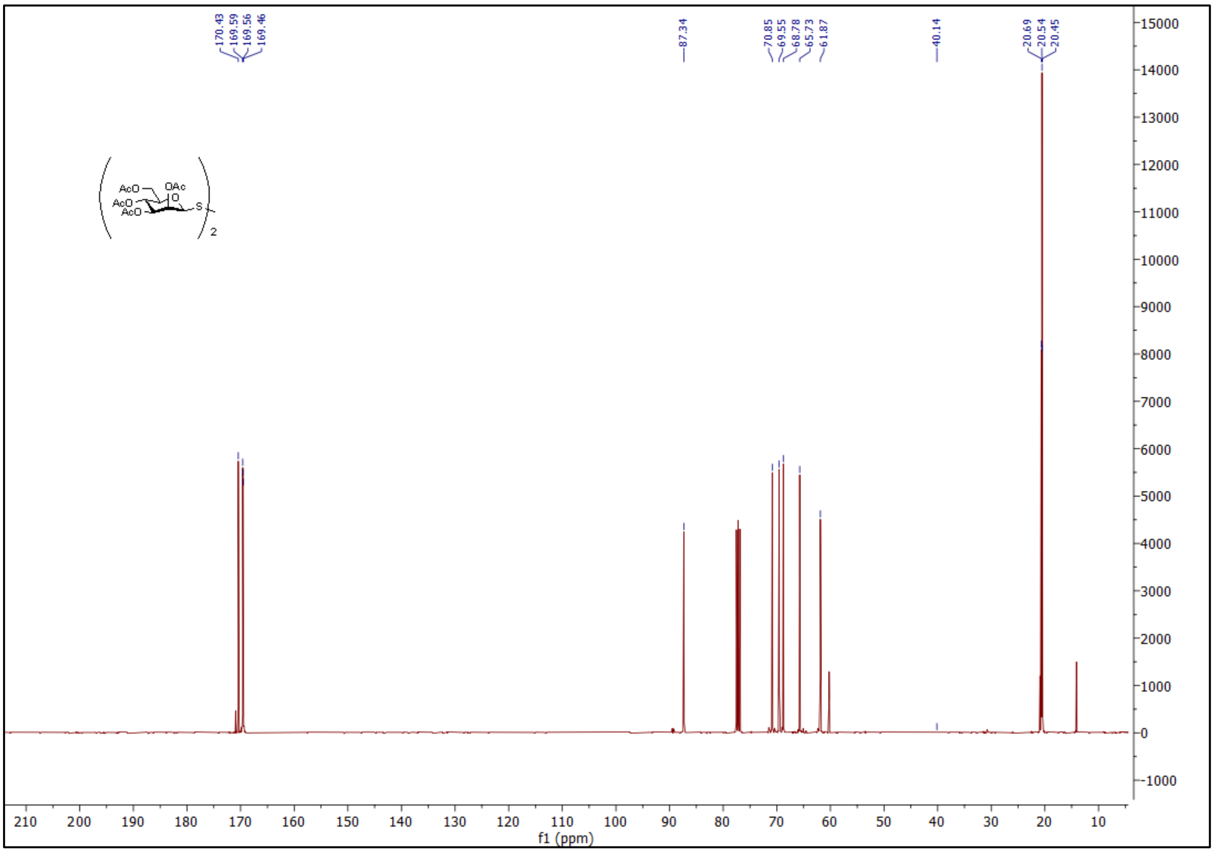

HPLC chromatogram of crude compound **3a** and corresponding mass spectrum

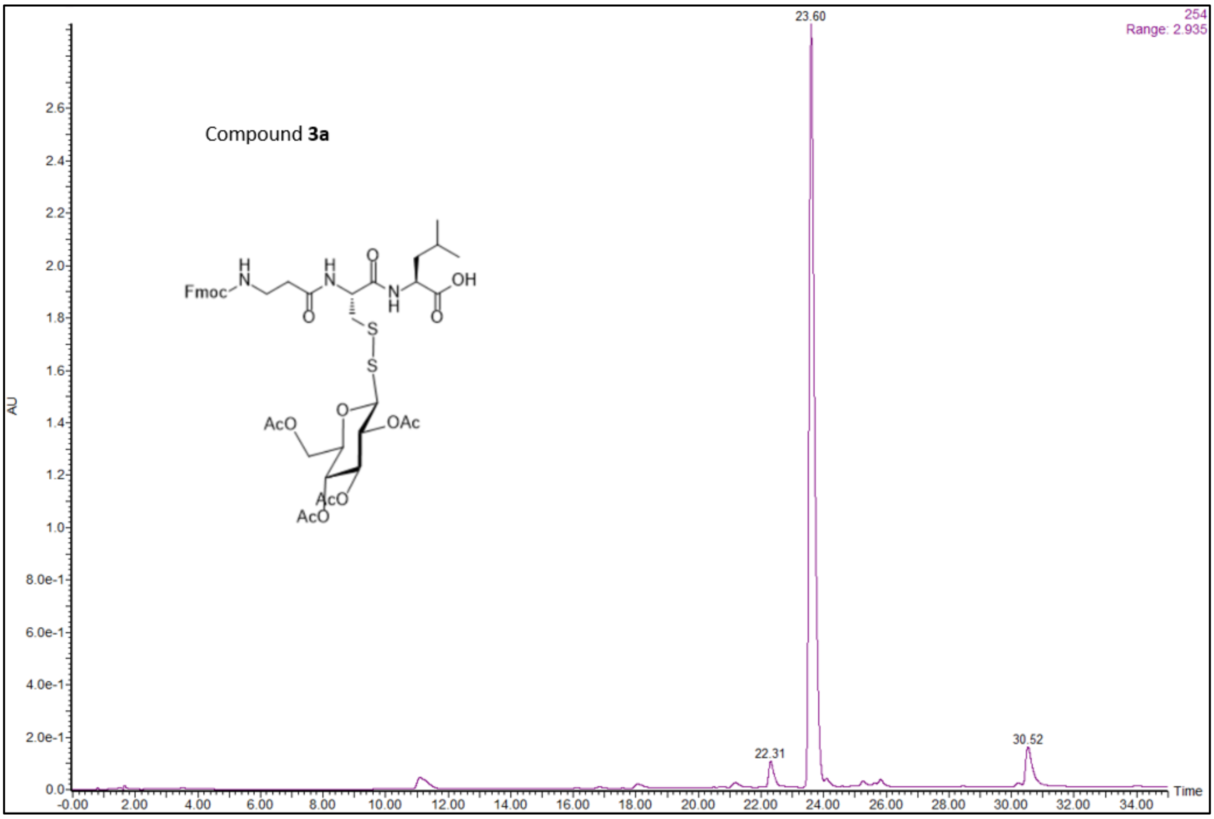

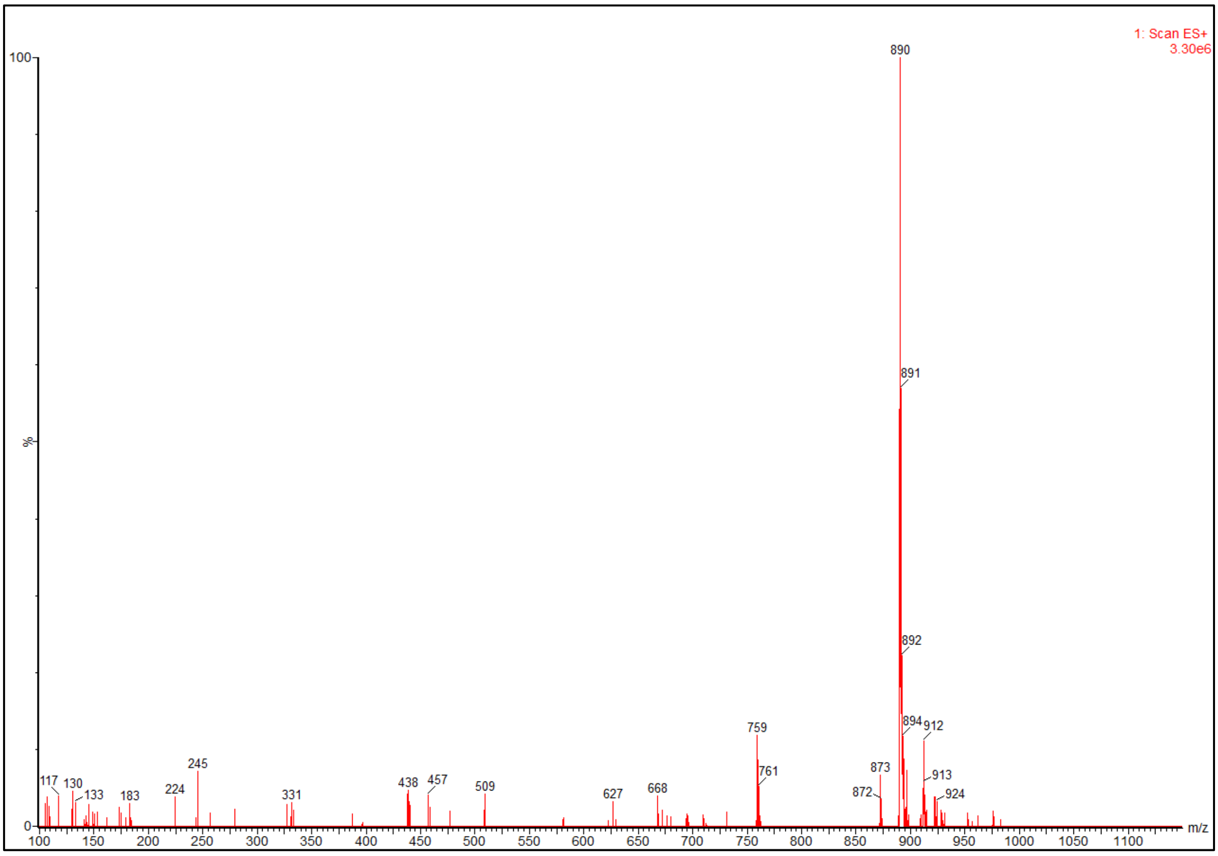

HPLC chromatogram of crude compound **3b** and corresponding mass spectrum

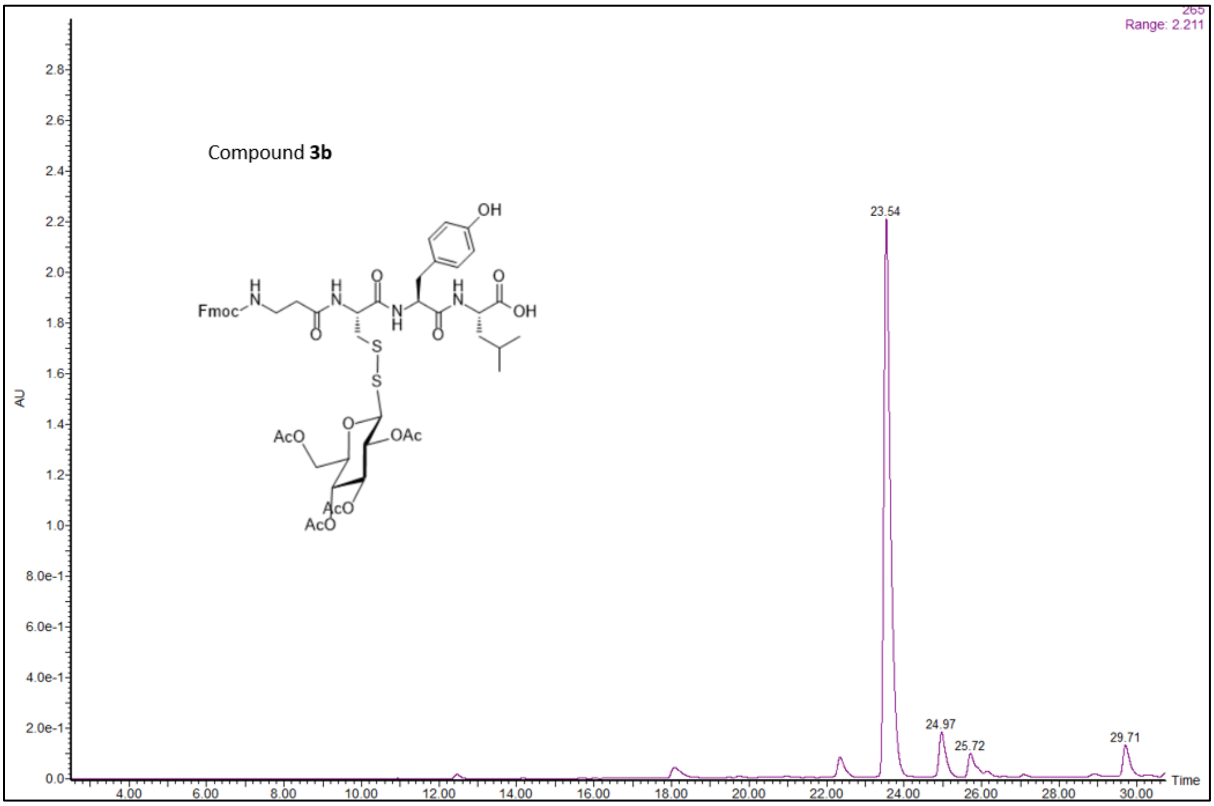

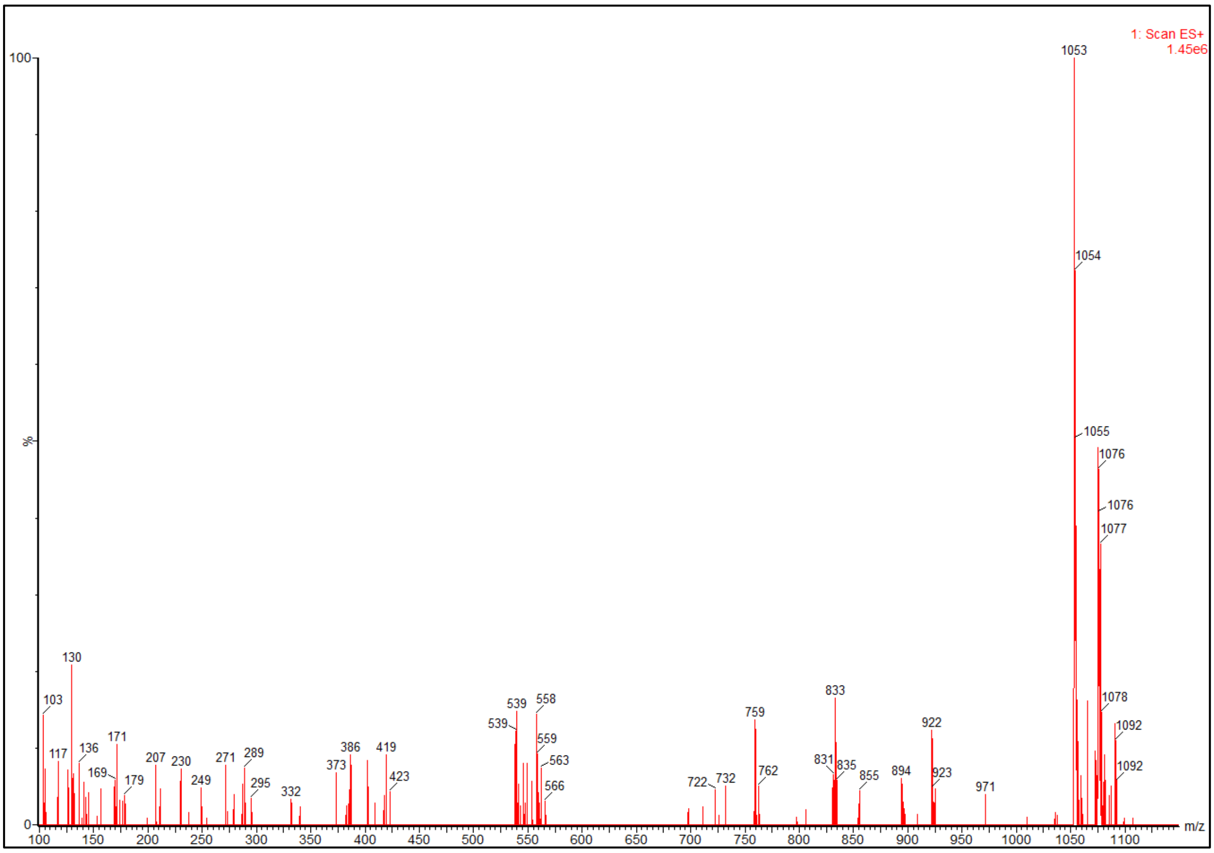

HPLC chromatogram of crude compound **3c** and corresponding mass spectrum

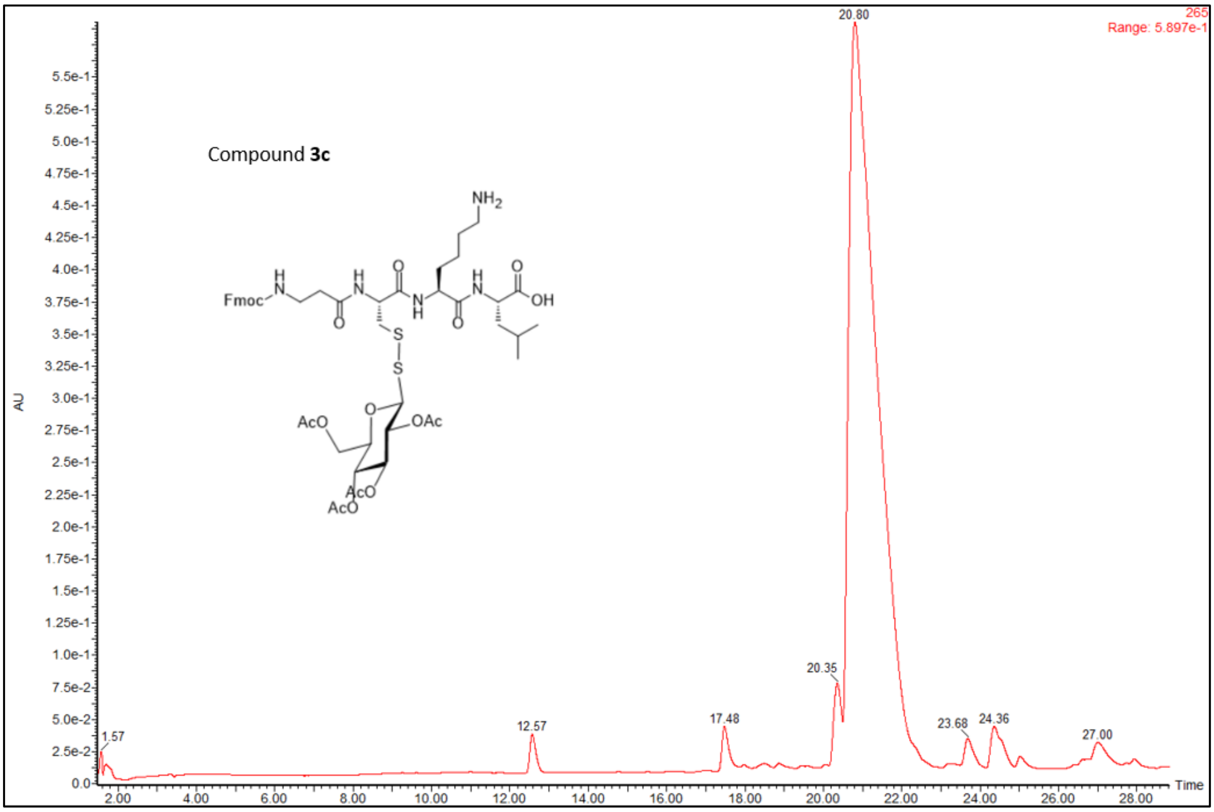

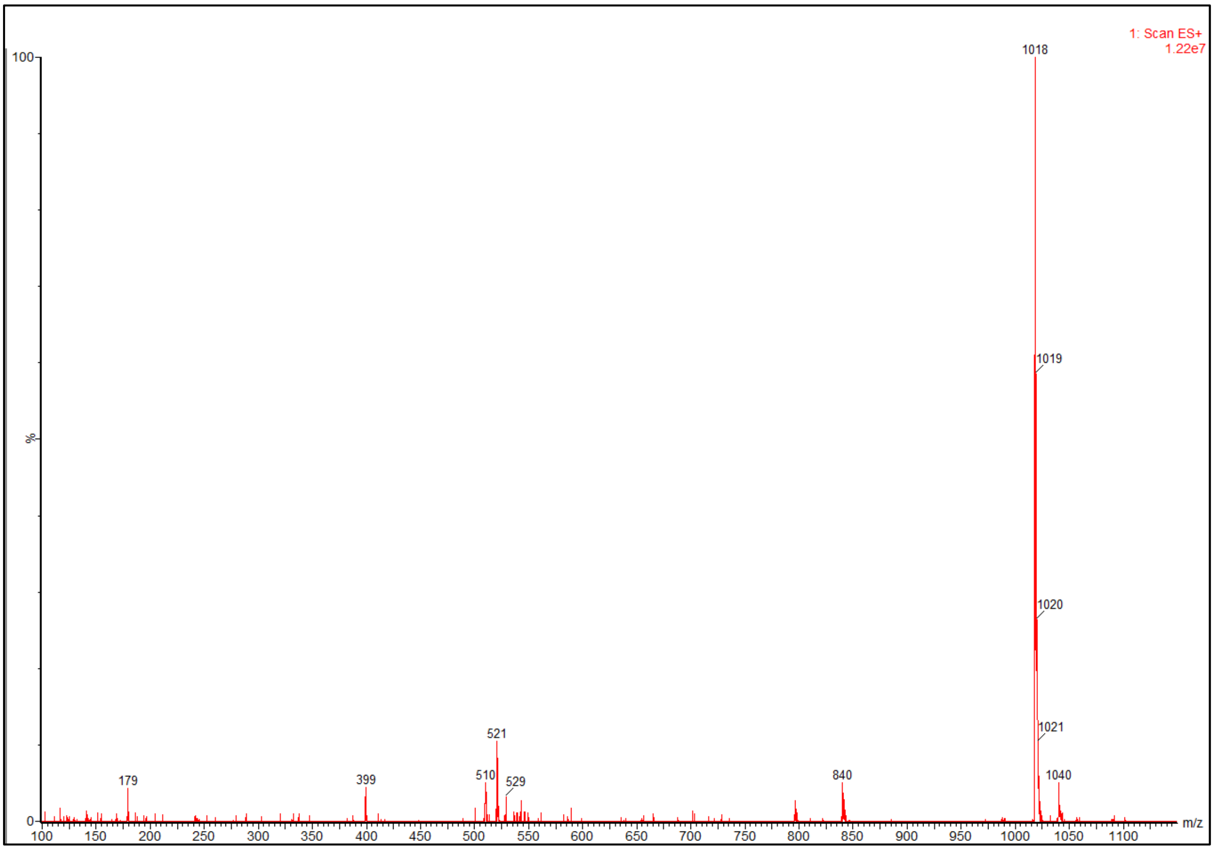

HPLC chromatogram of crude compound **3d** and corresponding mass spectrum

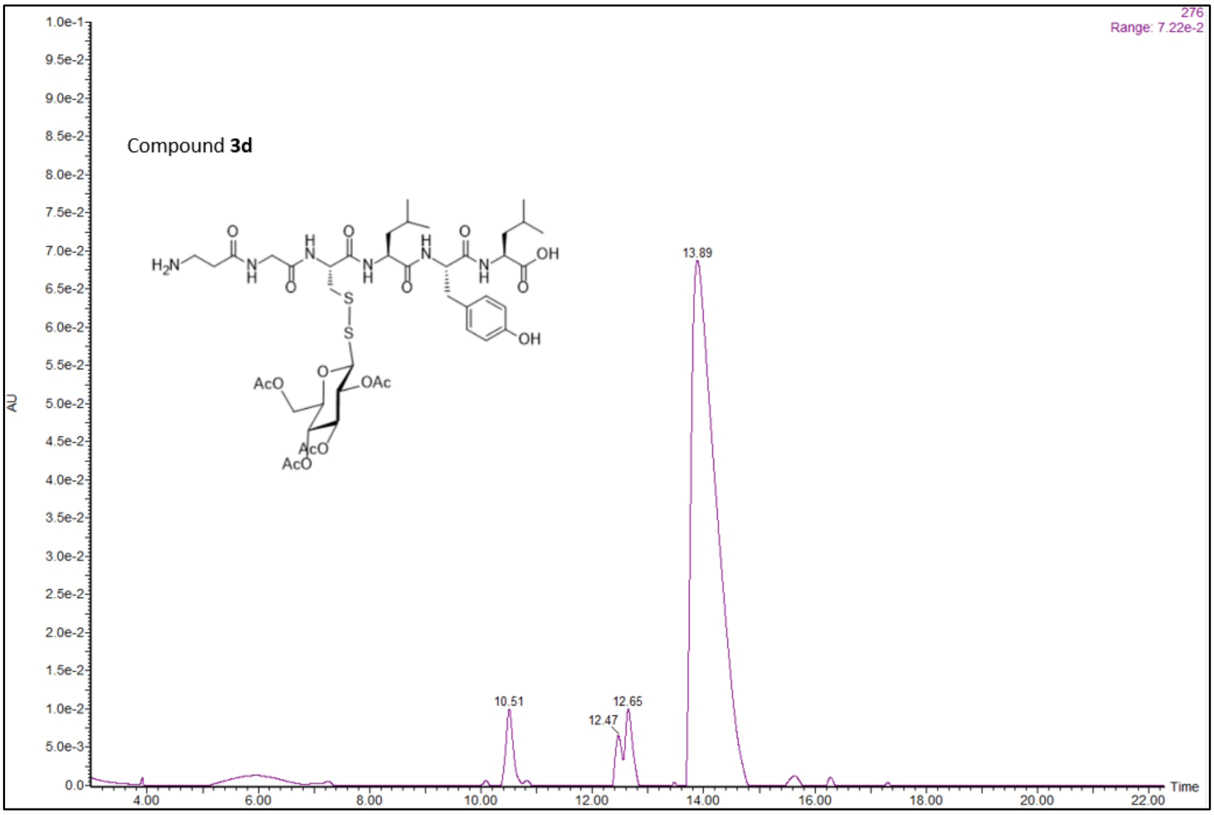

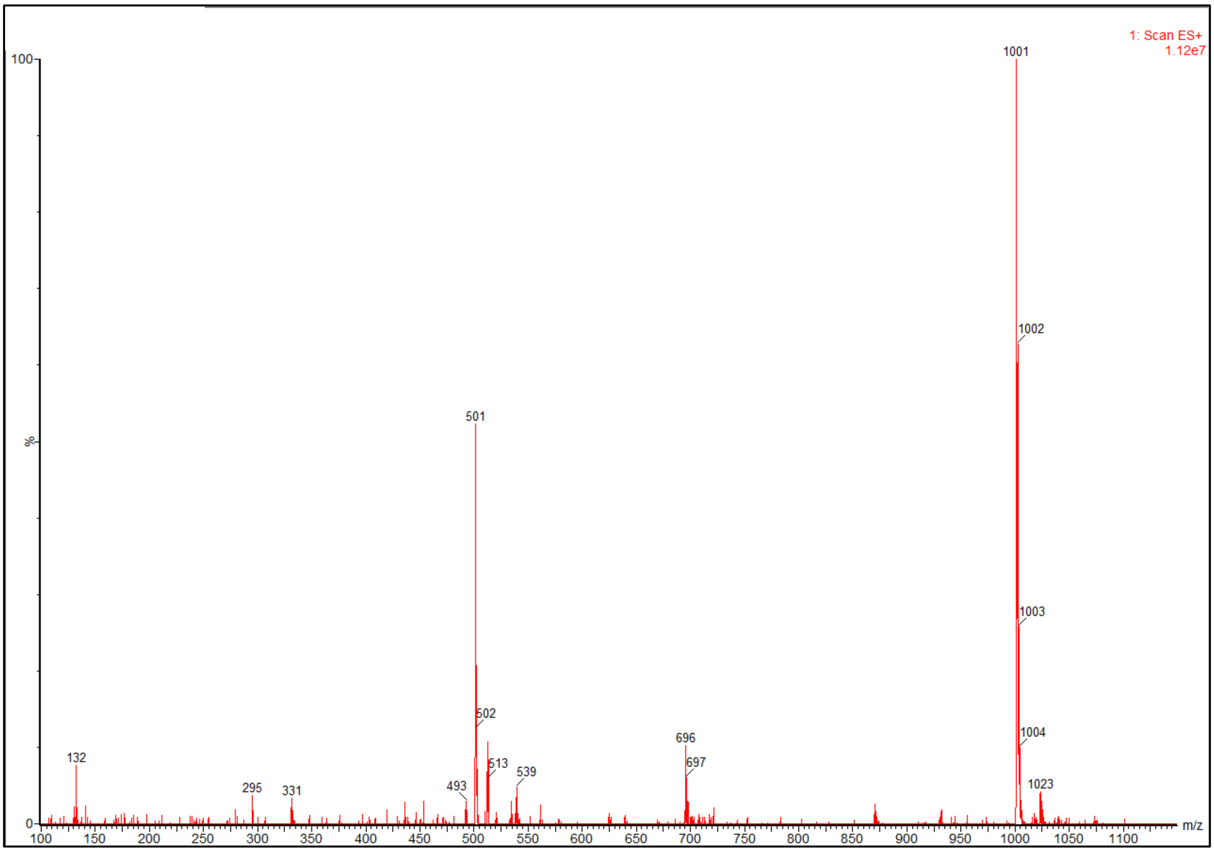

HPLC chromatogram of crude compound **3e** and corresponding mass spectrum

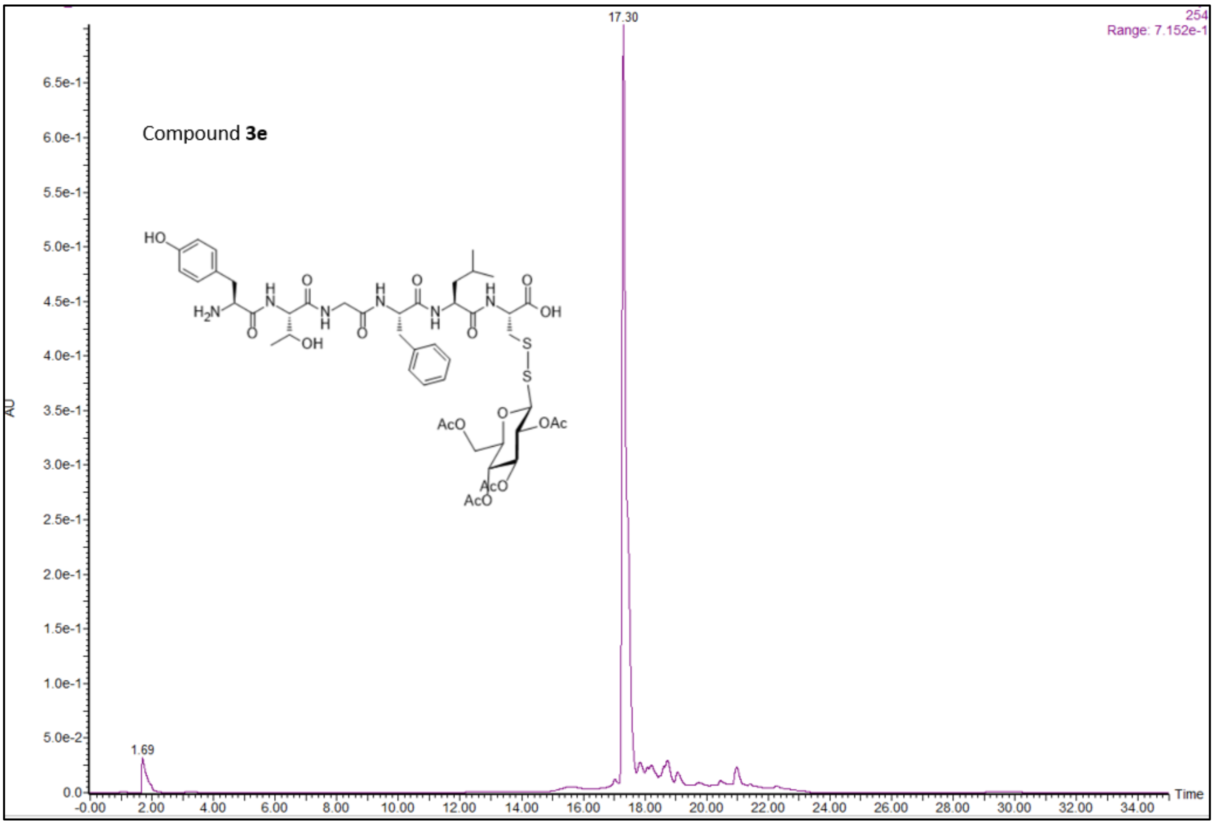

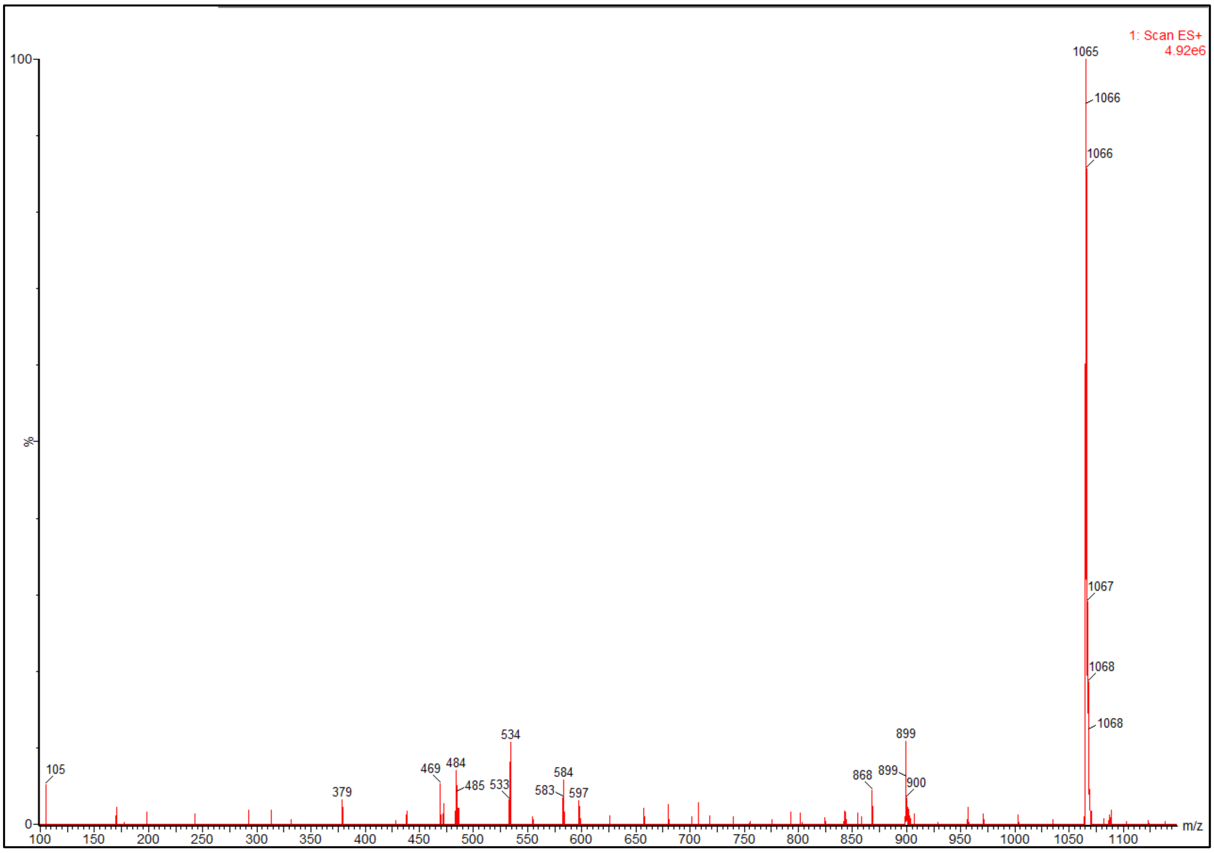

HPLC chromatogram of crude compound **3g** and corresponding mass spectrum

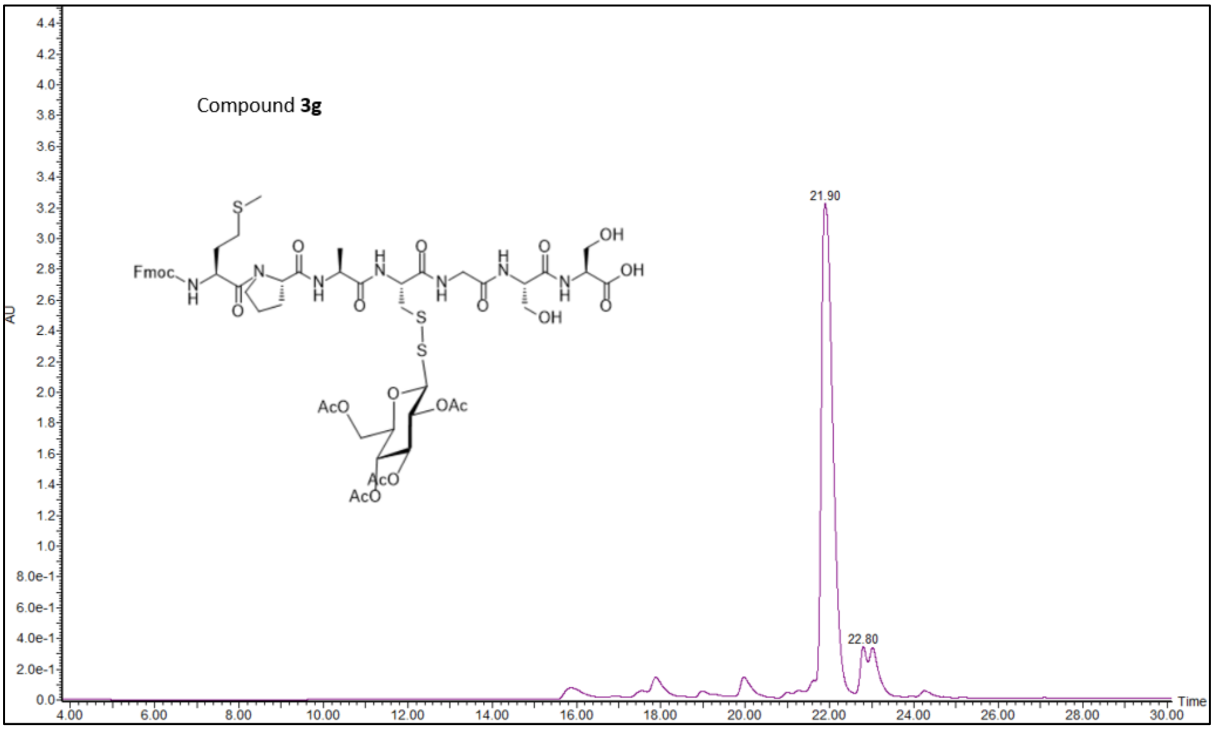

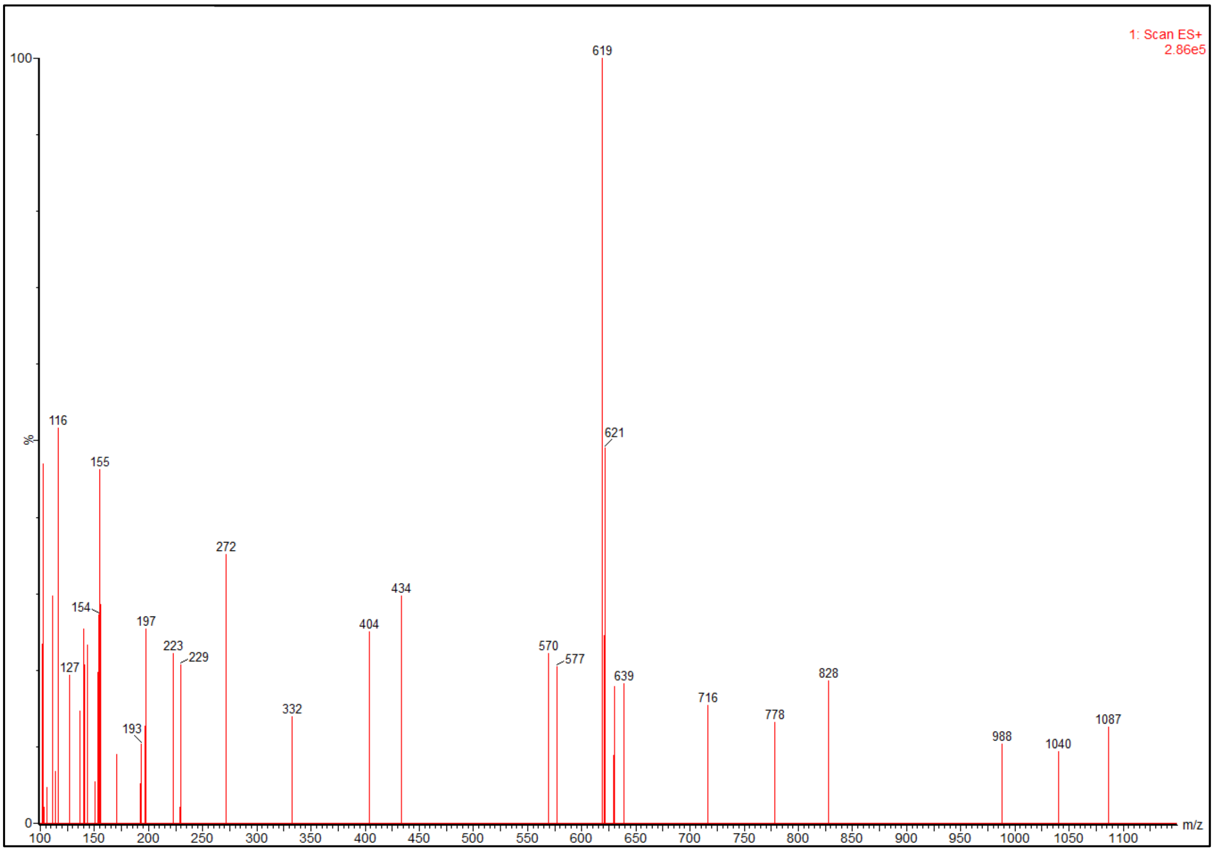

HPLC chromatogram of crude compound **3h** and corresponding mass spectrum

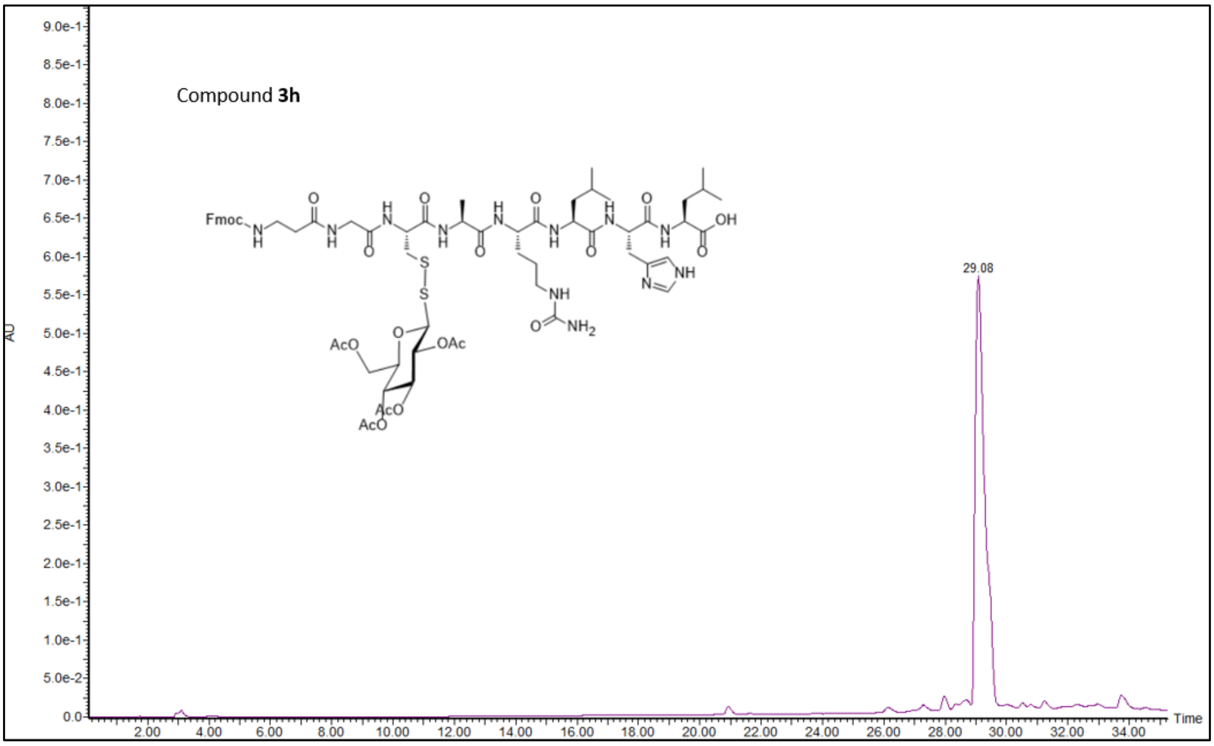

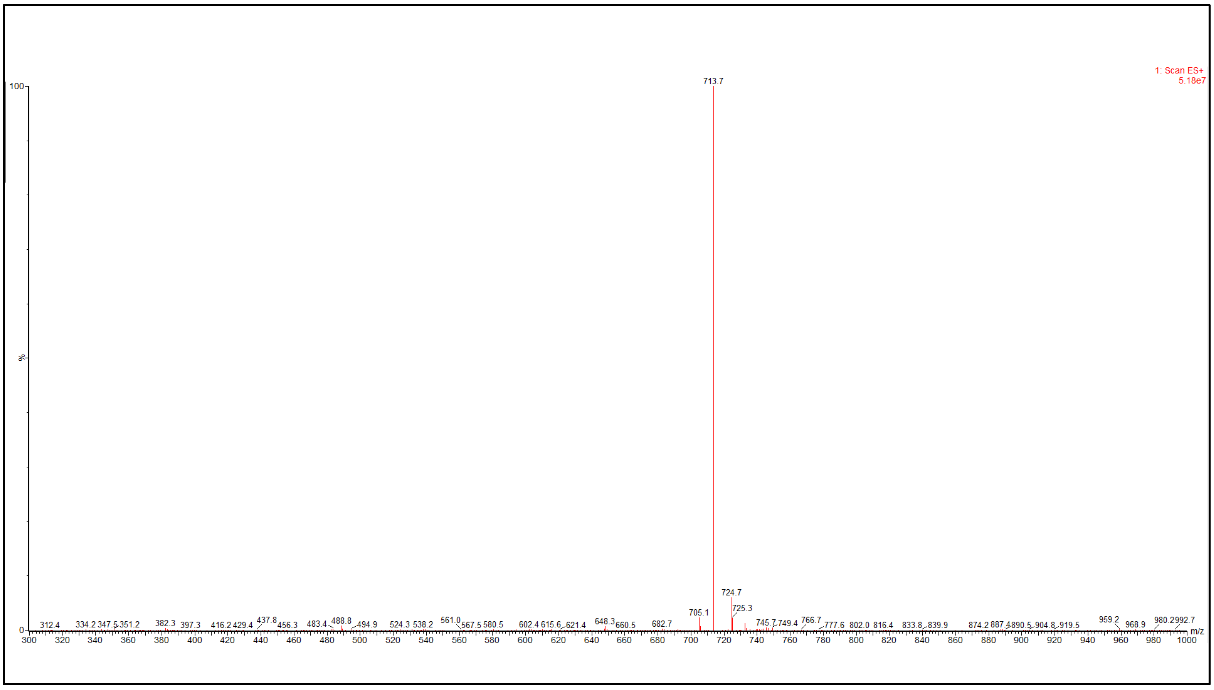

HPLC chromatogram of crude compound **3i** and corresponding mass spectrum

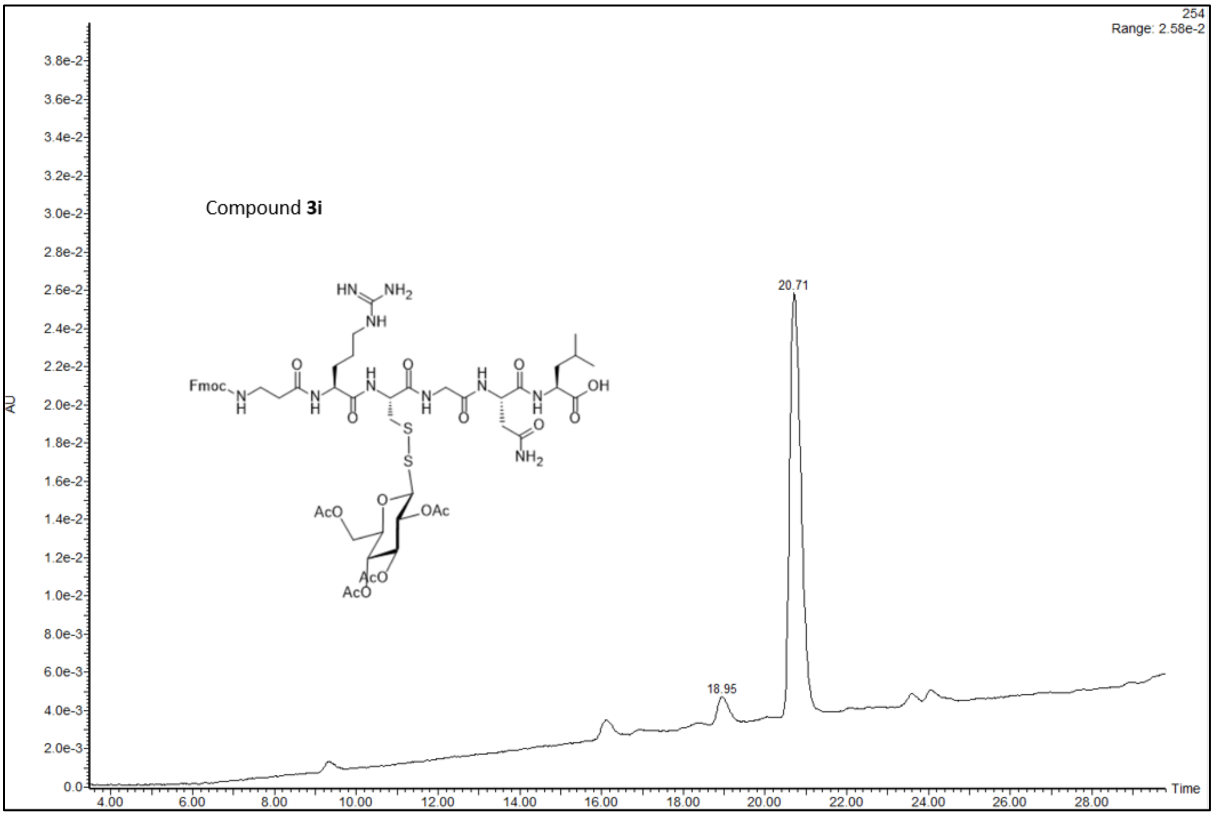

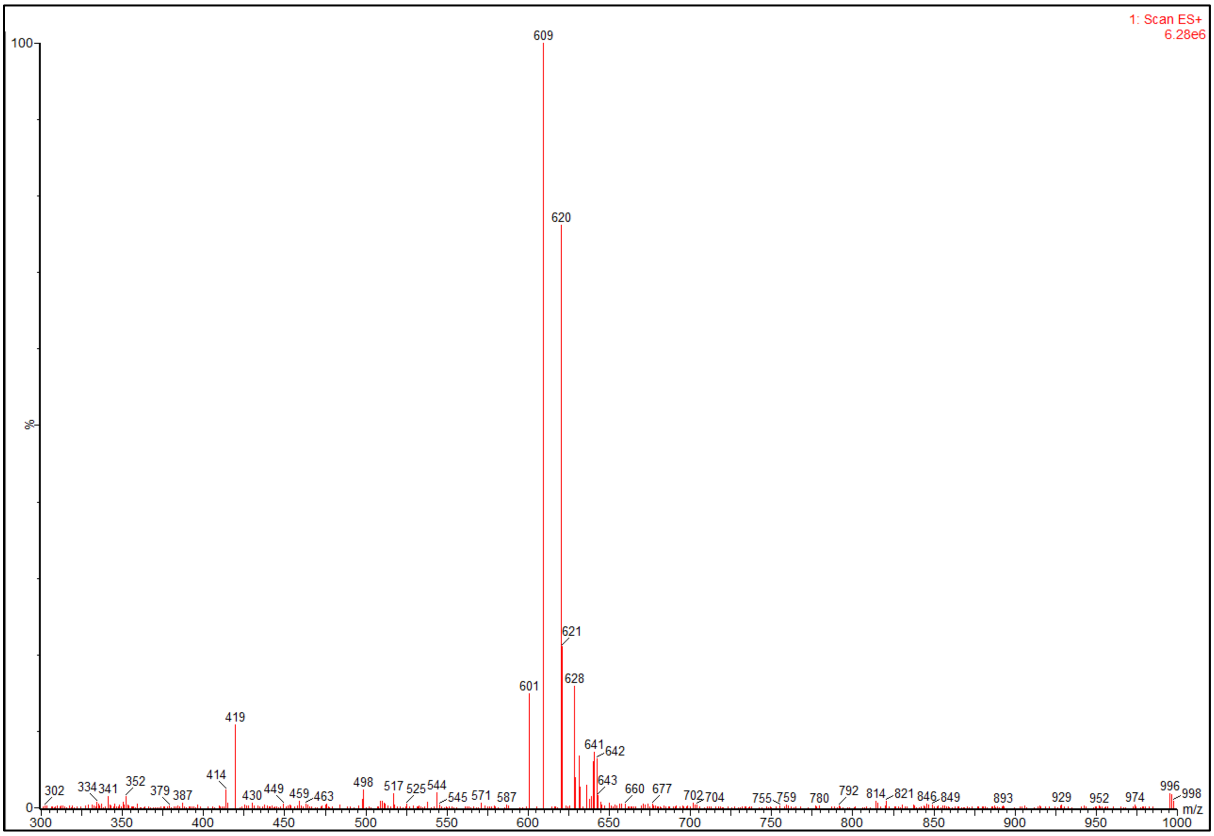

HPLC chromatogram of crude compound **3l** and corresponding mass spectrum

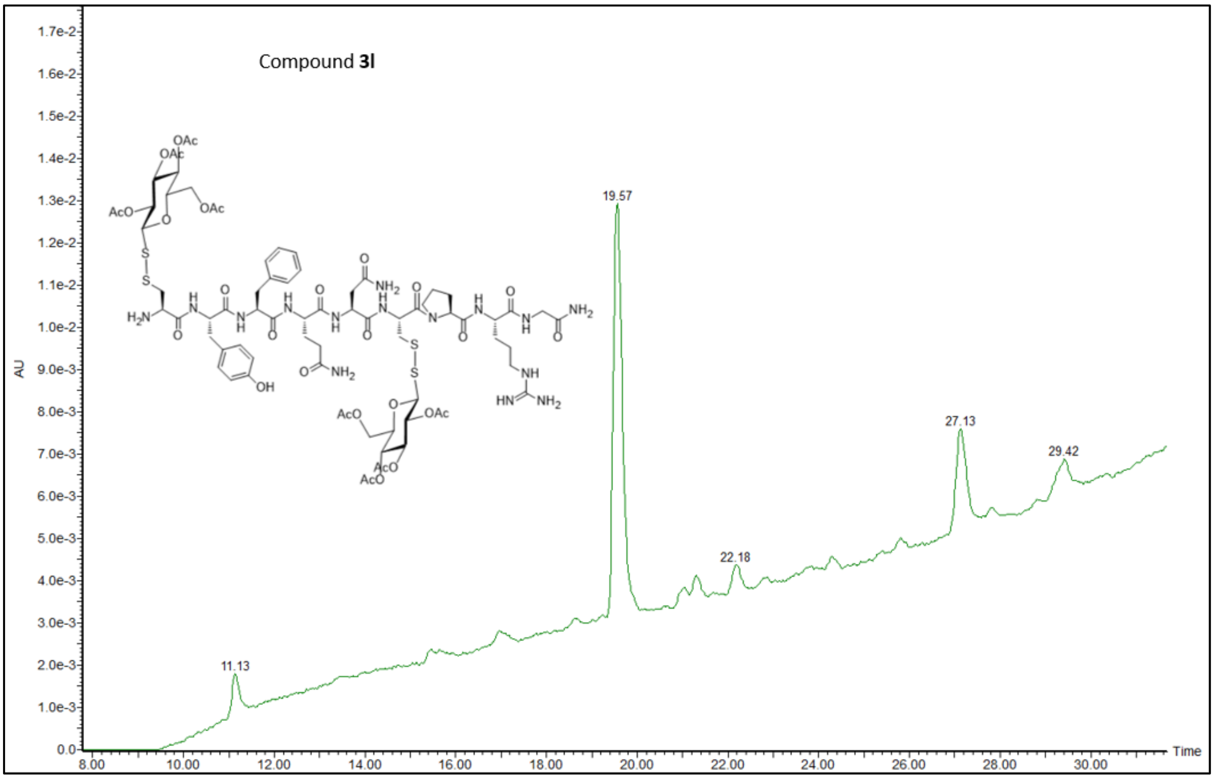

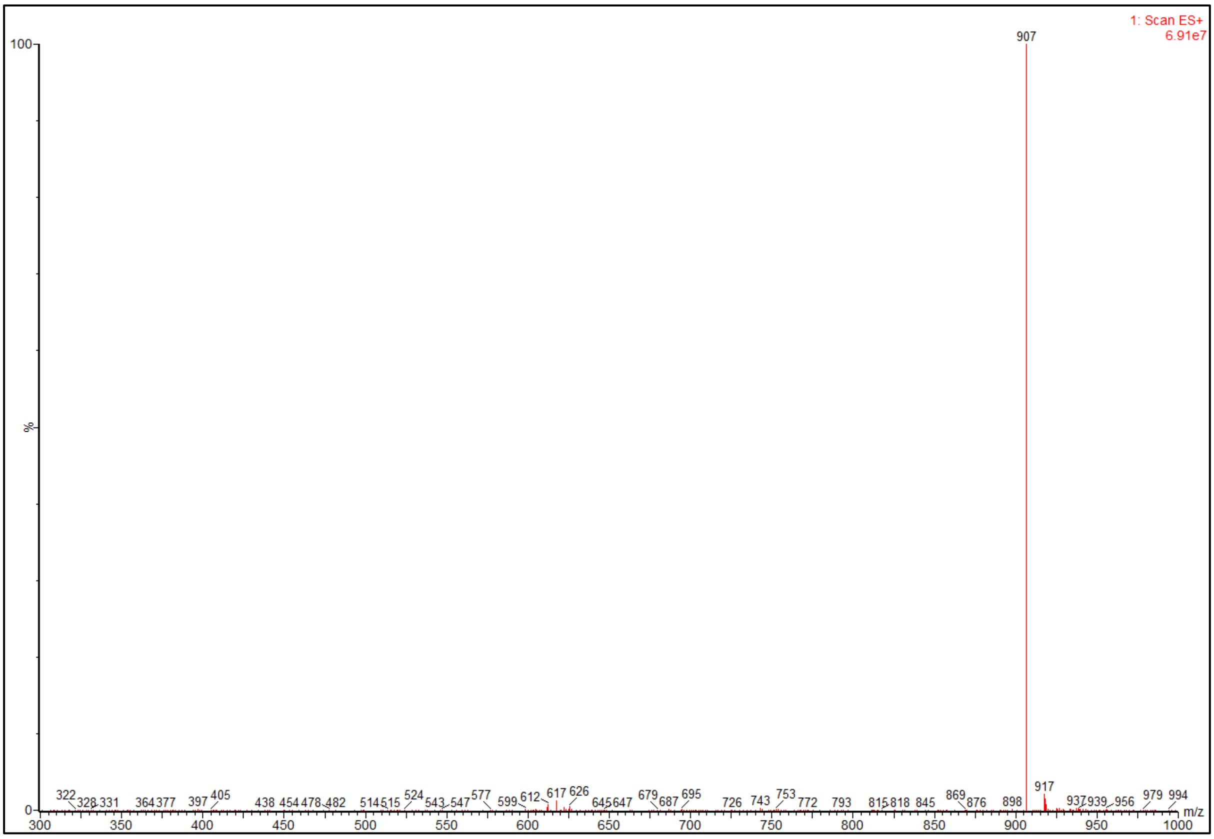

HPLC chromatogram of crude compound **3m** and corresponding mass spectrum

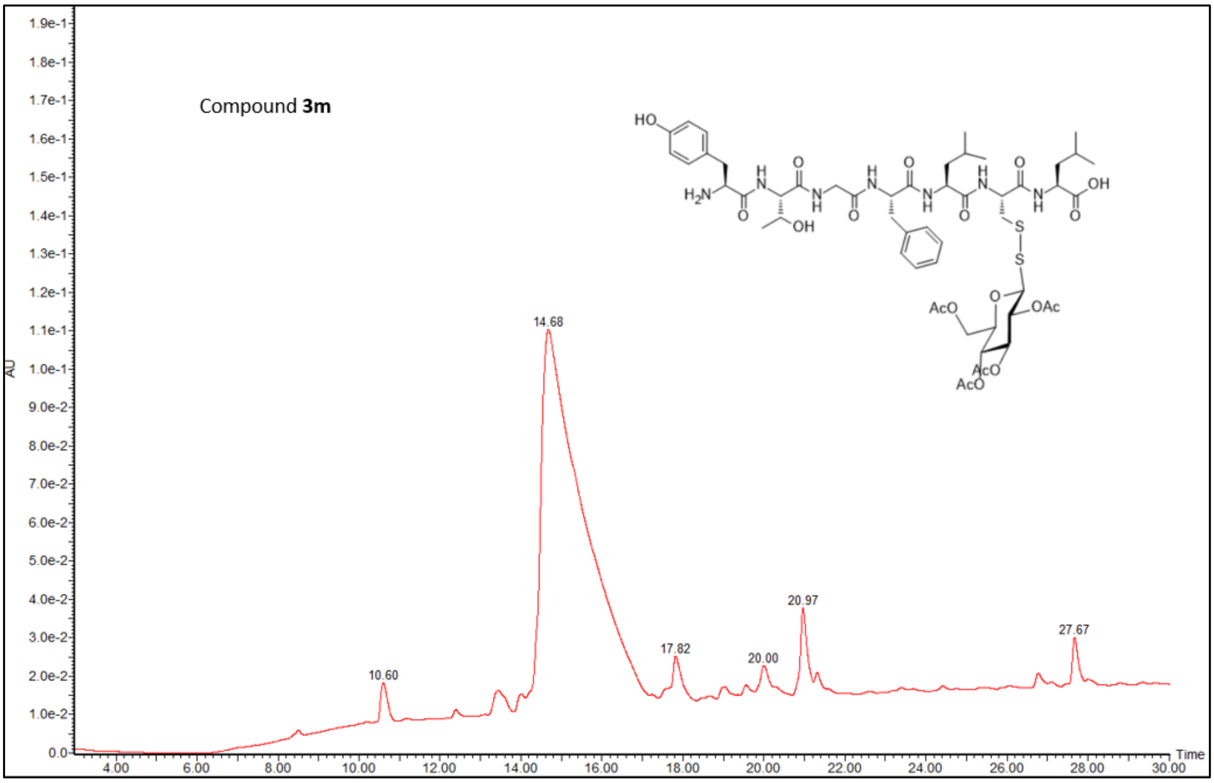

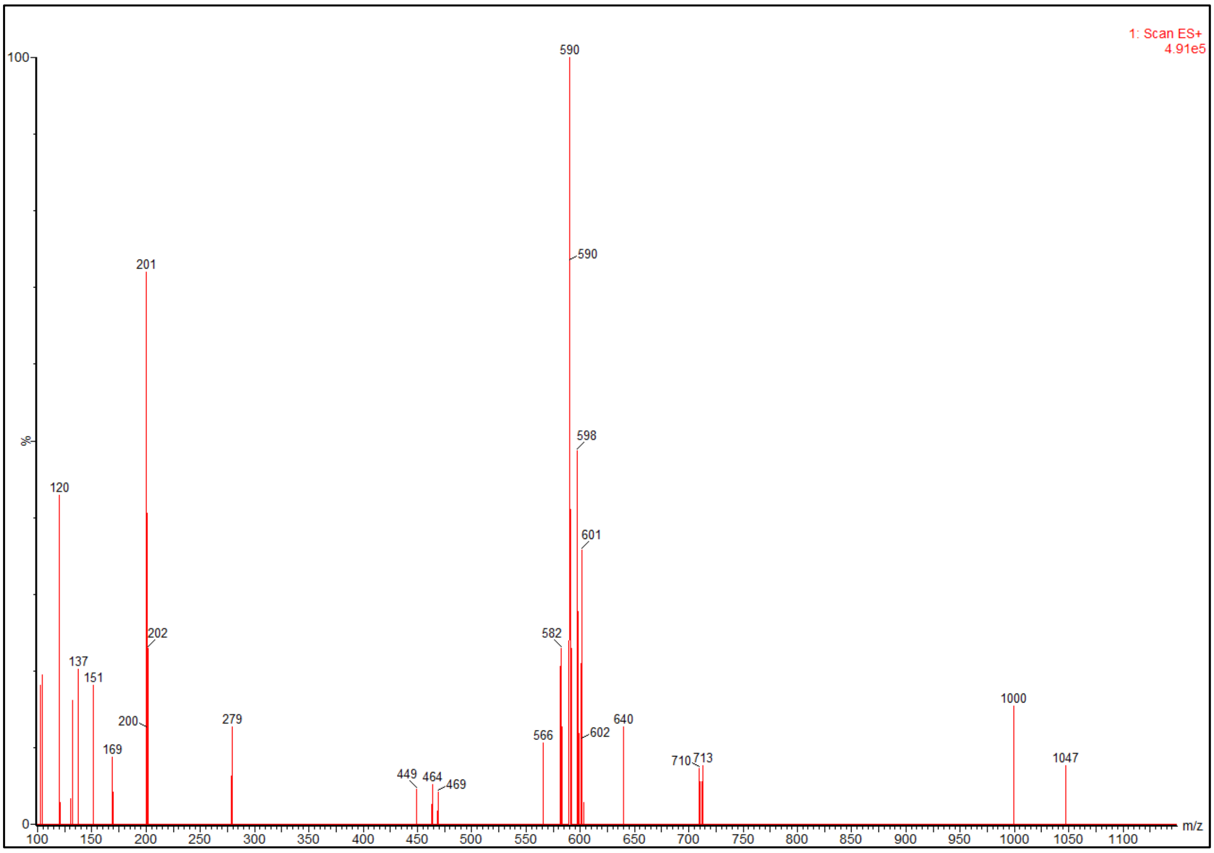

HPLC chromatogram of crude compound **8e** and corresponding mass spectrum

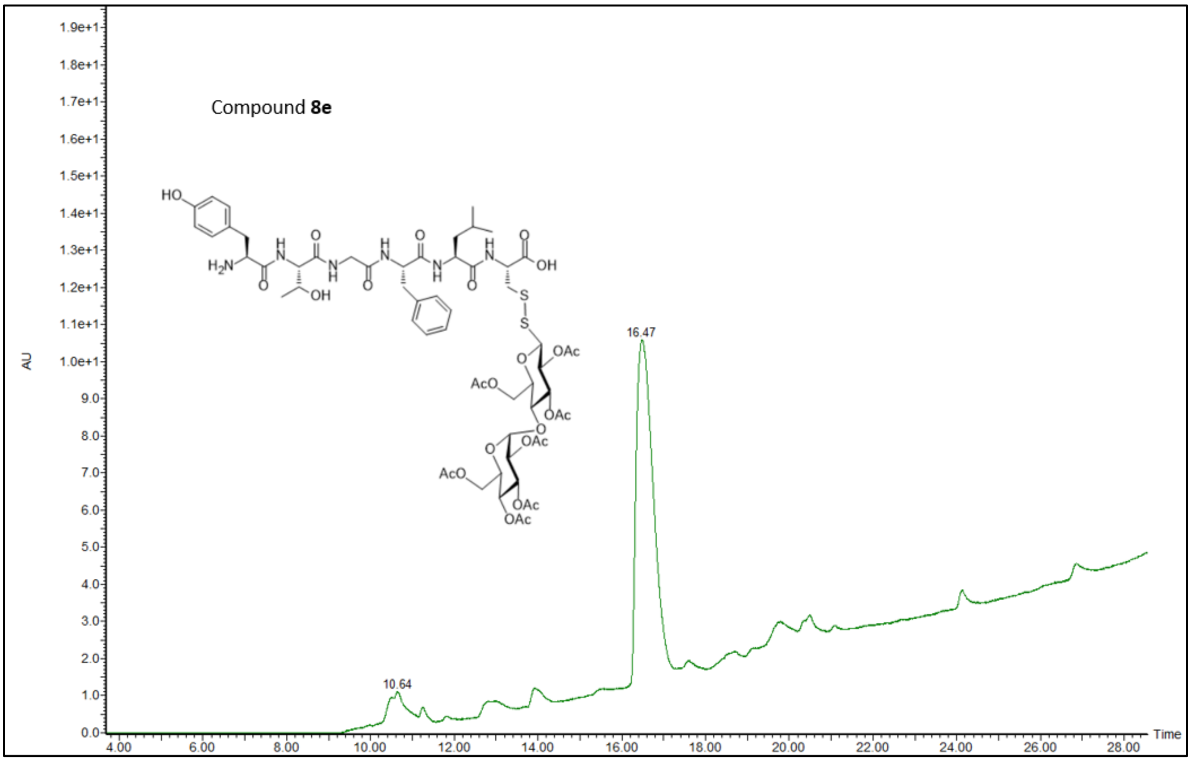

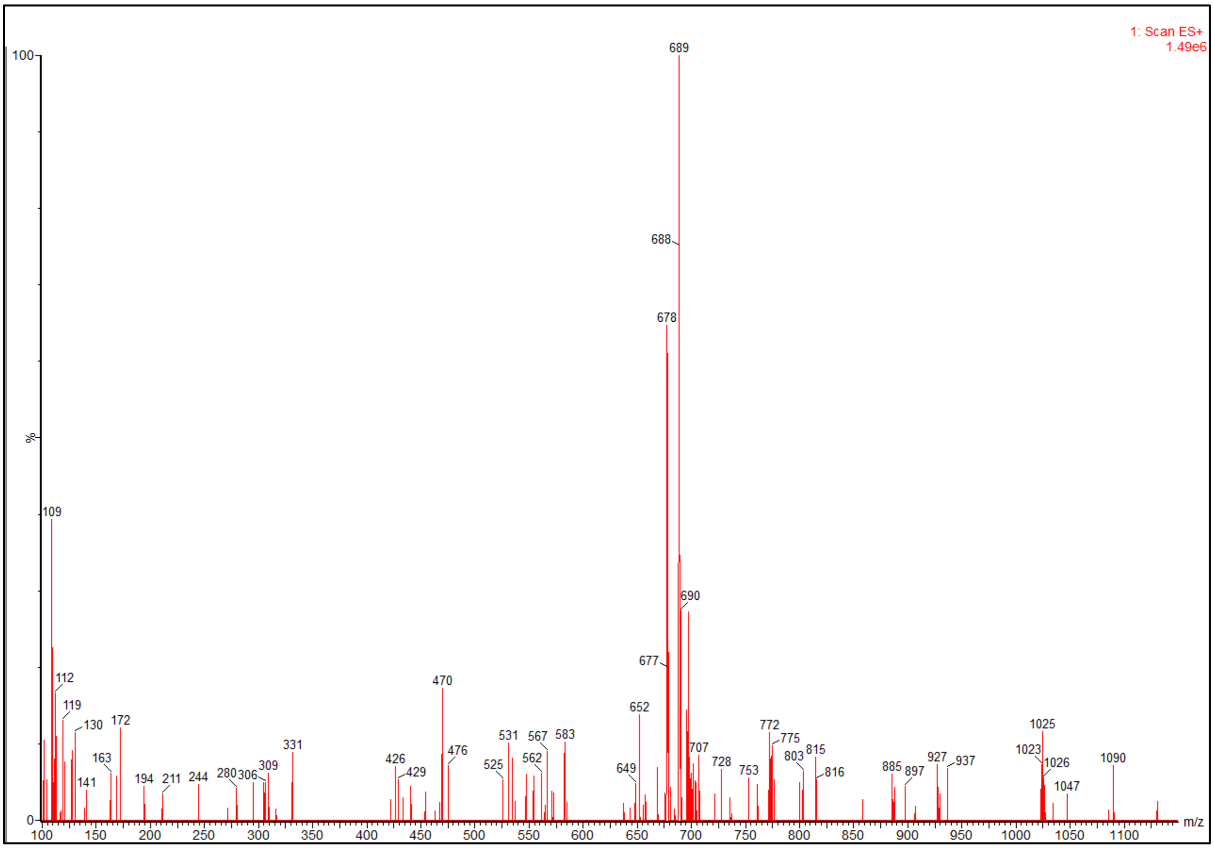

HPLC chromatogram of crude compound **8g** and corresponding mass spectrum

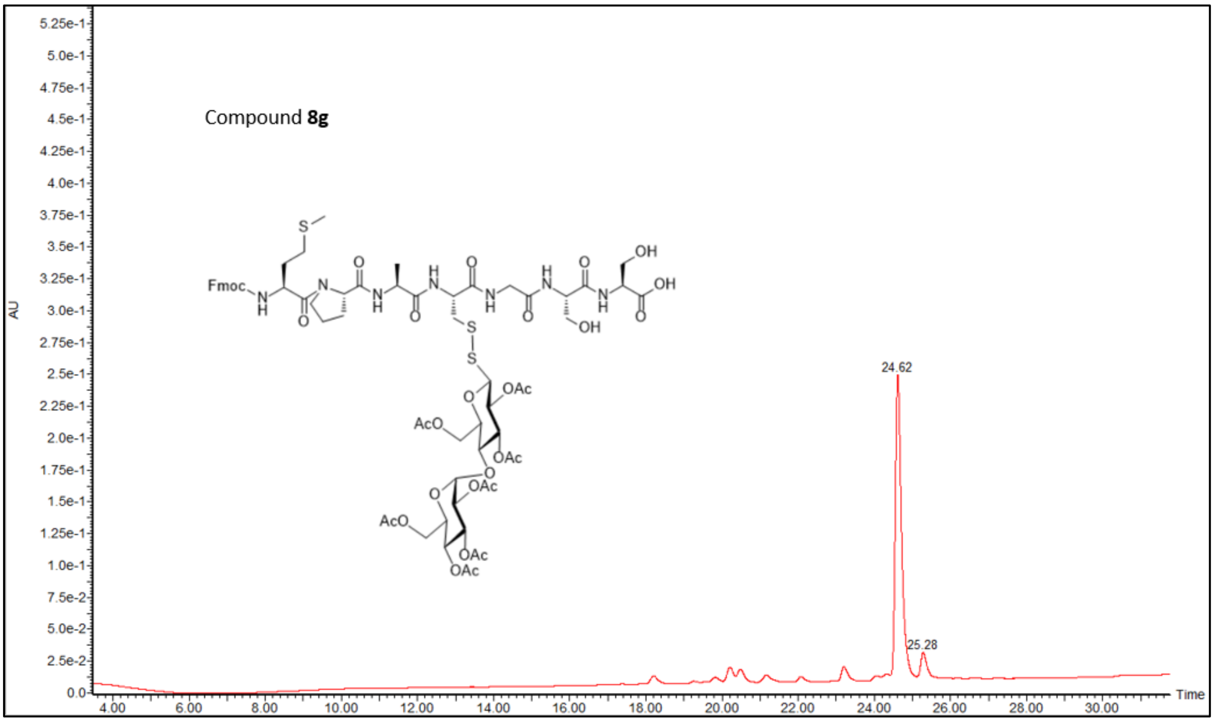

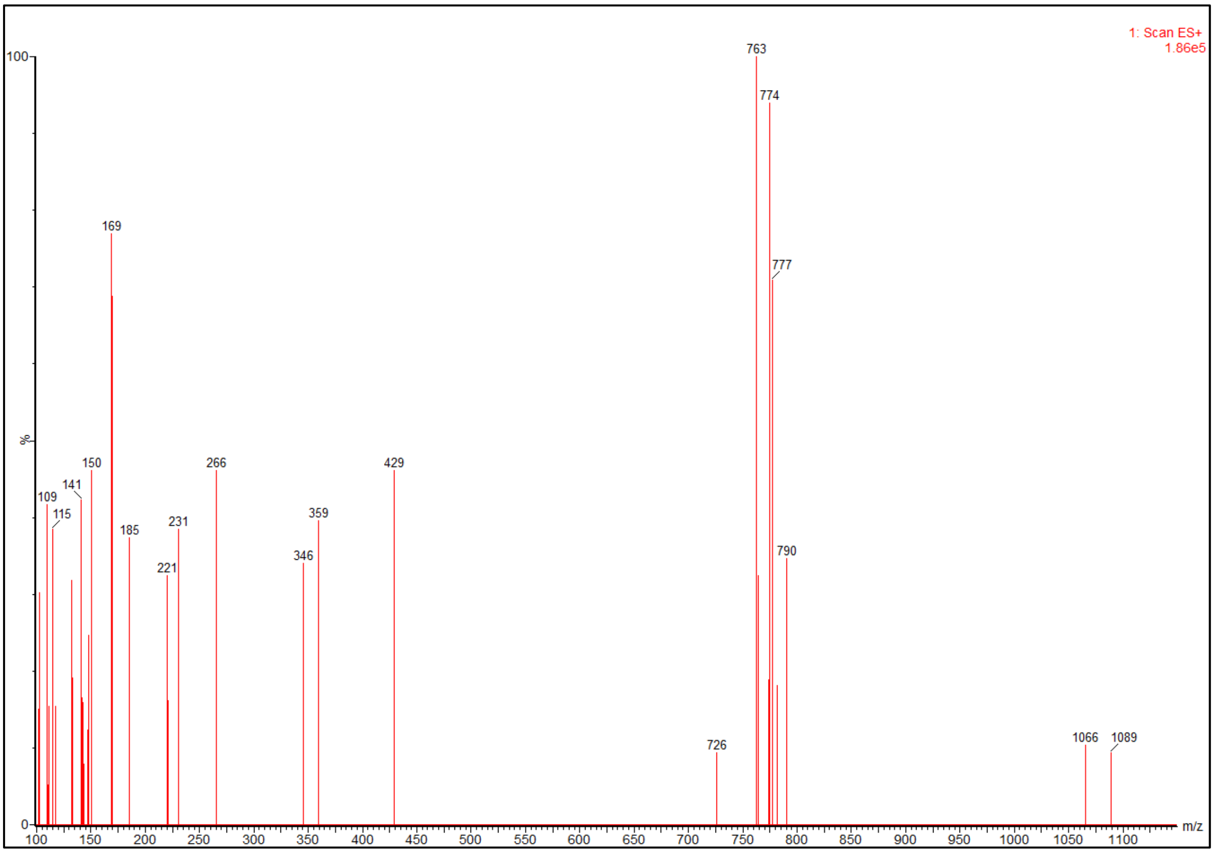

HPLC chromatogram of crude compound **8h** and corresponding mass spectrum

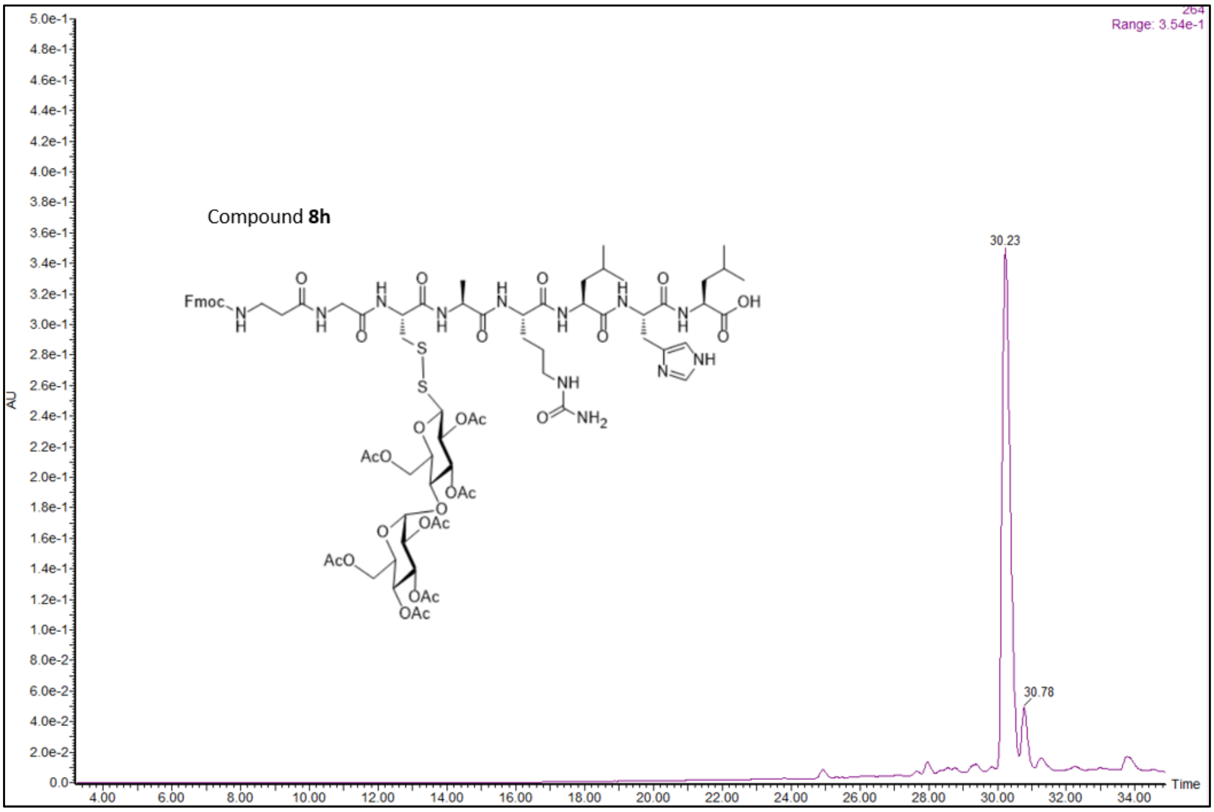

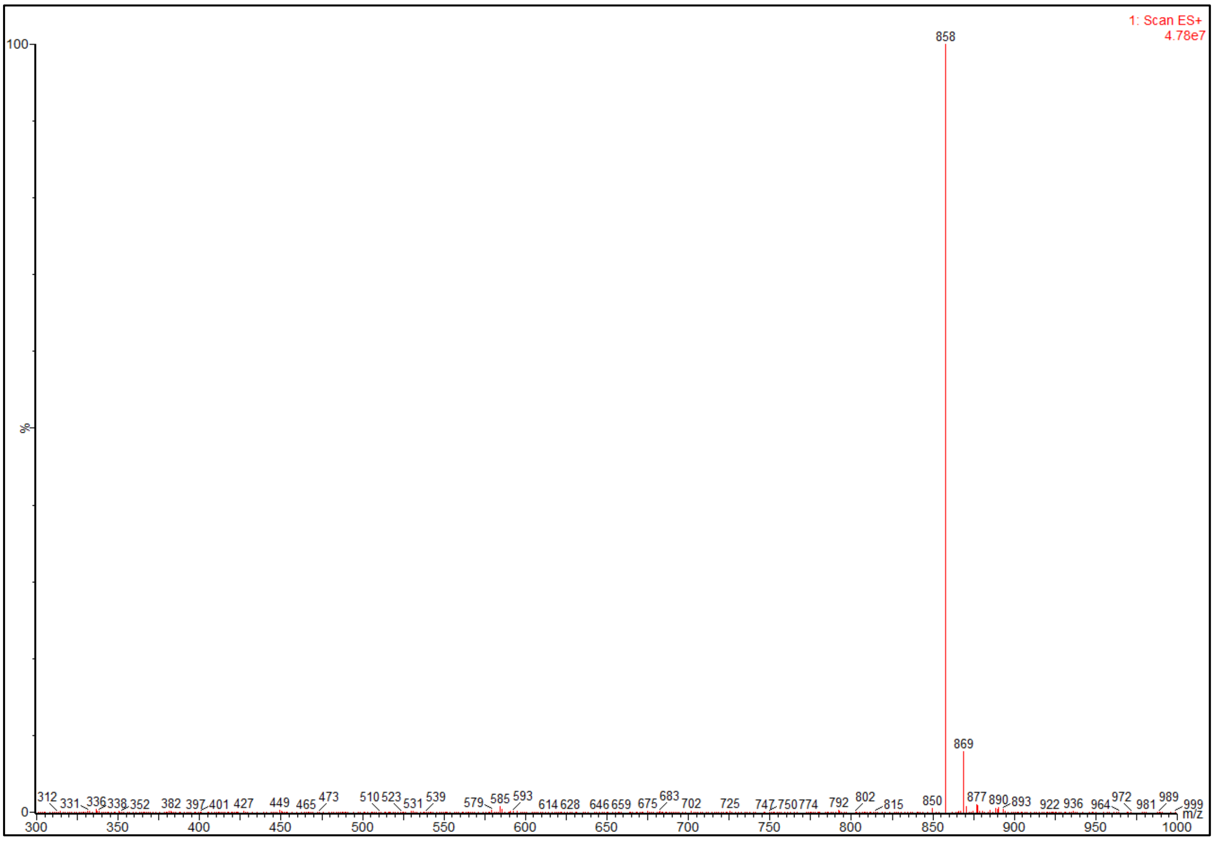

HPLC chromatogram of crude compound **8i** and corresponding mass spectrum

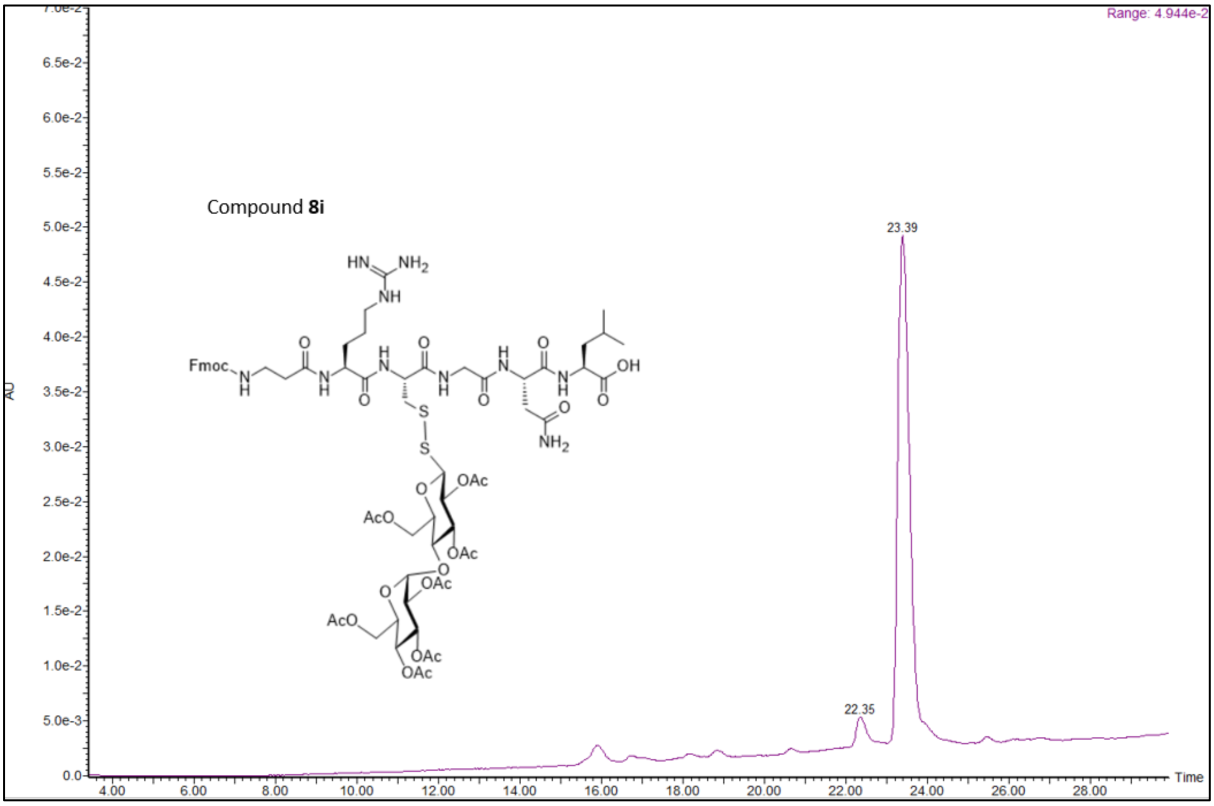

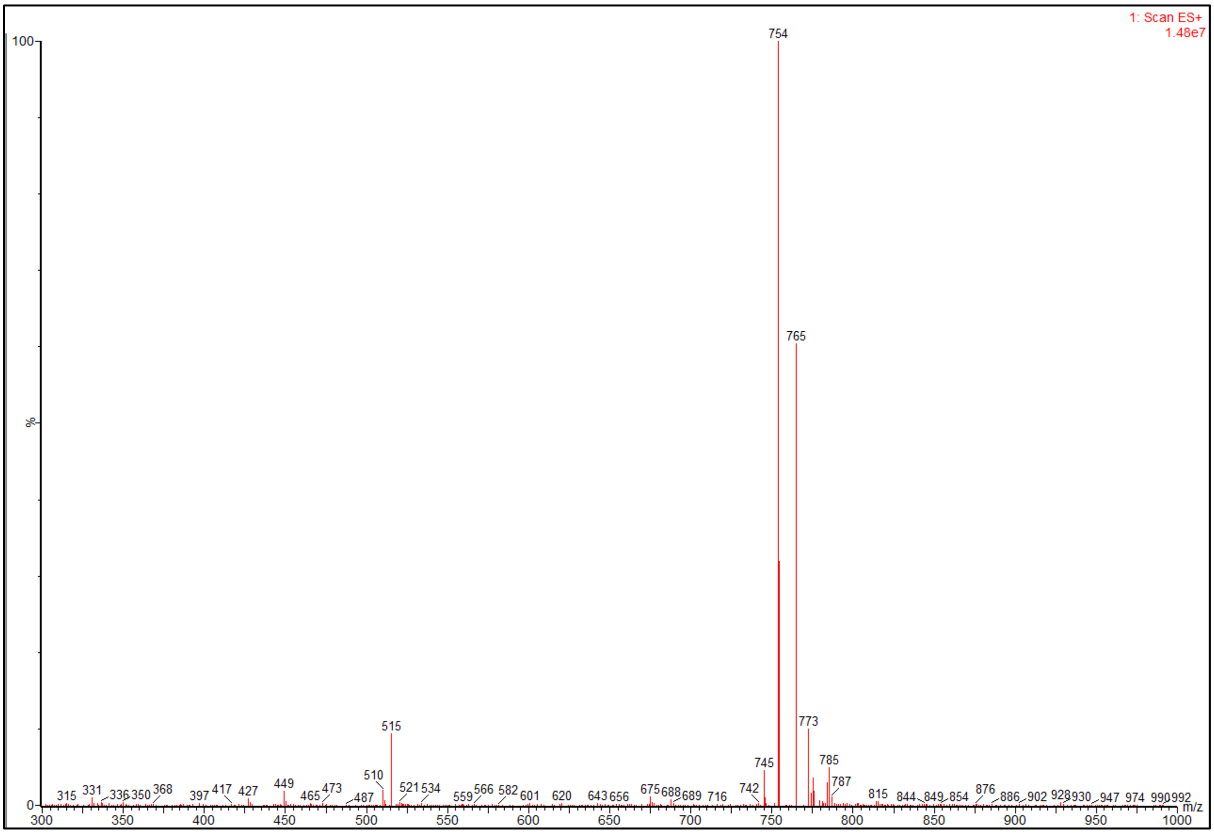

HPLC chromatogram of crude compound **11c** and corresponding mass spectrum

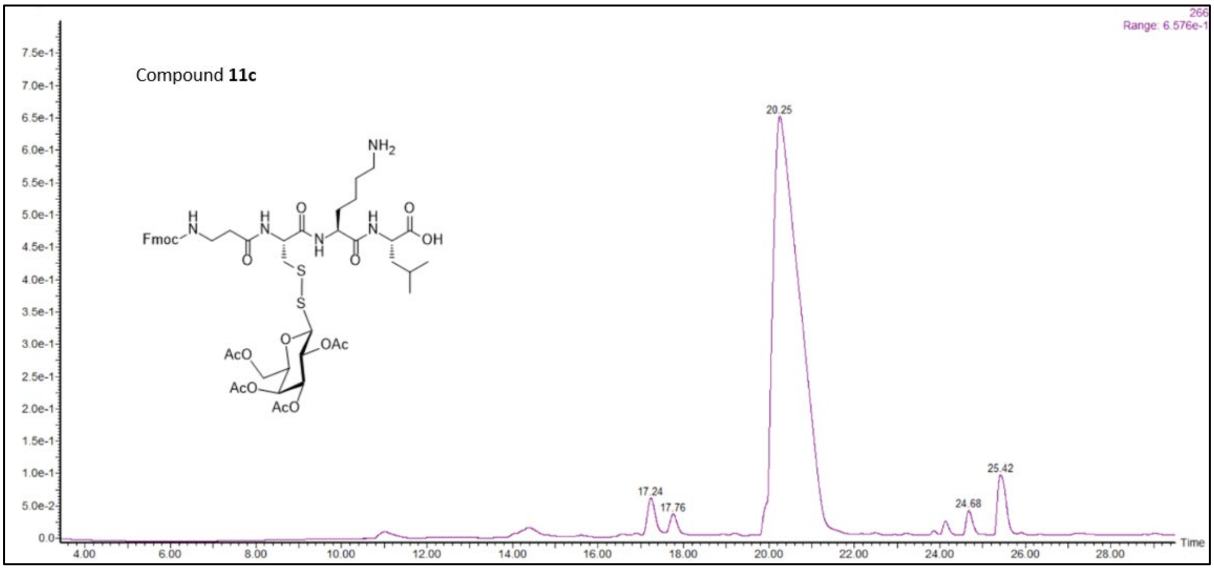

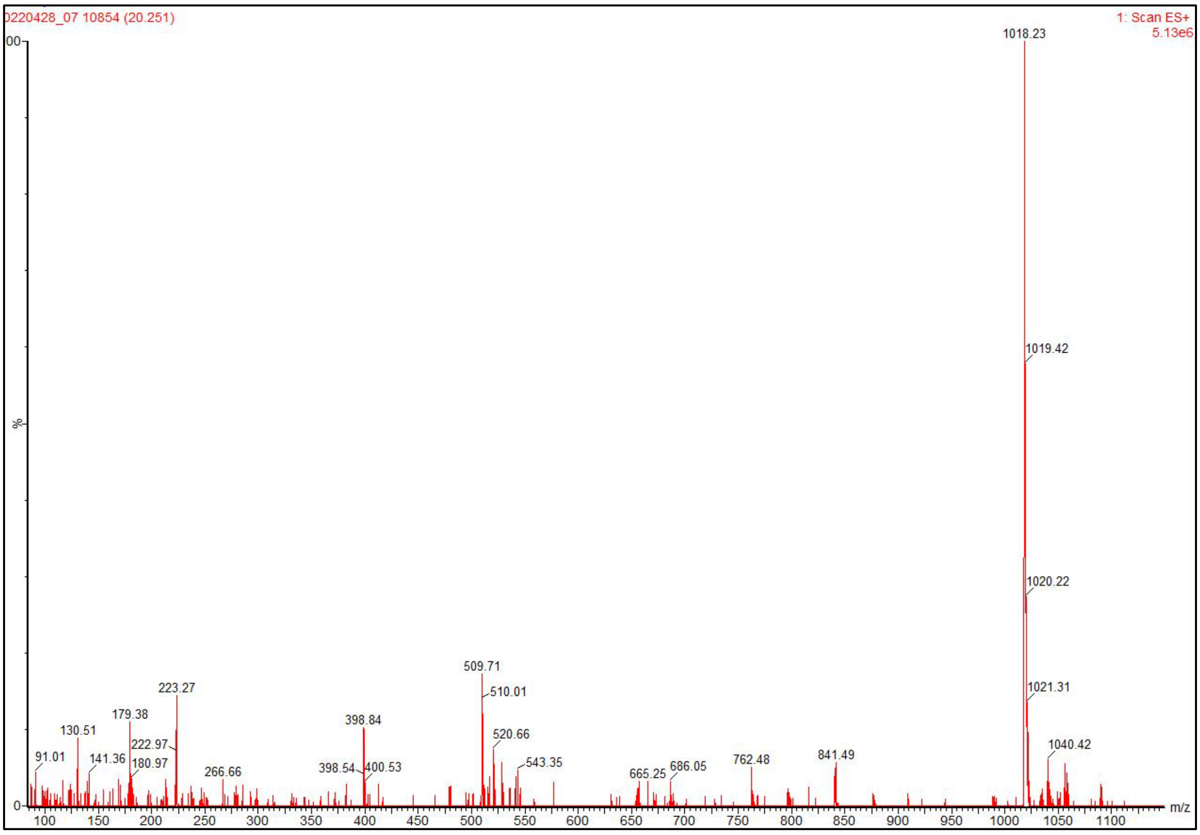

HPLC chromatogram of crude compound **11g** and corresponding mass spectrum

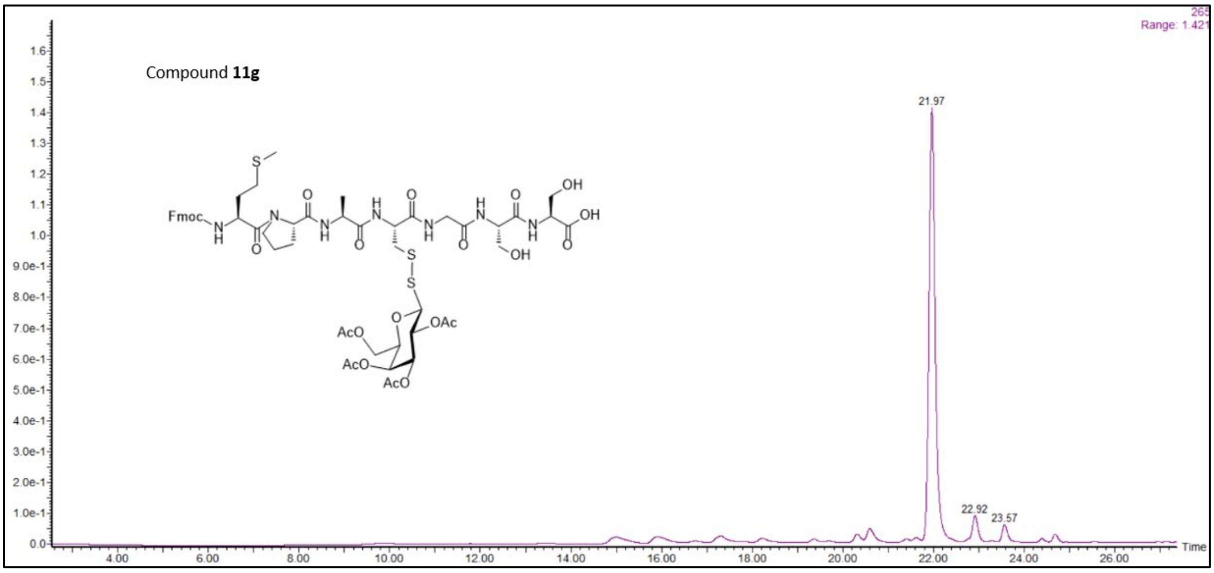

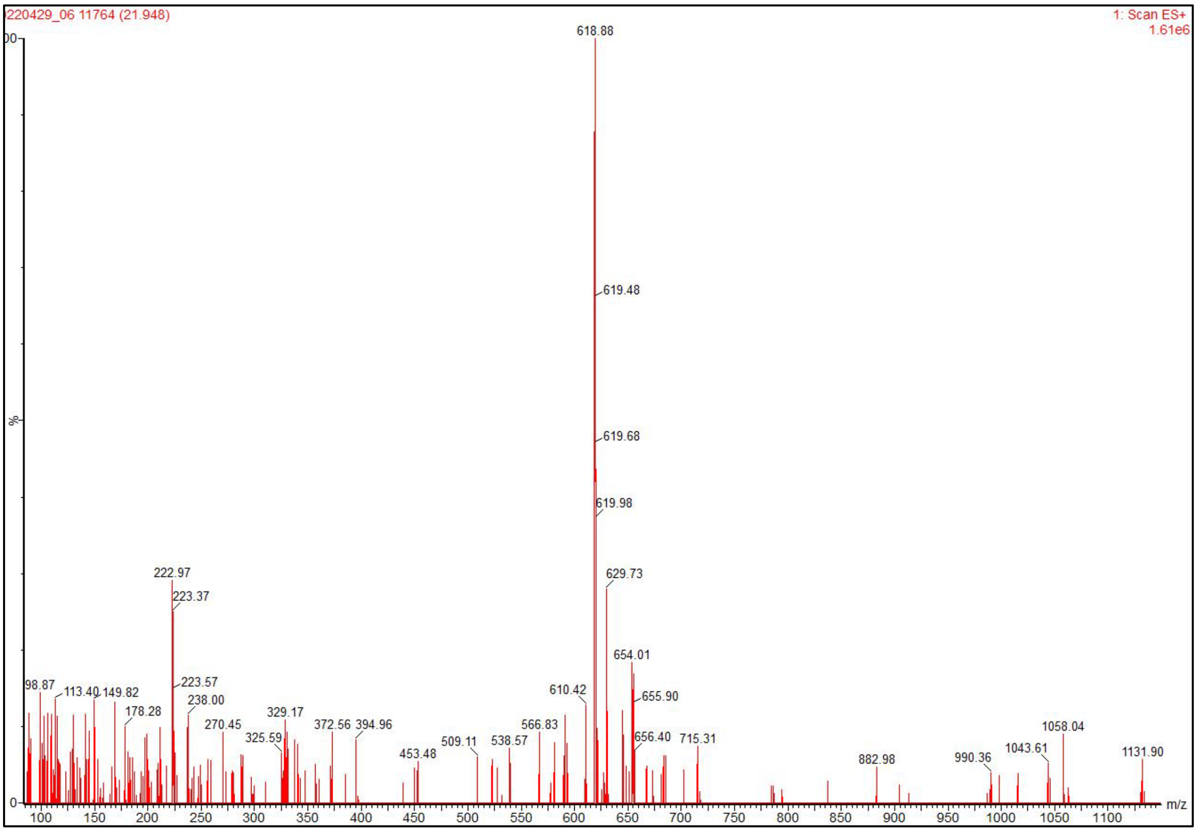

HPLC chromatogram of crude compound **11h** and corresponding mass spectrum

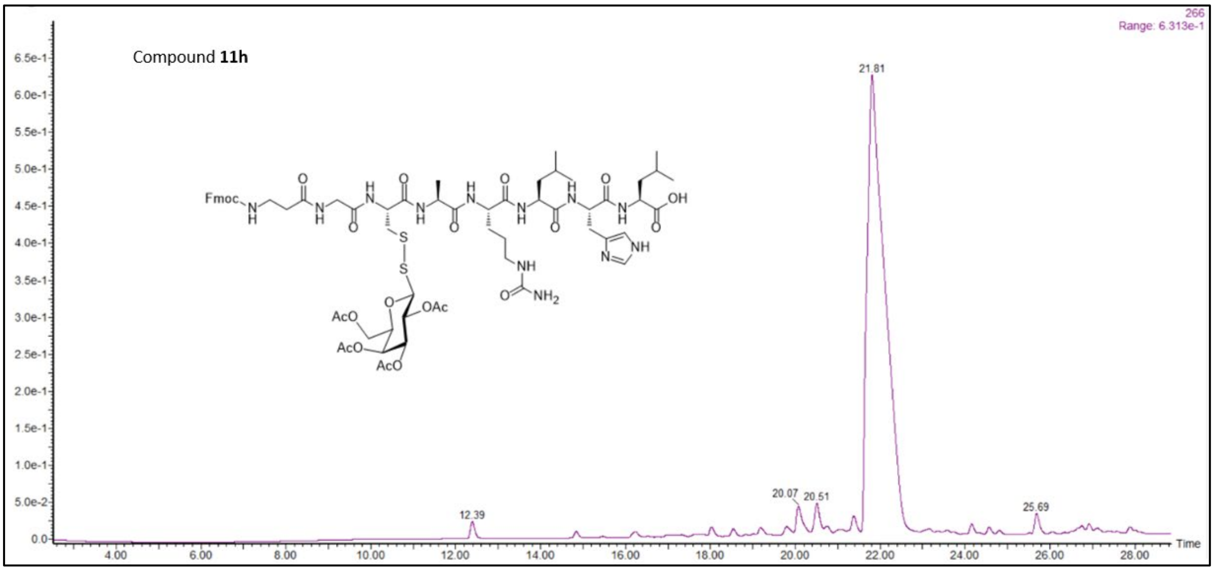

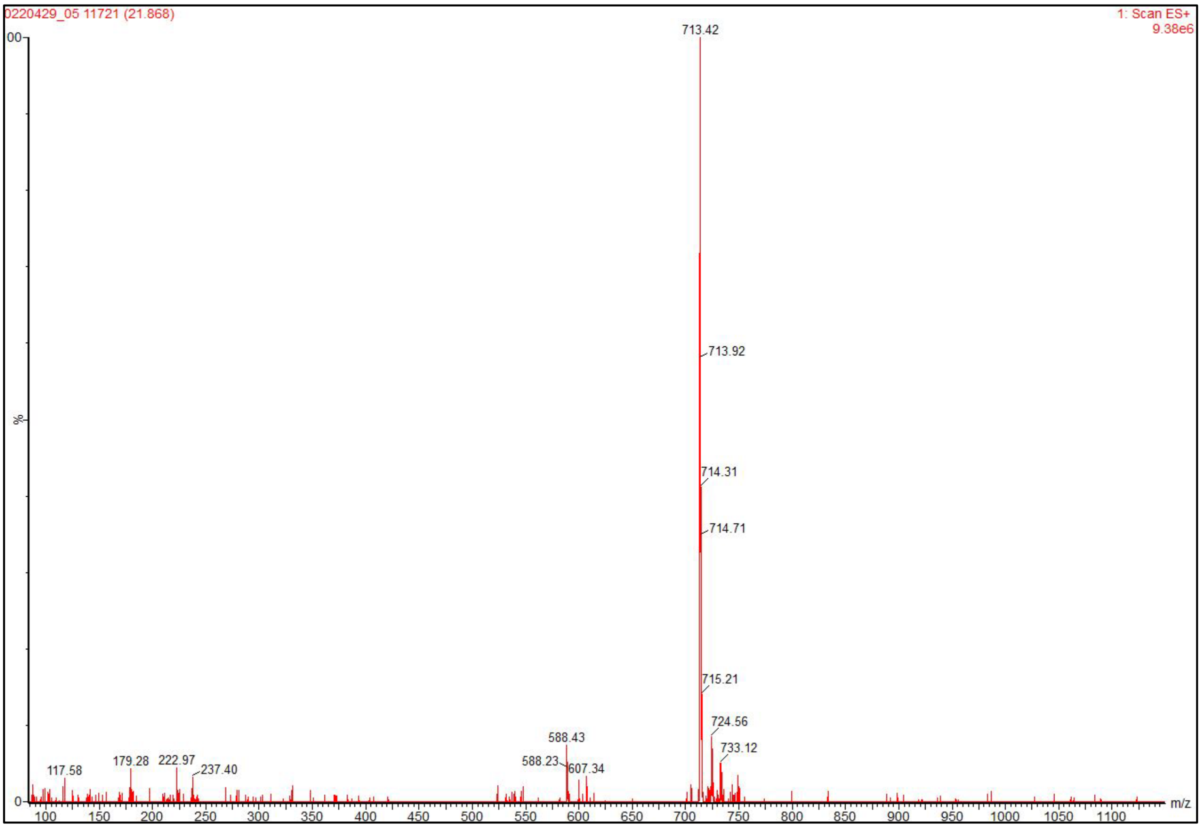

HPLC chromatogram of crude compound **11i** and corresponding mass spectrum

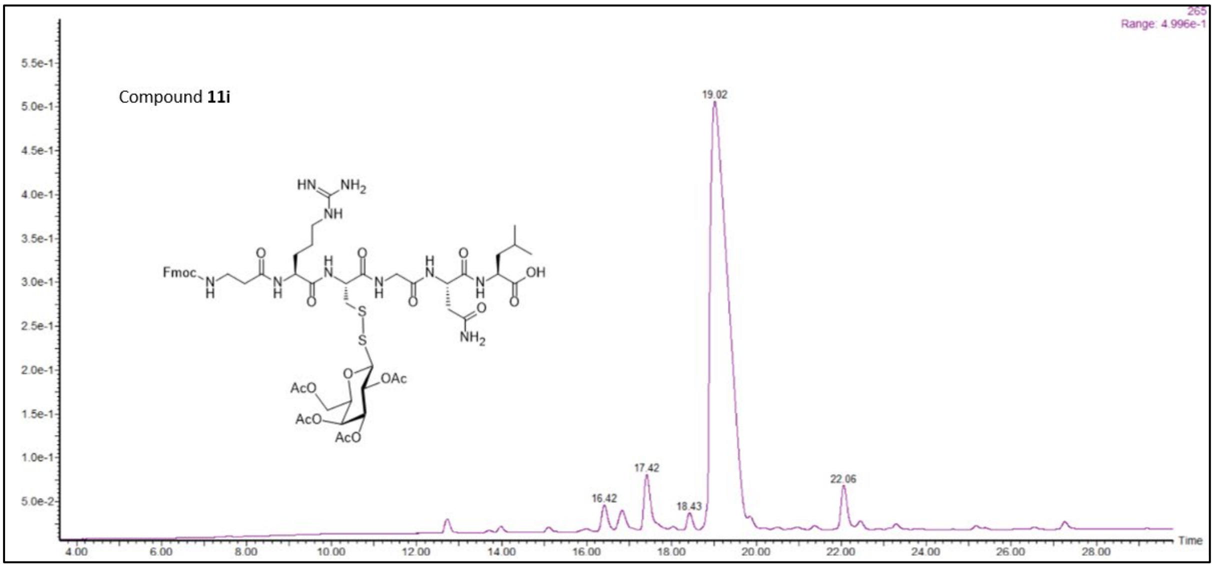

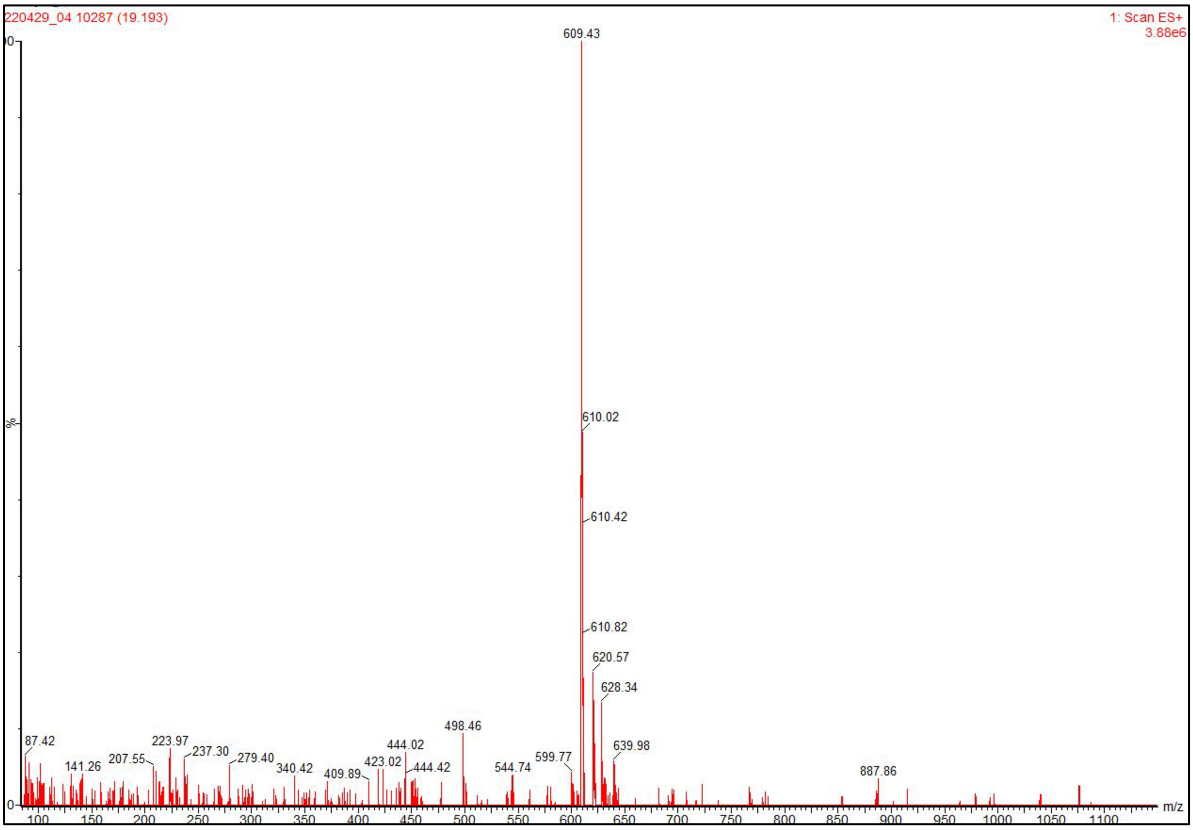

HPLC chromatogram of crude compound **14g** and corresponding mass spectrum

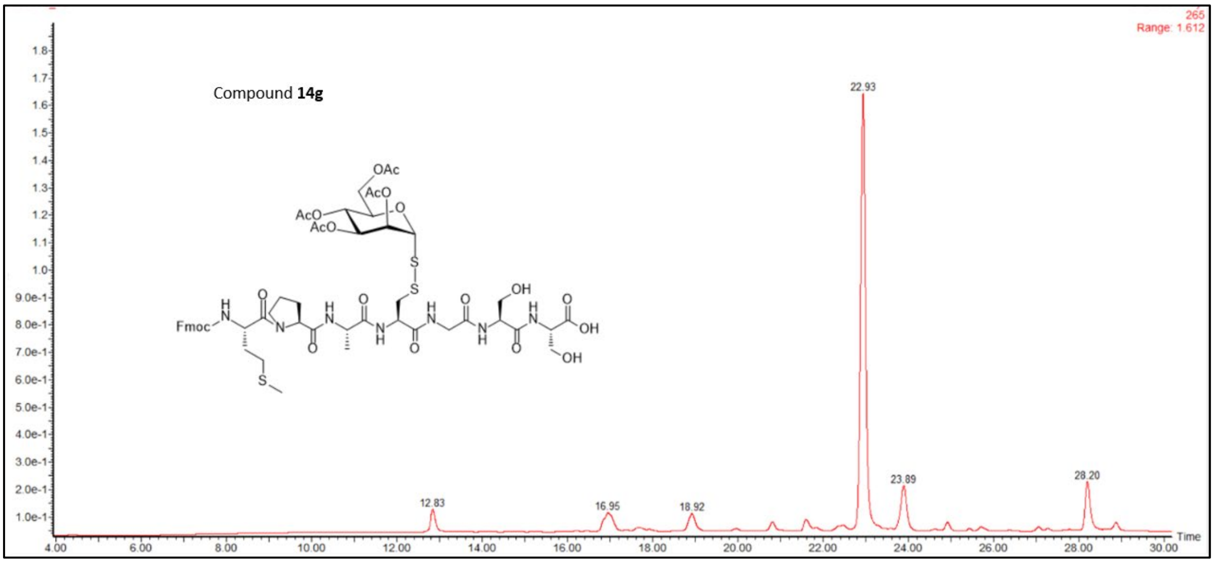

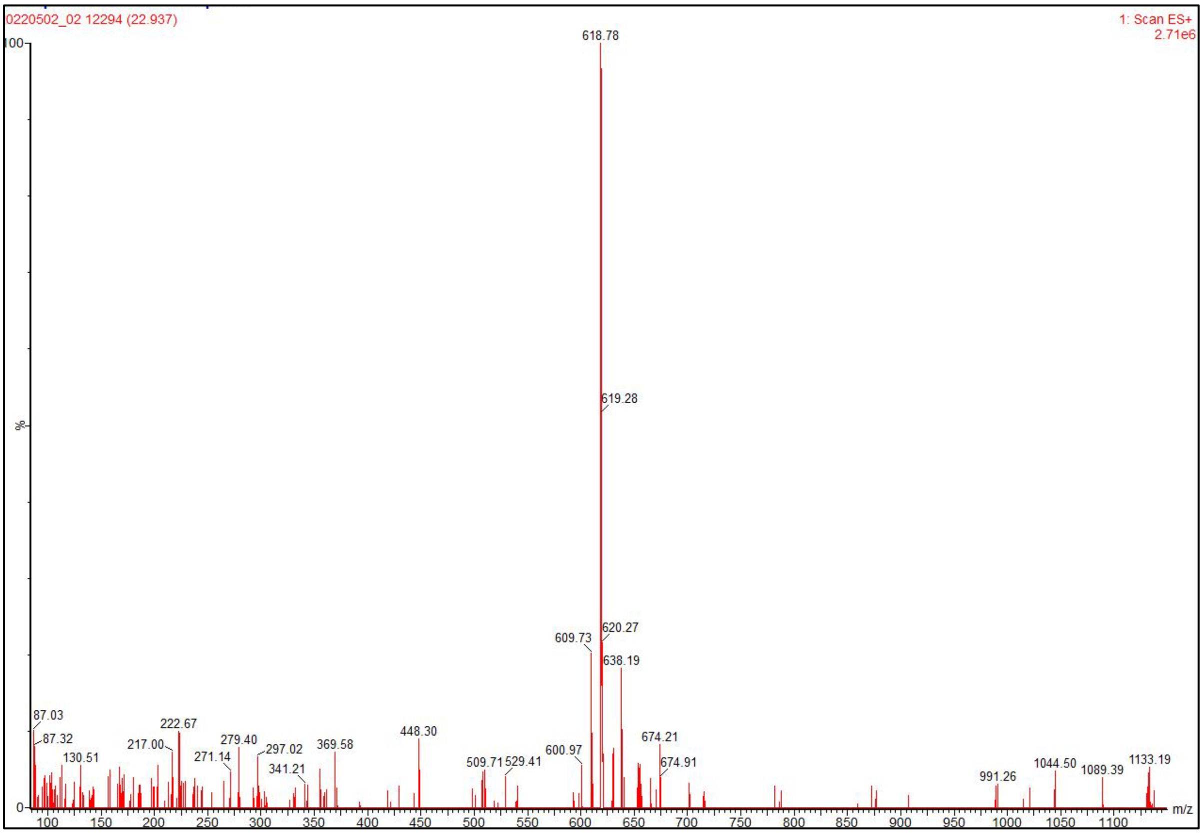

HPLC chromatogram of crude compound **23g** and corresponding mass spectrum

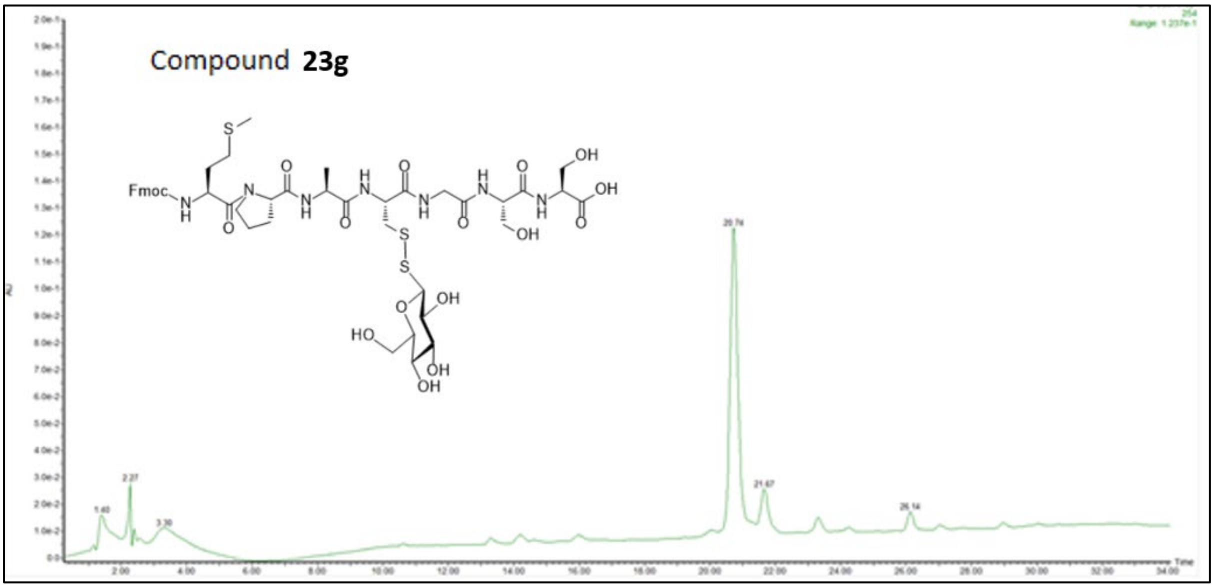

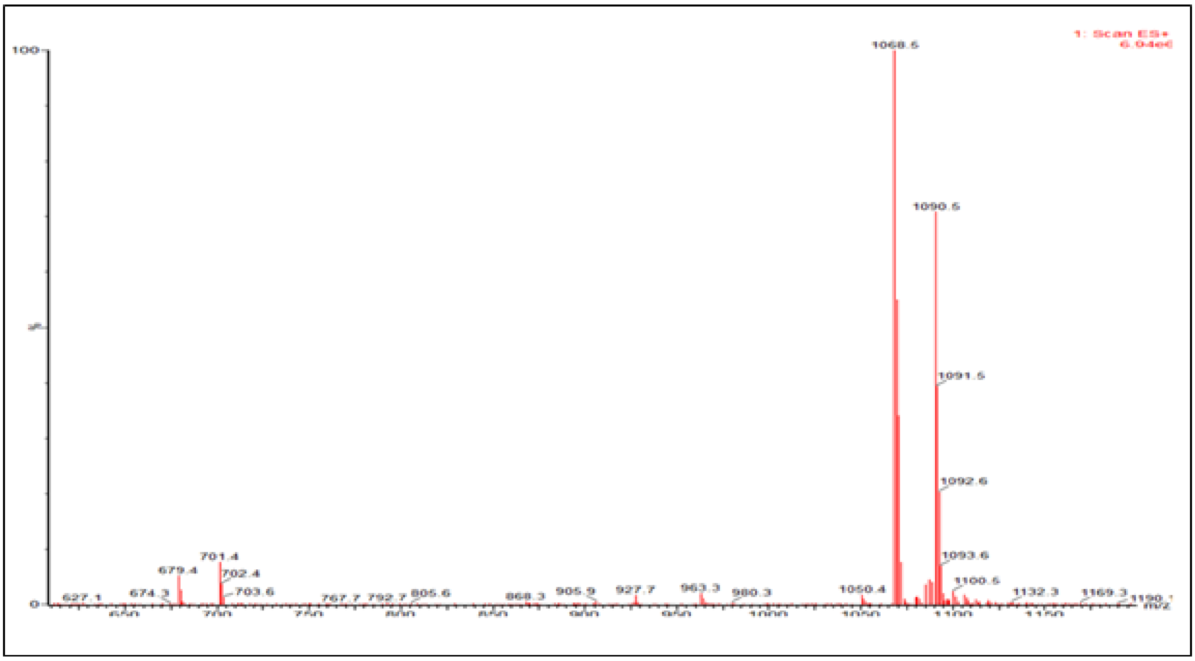

HPLC chromatogram of crude compound **23h** and corresponding mass spectrum

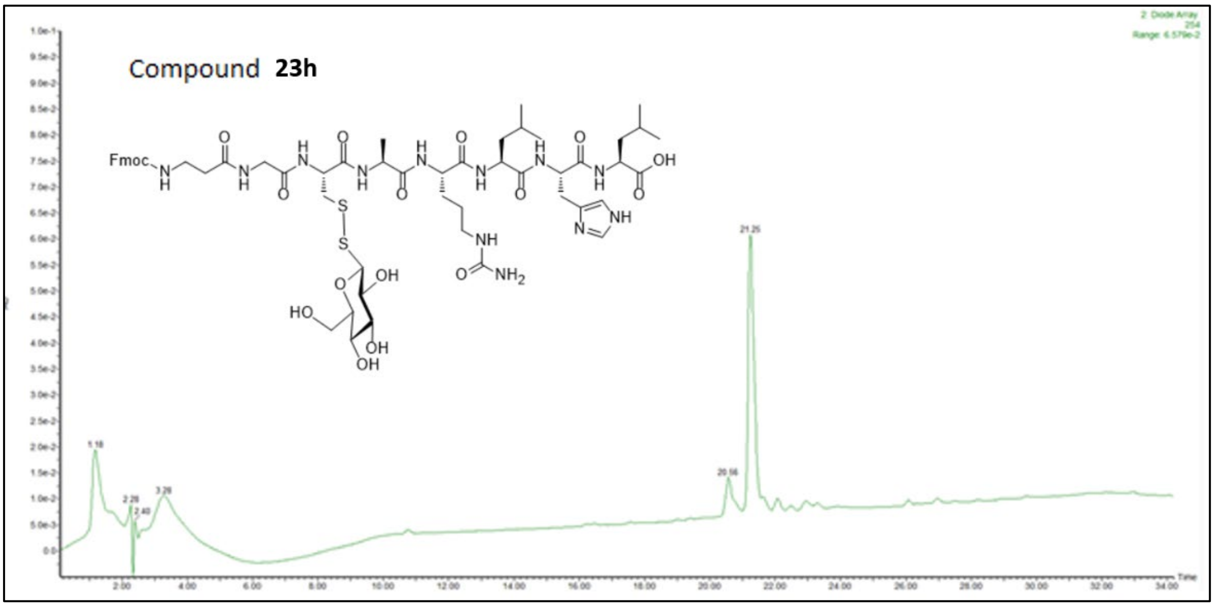

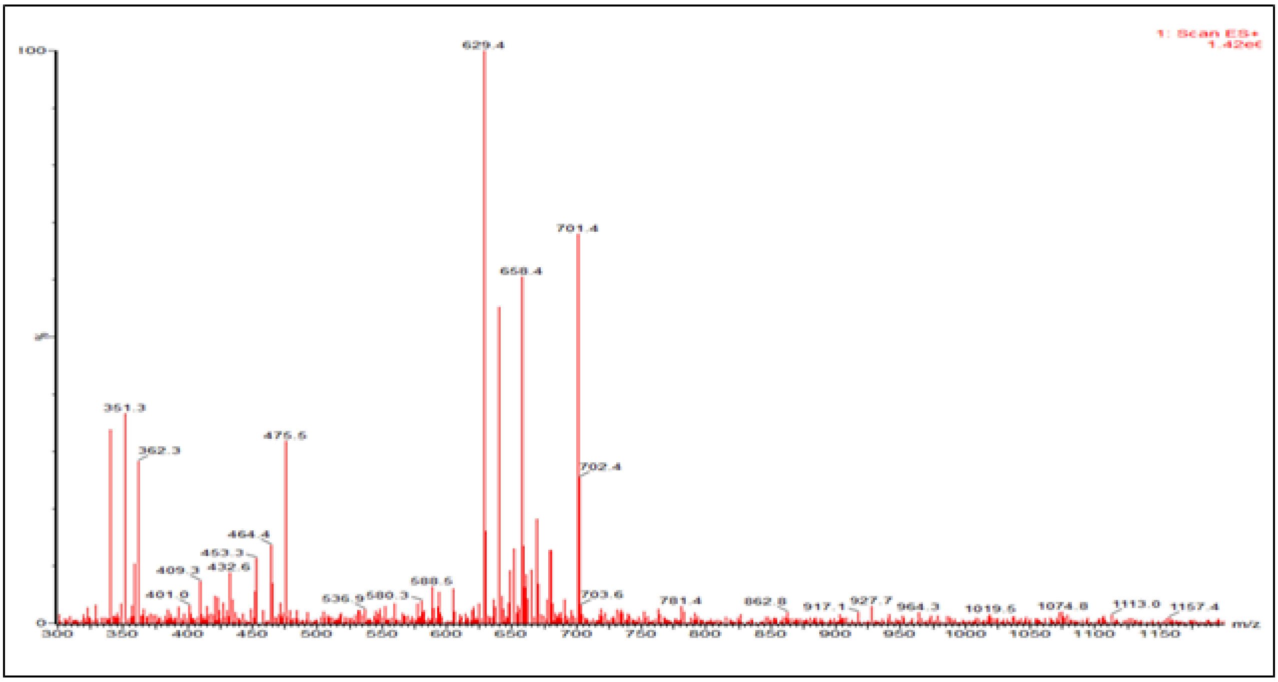

HPLC chromatogram of crude compound **23i** and corresponding mass spectrum

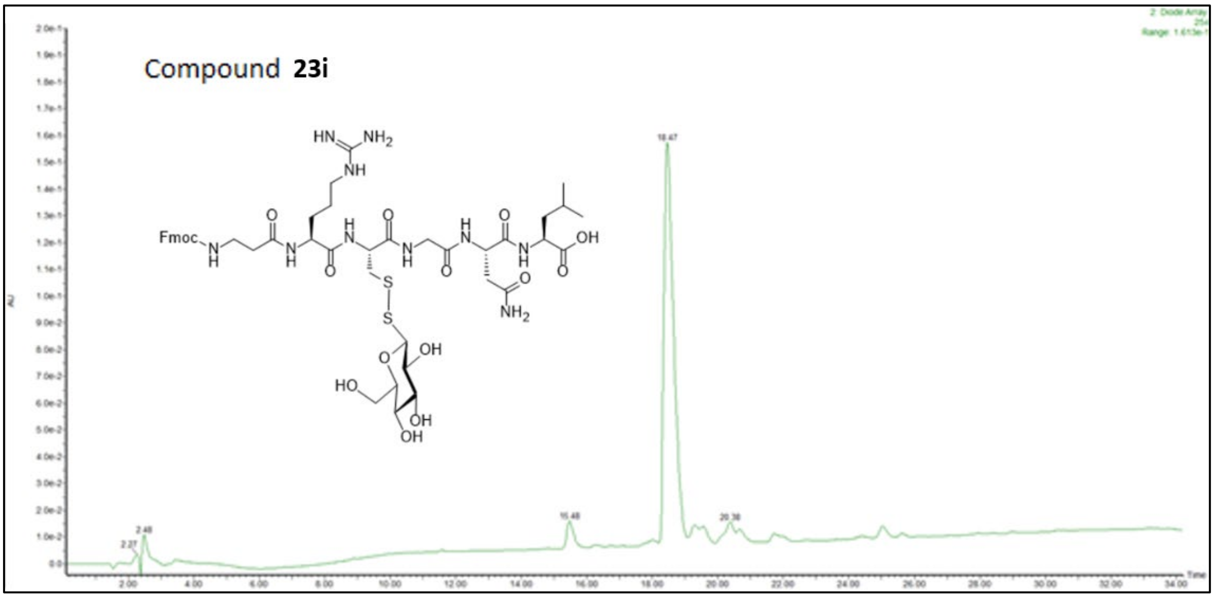

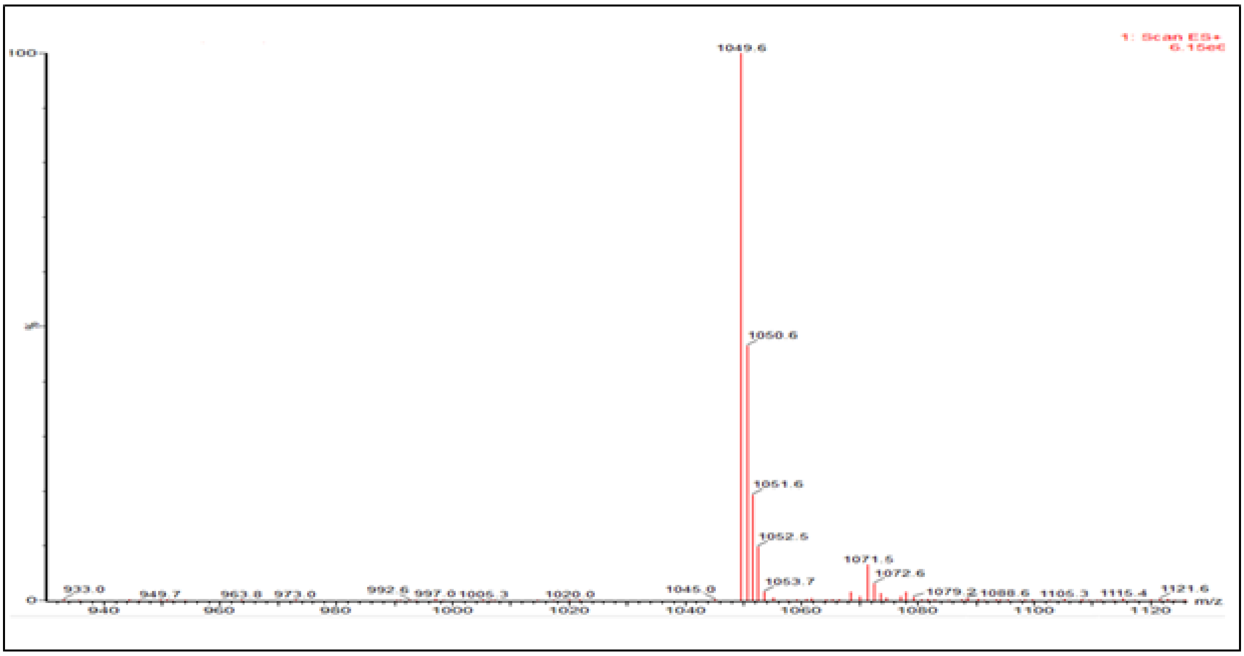

HPLC chromatogram of crude compound **24c** and corresponding mass spectrum

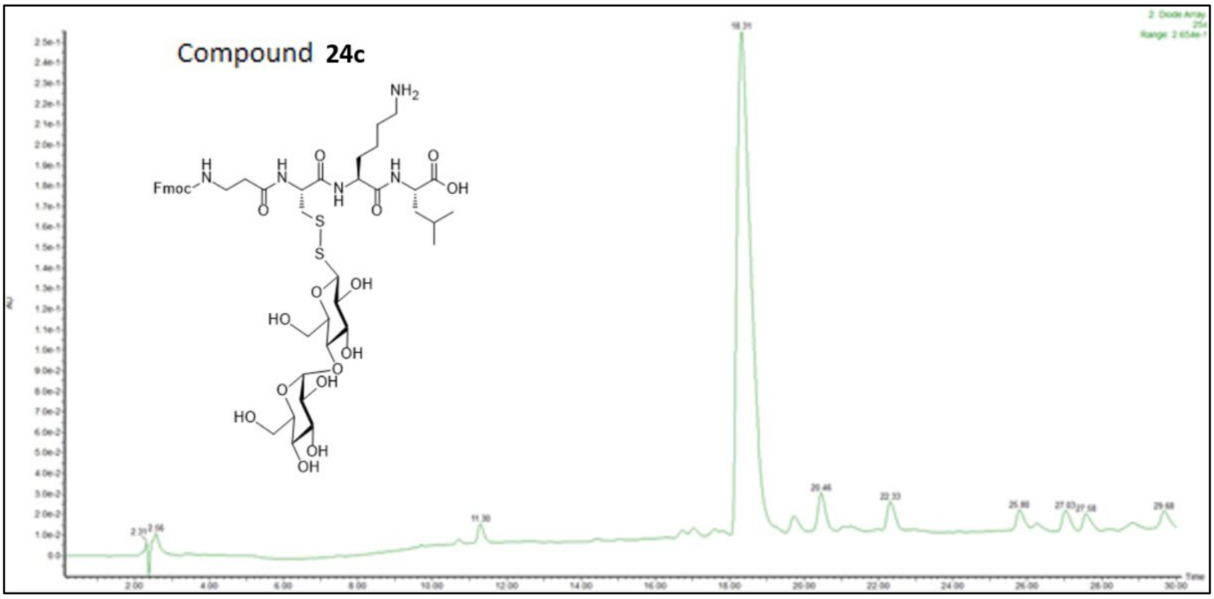

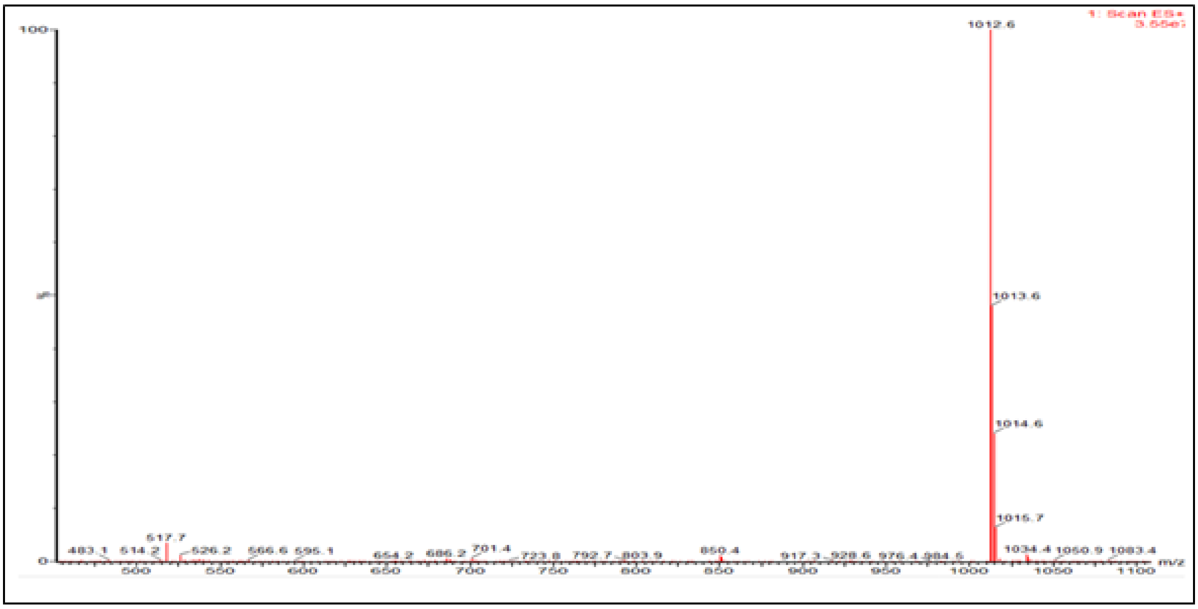

HPLC chromatogram of crude compound **24g** and corresponding mass spectrum

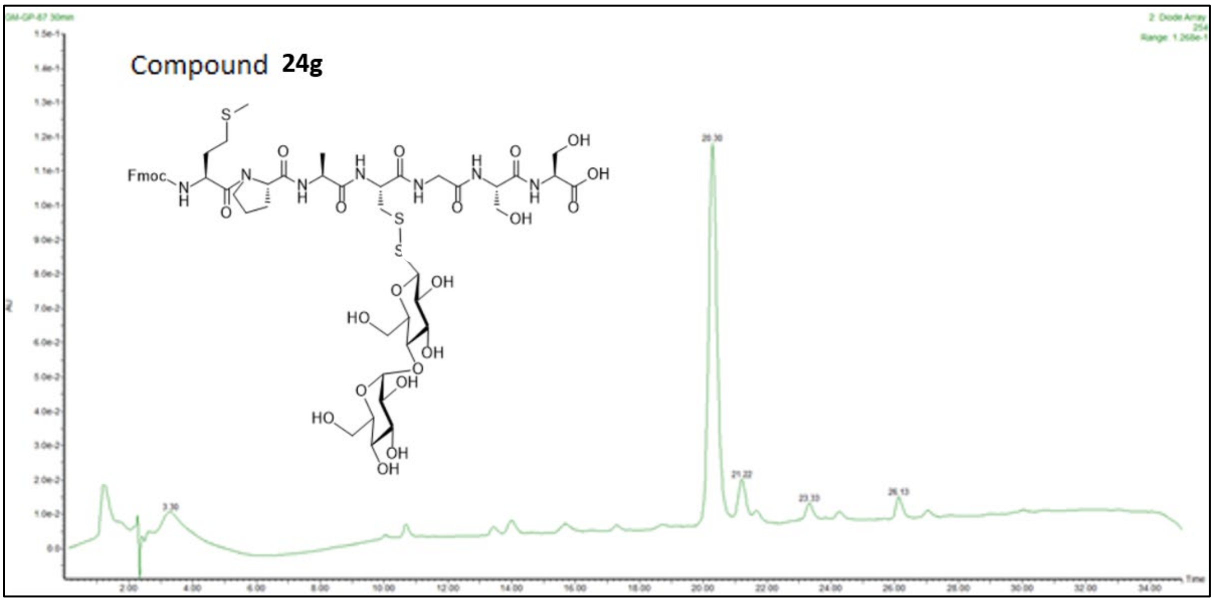

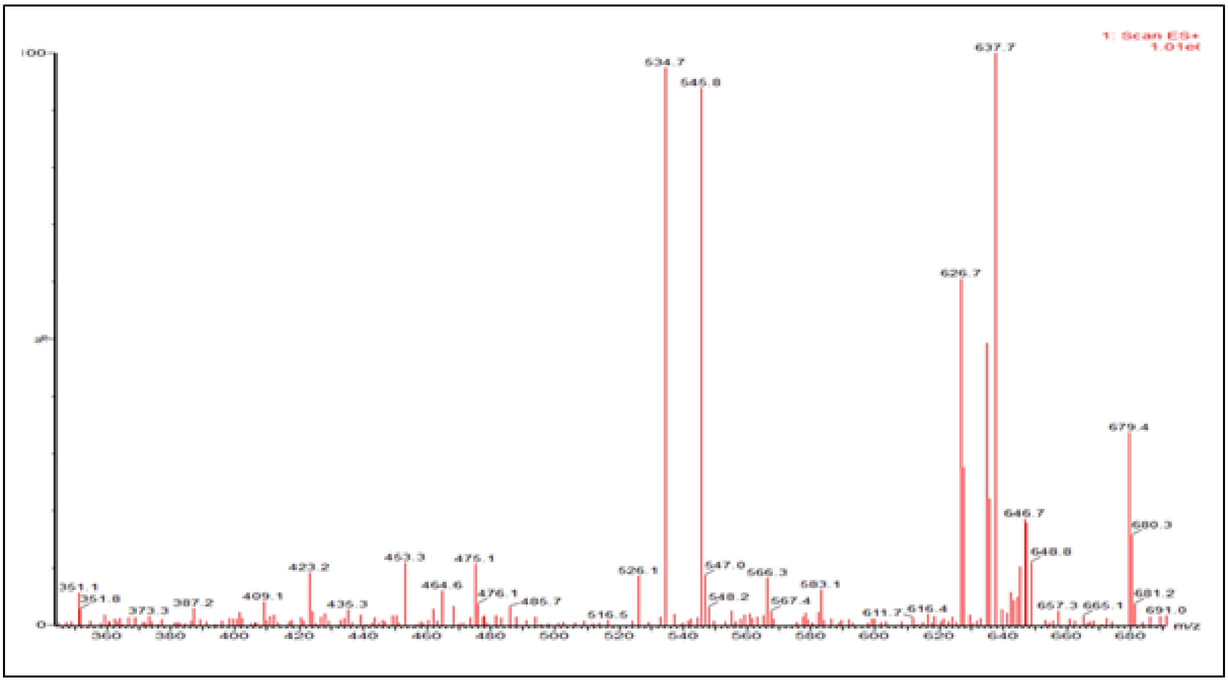

HPLC chromatogram of crude compound **24h** and corresponding mass spectrum

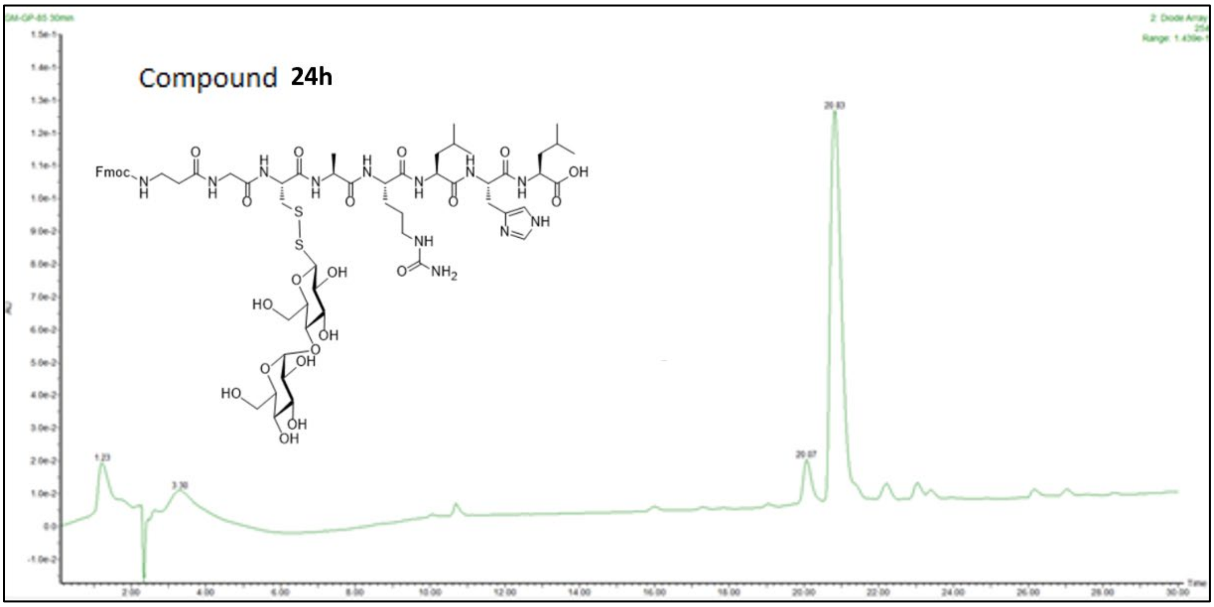

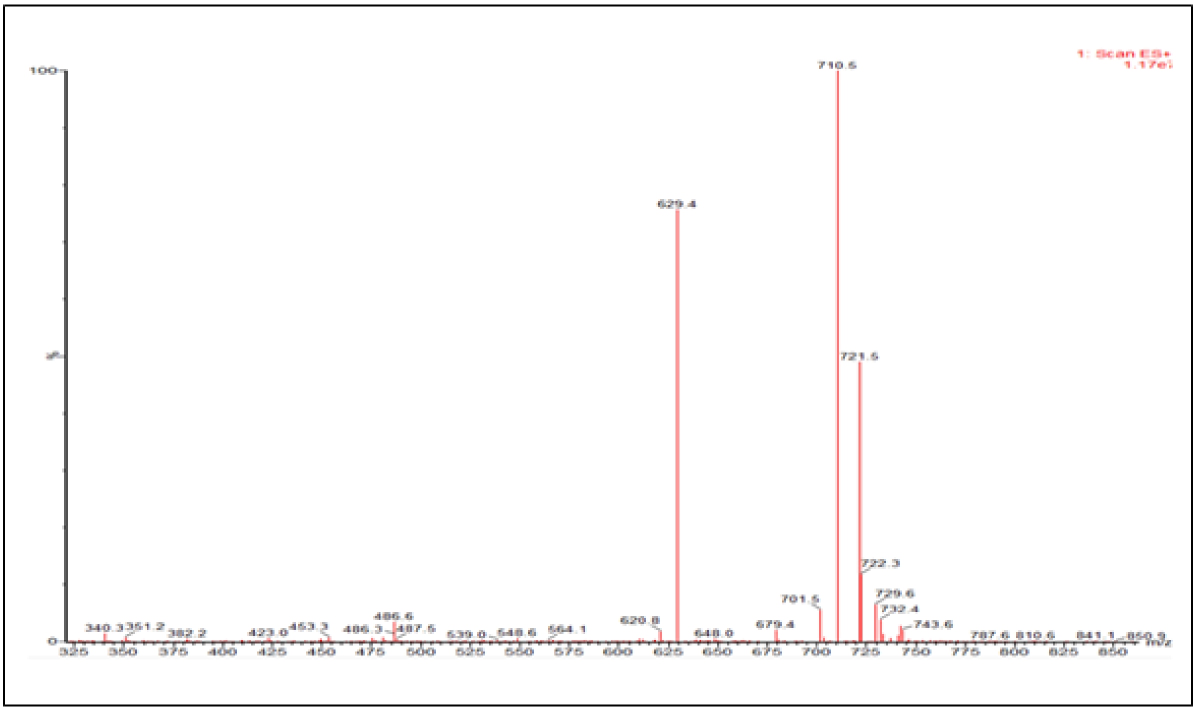

HPLC chromatogram of crude compound **24i** and corresponding mass spectrum

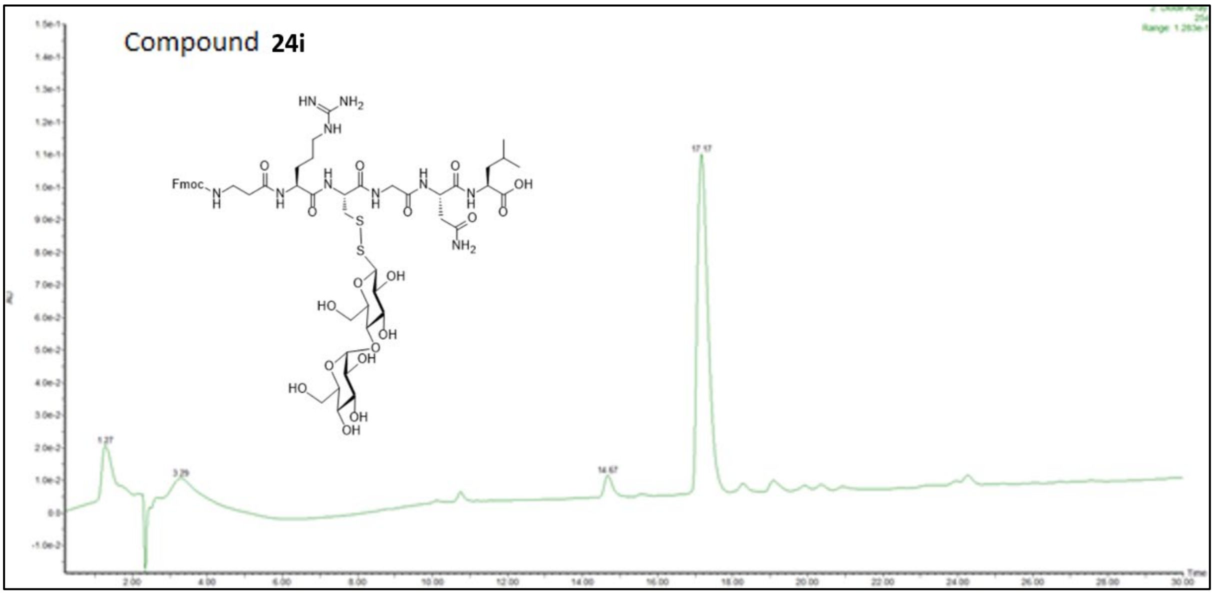

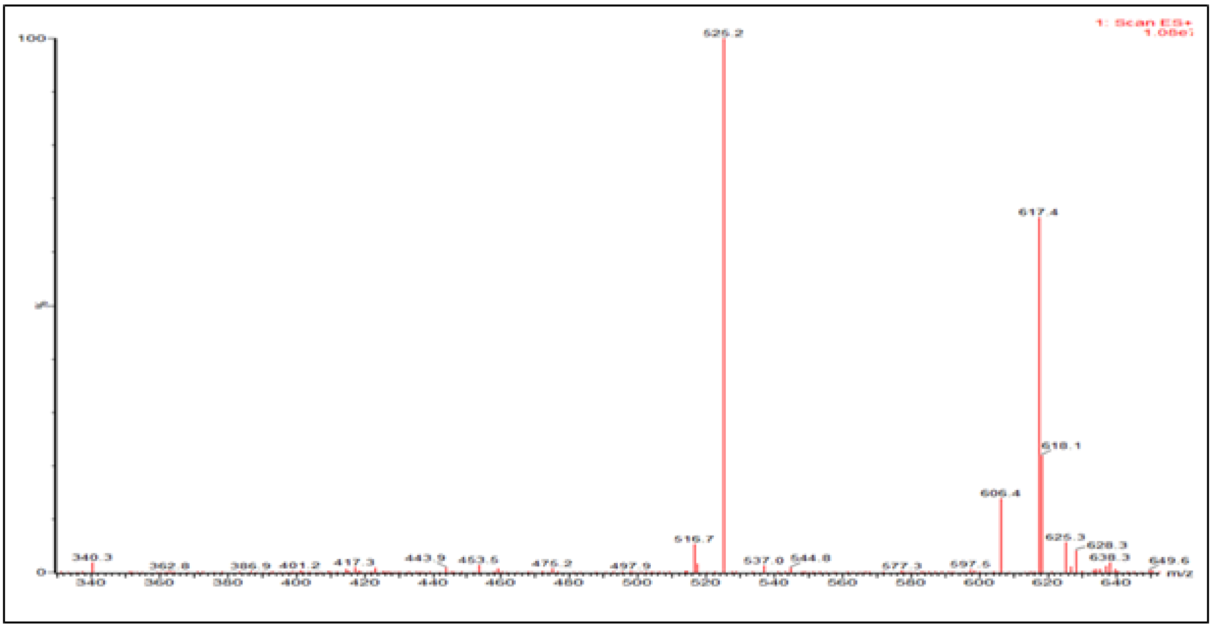

HPLC chromatogram of crude compound **25c** and corresponding mass spectrum

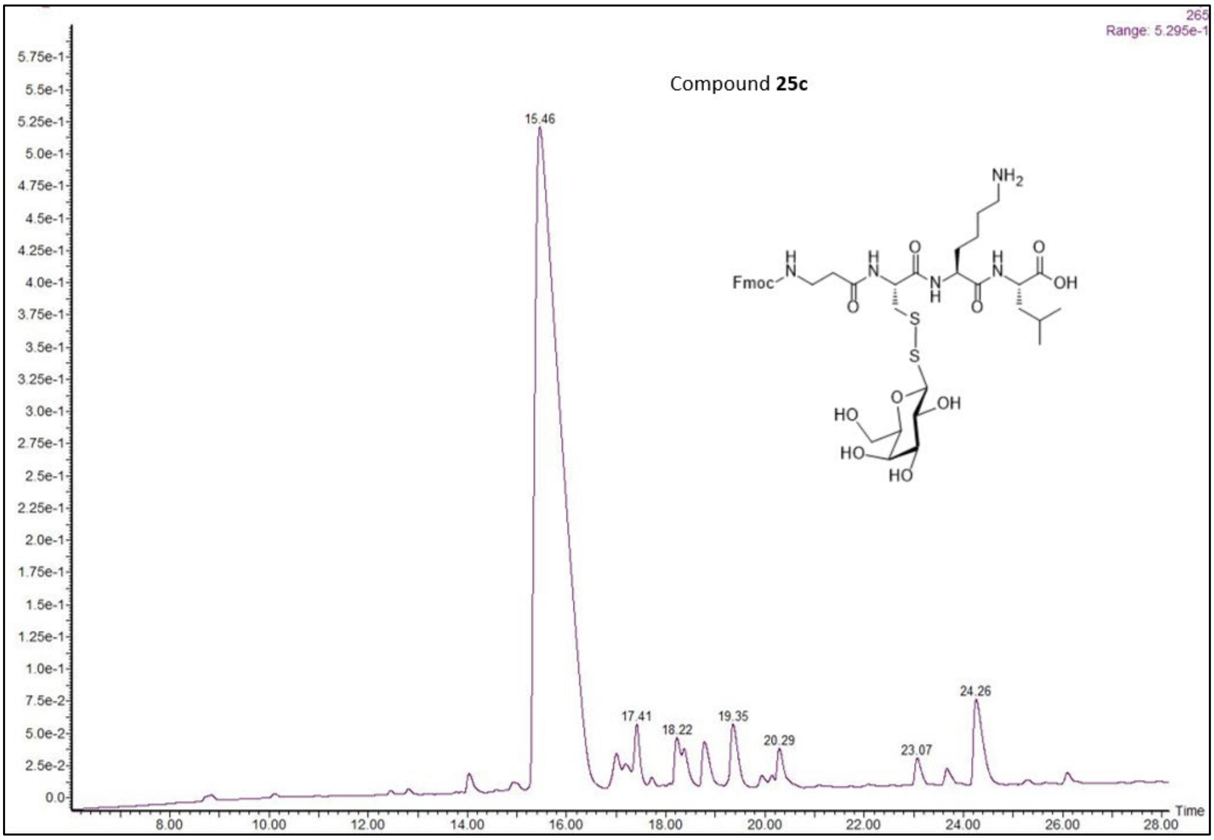

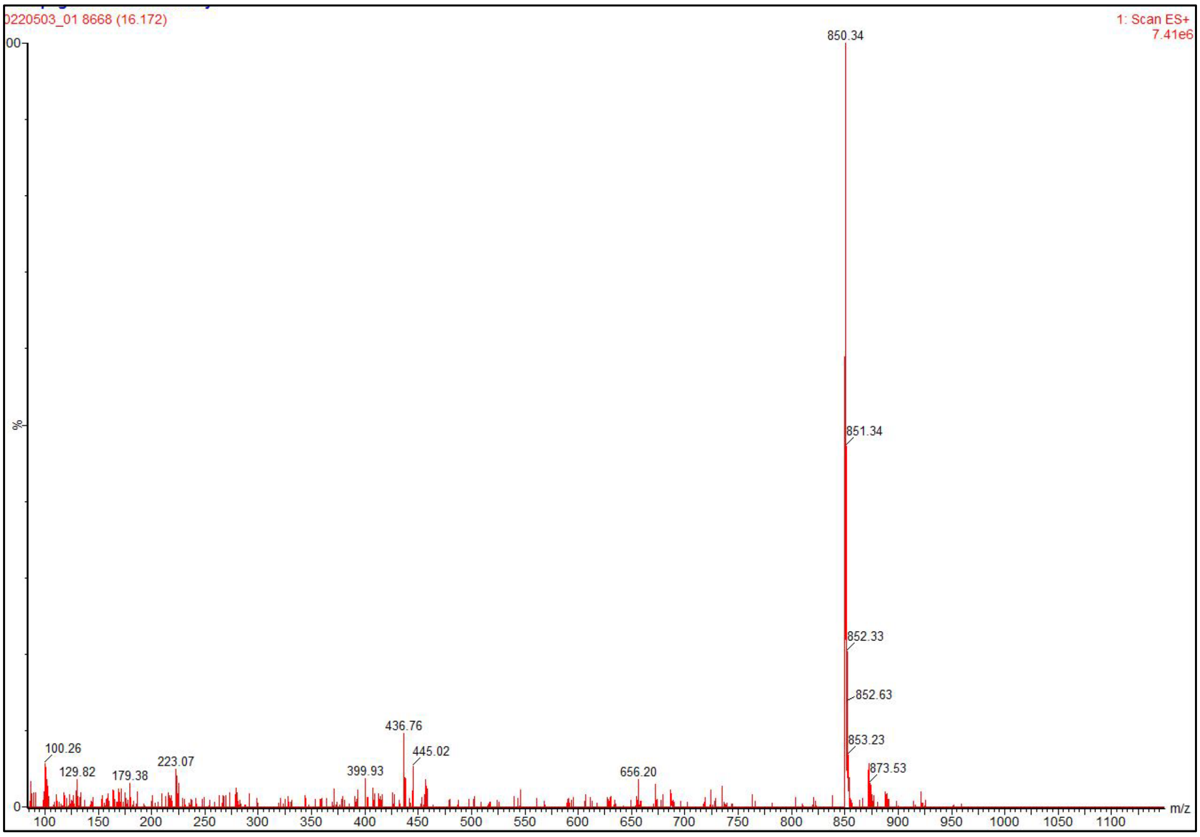

HPLC chromatogram of crude compound **25g** and corresponding mass spectrum

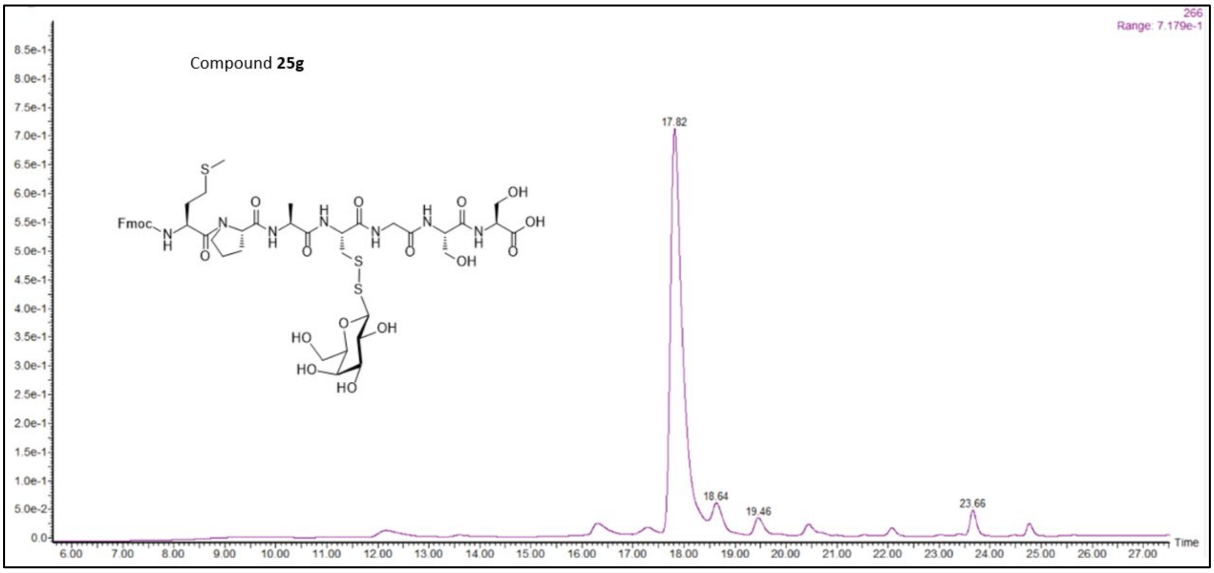

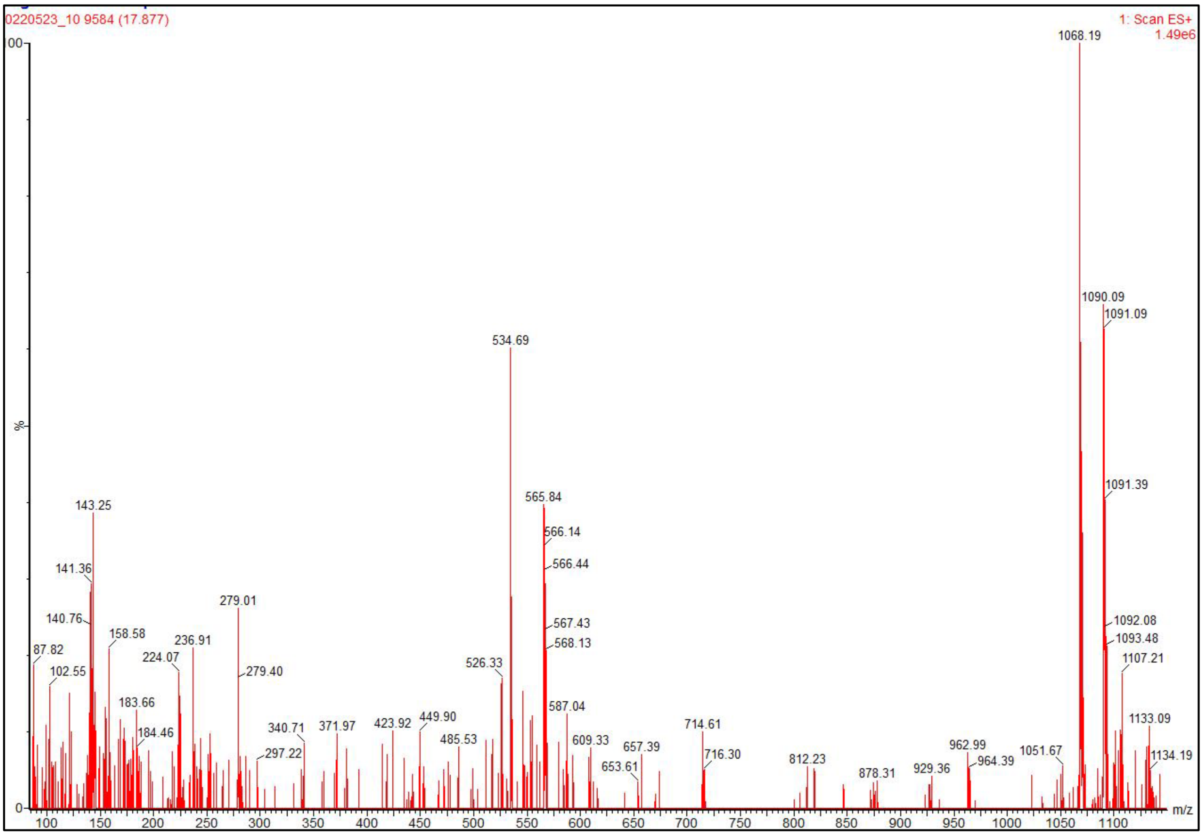

HPLC chromatogram of crude compound **25h** and corresponding mass spectrum

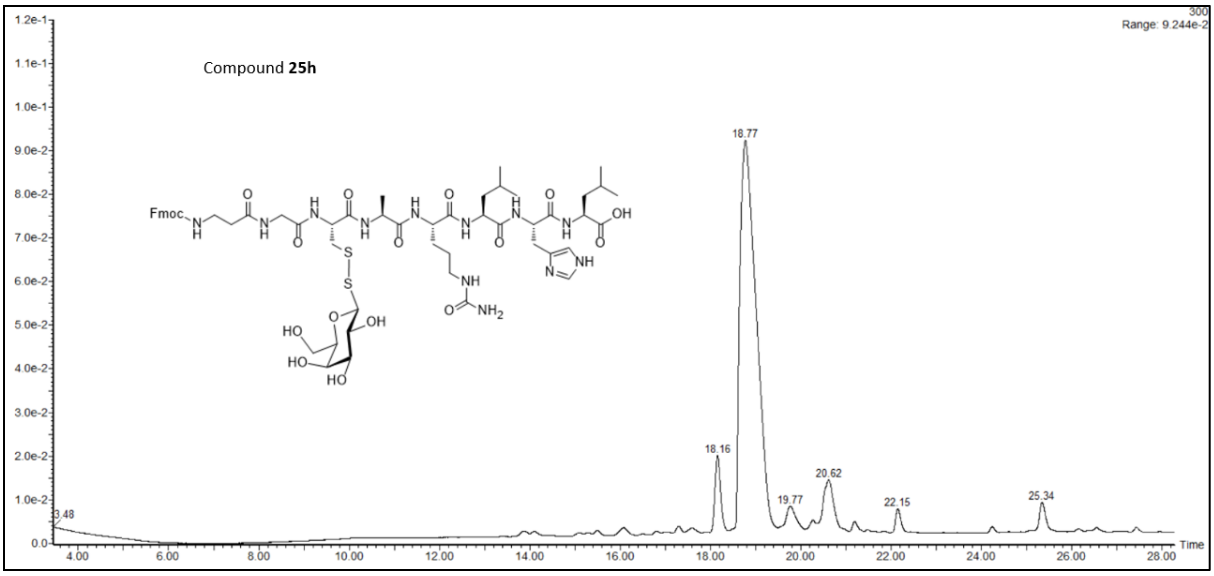

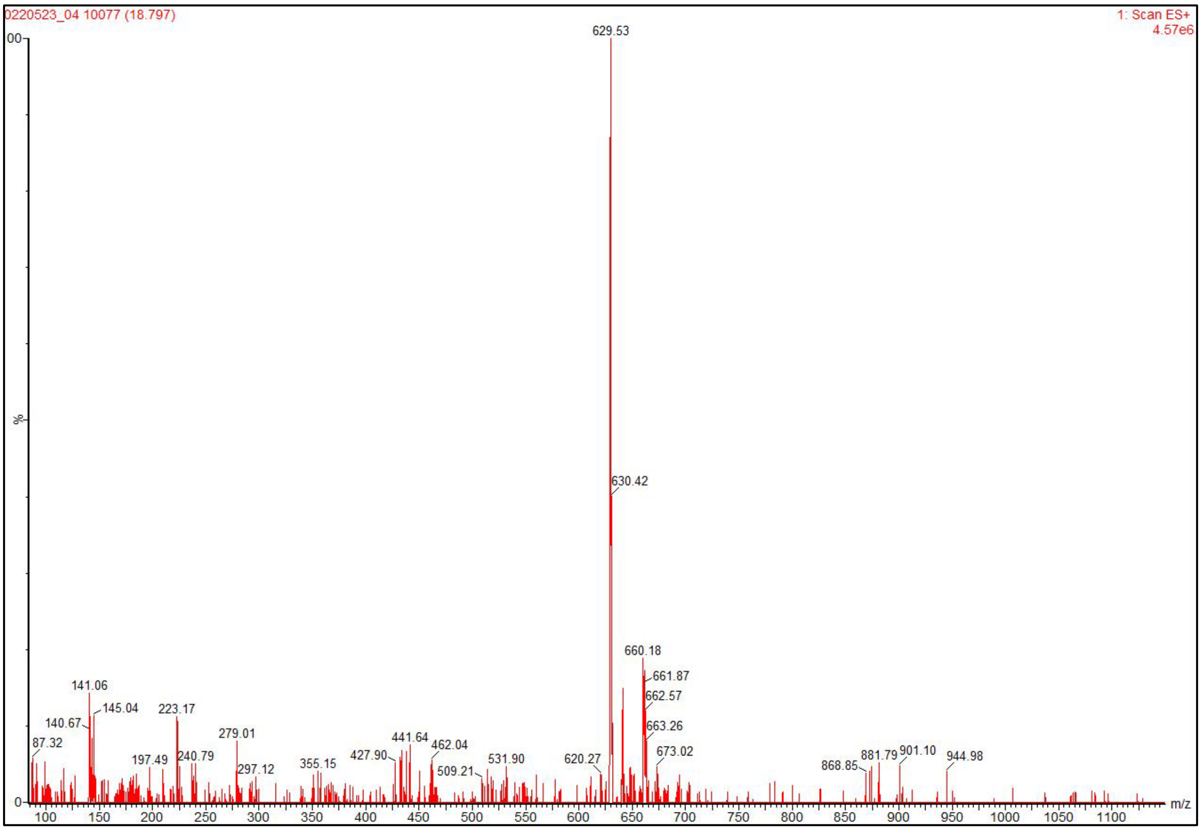

HPLC chromatogram of crude compound **25i** and corresponding mass spectrum

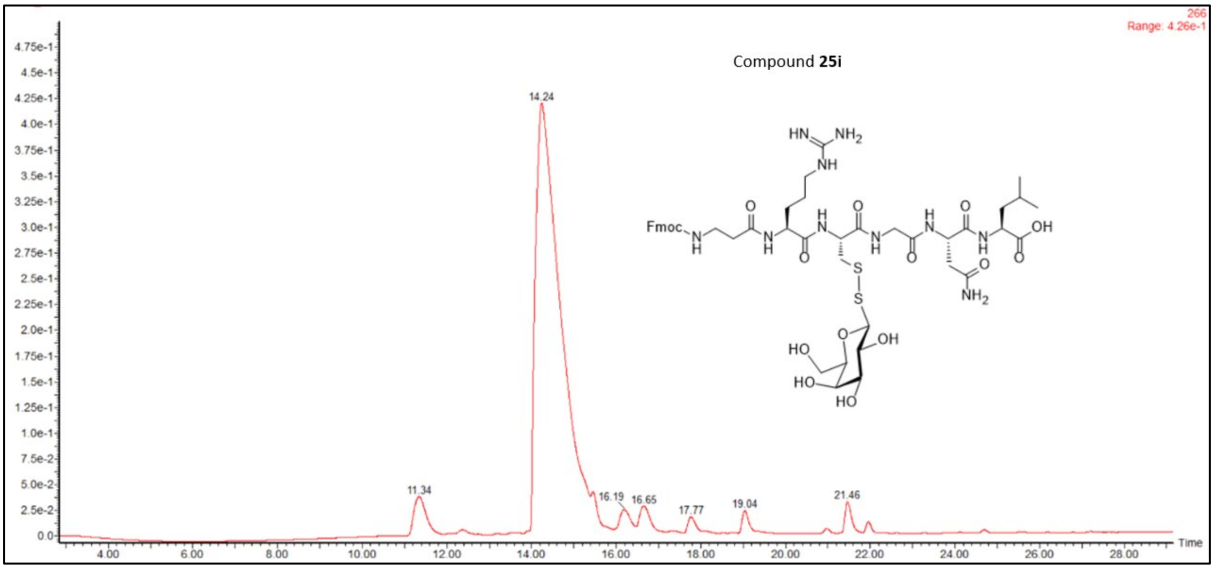

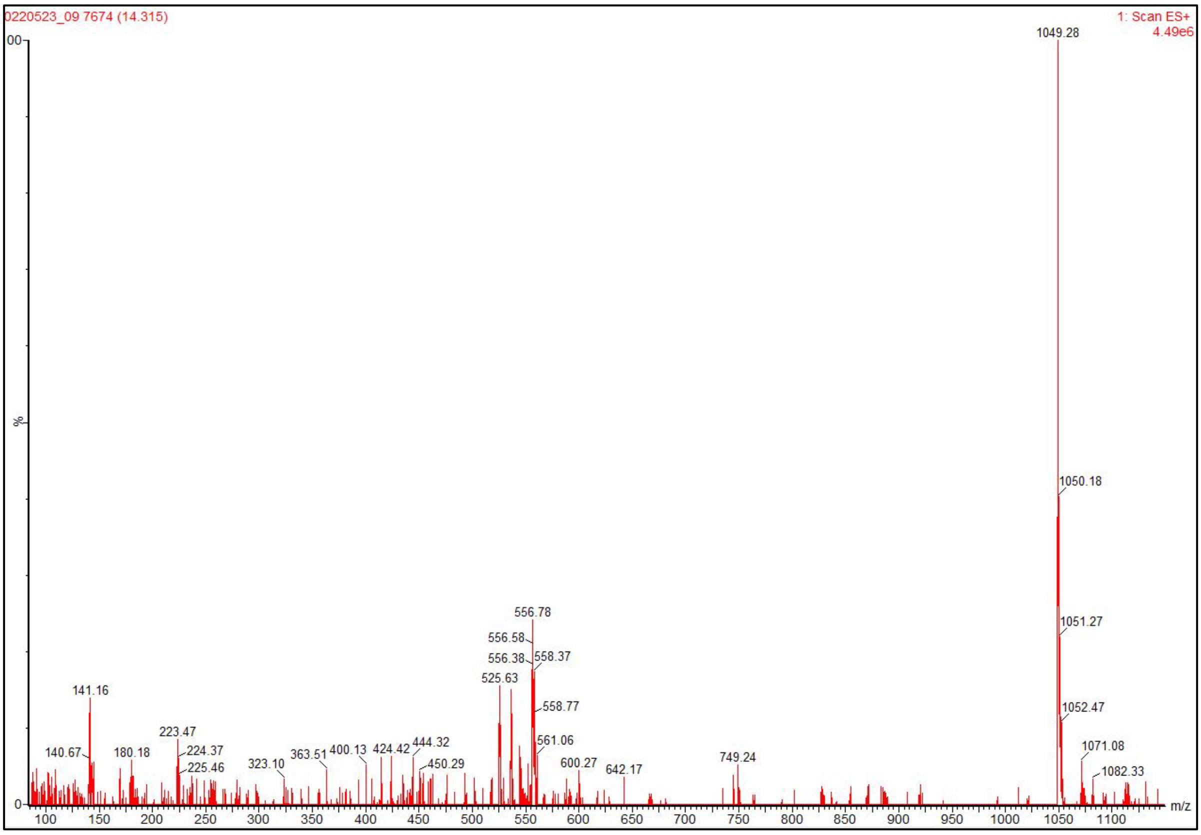

HPLC chromatogram of crude compound **26c** and corresponding mass spectrum

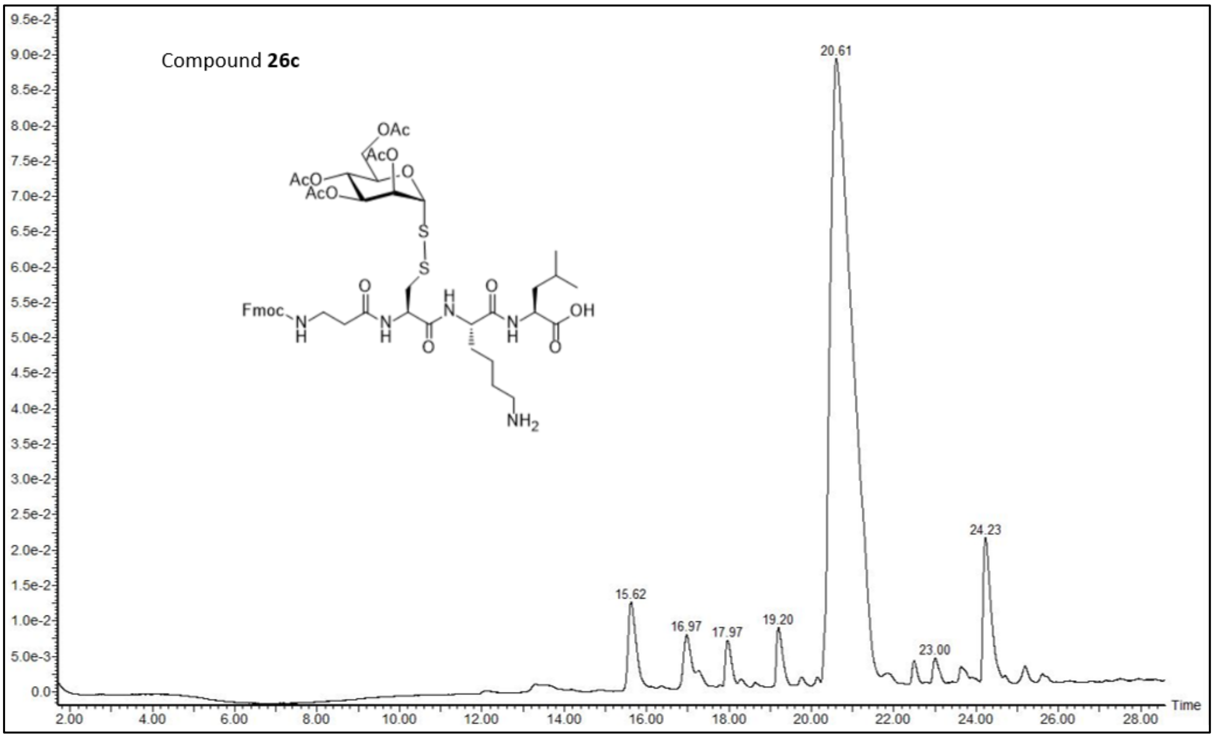

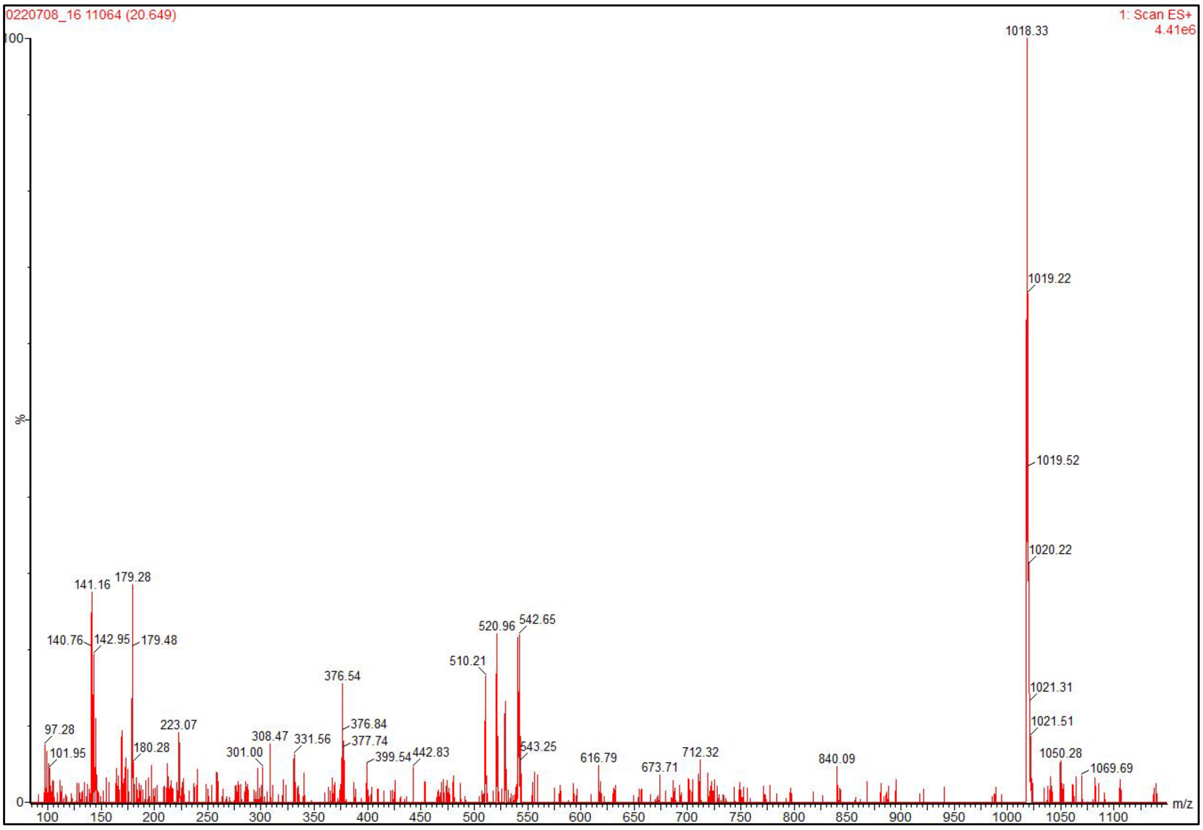

HPLC chromatogram of crude compound **26g** and corresponding mass spectrum

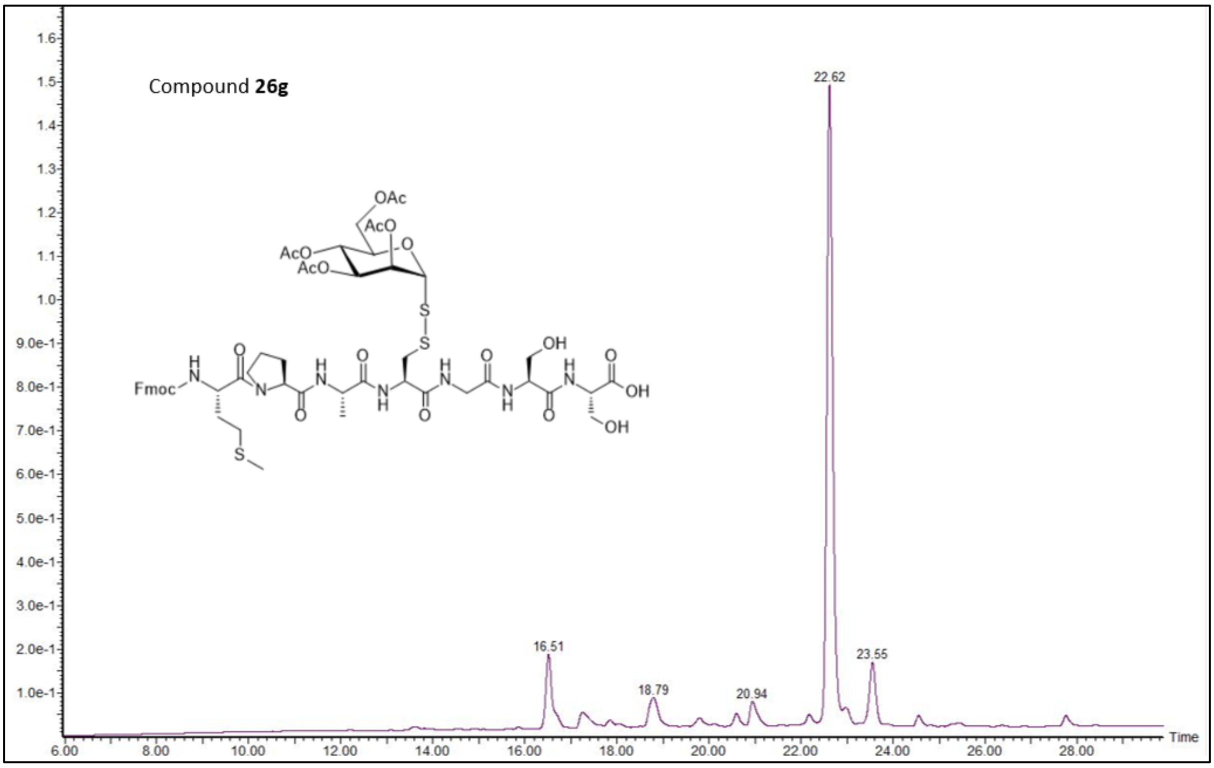

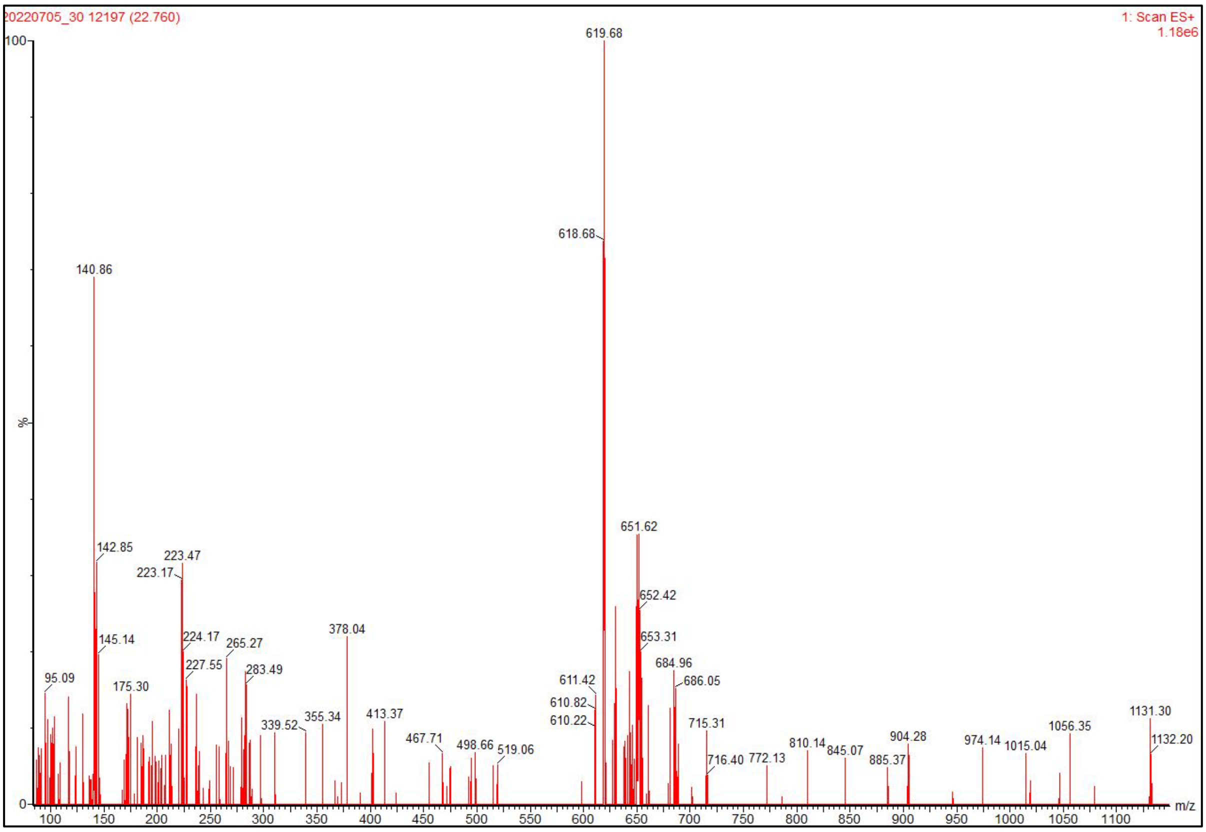

HPLC chromatogram of crude compound **26i** and corresponding mass spectrum

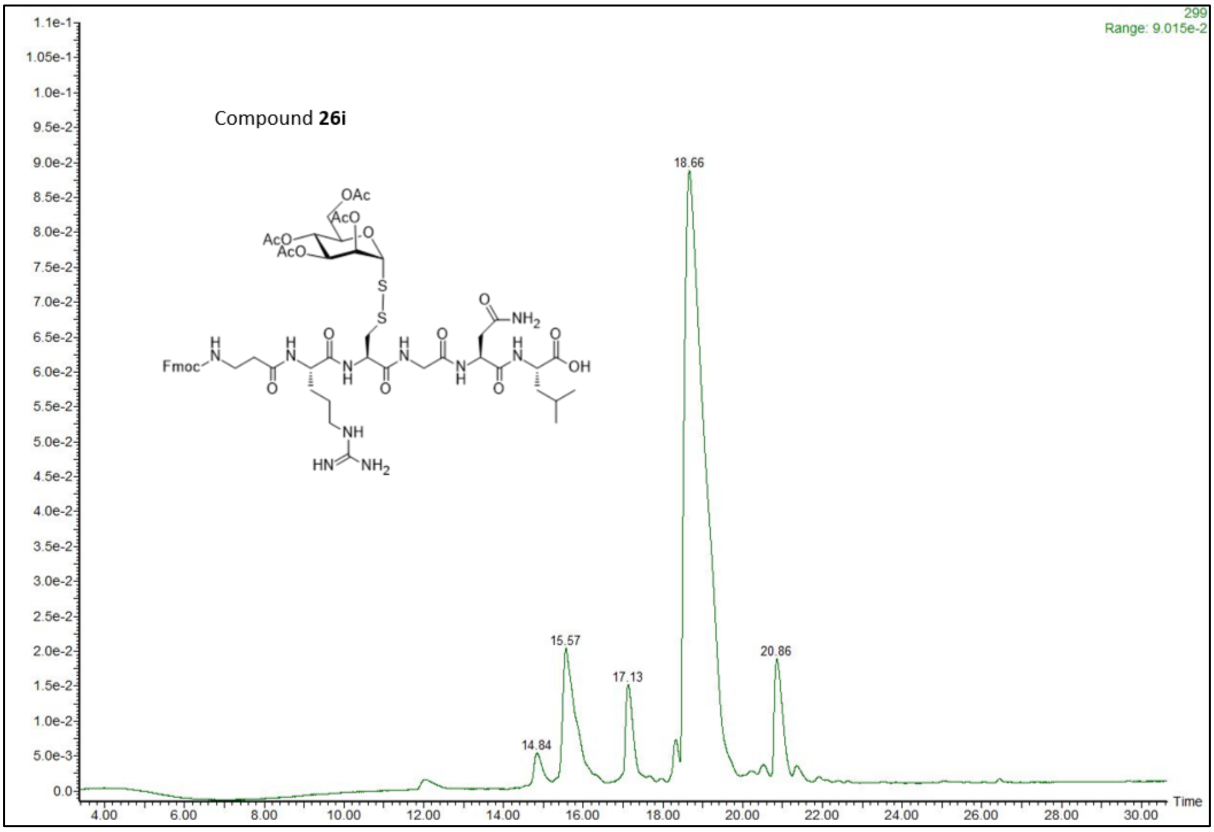

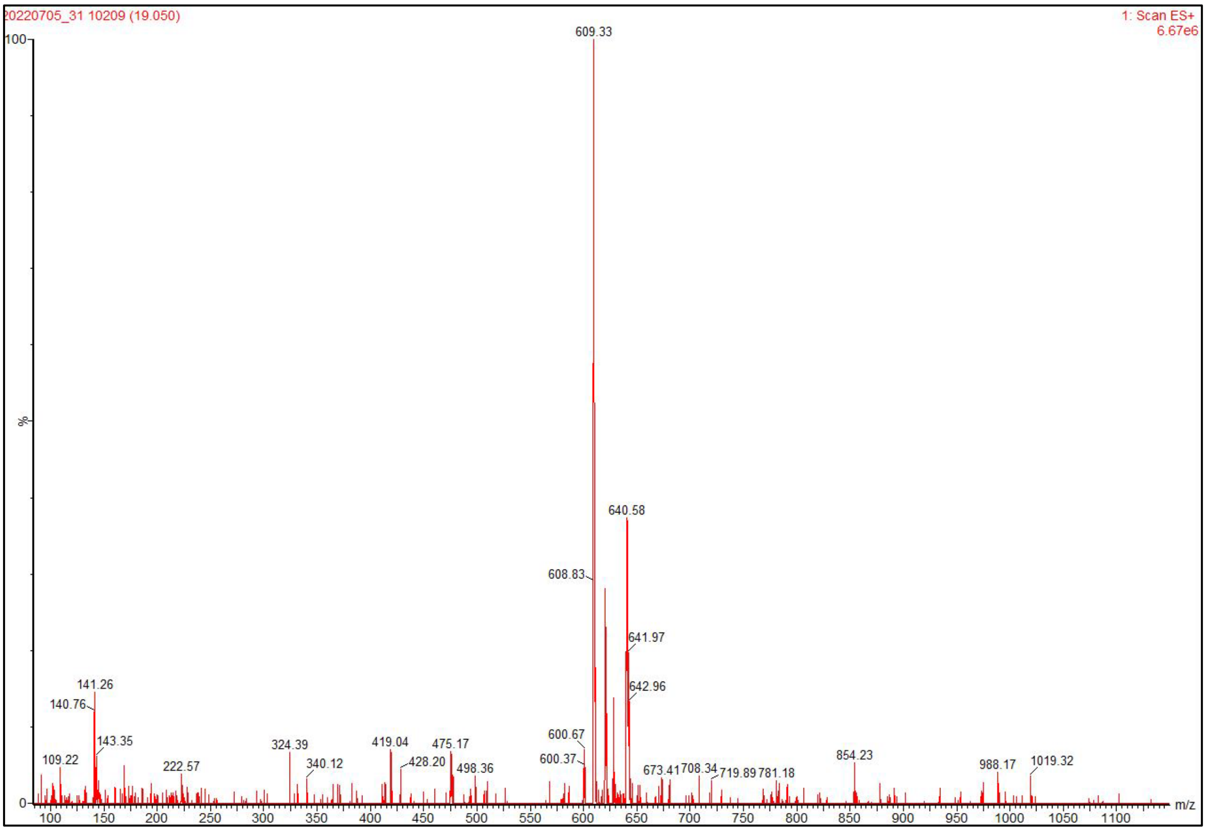

Supplement: Supplementary file 1 — jo2c01651_si_001.pdf [file jo2c01651_si_001.pdf]
